# Ultra-flexible optoelectronic stimulator converts tissue-attenuated weak light into electrical signals for cardiac remodeling

**DOI:** 10.1038/s41467-026-75495-7

**Published:** 2026-07-13

**Authors:** Ruiyue Zhao, Feifan Yang, Tao Yang, Kanghua Li, Ruisi Gao, Xinchang Kang, Qi Chen, Liang Li, Jia Chen, Yi Yuan, Xinyi Chen, Mengxue Zhou, Shengmin Zhang, Jianglin Wang, Chuanbin Mao

**Affiliations:** 1 Department of Biomedical Engineering, Huazhong University of Science and Technology, Wuhan, China; 2 School of Integrated Circuits, Huazhong University of Science and Technology, Wuhan, China; 3 Department of Biomedical Engineering, The Chinese University of Hong Kong, Hong Kong, China; 4 Shenzhen Huazhong University of Science and Technology Research Institute, Shenzhen, China

**Keywords:** Biomedical engineering, Implants

## Abstract

Precise spatiotemporal resolution and wireless multi-site modulation of excitable tissues via optoelectronics offers transformative potential for bioelectronic medicine, yet clinical translation is hindered by the rigidity of conventional silicon-based devices and their reduced performance under tissue-attenuated illumination, where the weak light reaching implants often fails to generate sufficient stimulation voltage. Here, we report an ultra-flexible, high-efficiency optoelectronic stimulator (OES) based on (Bi,Sb)_2_Se_3_, a semiconductor with crystal structure comprising parallel 1D chains that enable efficient flexibility and photocarrier transport. The OES achieves robust photoelectric conversion under near-infrared light intensities as low as 0.55 μW cm^−2^, reaching quantum efficiency of up to 89.60% while conforming seamlessly to soft tissues. In a rat model of myocardial infarction, the OES restored electrical conduction across infarcted regions and improved cardiac function under weak-light stimulation. Scalable fabrication yields large-area devices without loss of performance, as validated in a swine model. This work introduces a clinically translatable optoelectronic platform for soft-tissue modulation under low-power light, establishing a foundation for next-generation, minimally invasive cardiac repair technologies.

## Abstract (web summary)

Cardiac repair after myocardial infarction is limited by impaired electrical conduction. Here, the authors report a flexible optoelectronic stimulator that restores cardiac conduction and improves function under infrared light.

## Introduction

Myocardial infarction (MI), a leading cause of heart failure, results in irreversible cardiomyocyte loss and fibrotic remodeling that disrupts electrical conduction and impairs cardiac function^1–4^. Electrical stimulation has emerged as a promising therapeutic strategy for re-establishing conduction and improving contractile synchrony^5–9^. However, conventional approaches, from wired metal electrodes to wireless piezoelectric devices, face intrinsic limitations^10–13^. Wired electrodes are bulky, lack tunability, and rely on finite battery life^14–16^, while piezoelectric stimulators, typically driven by external ultrasound, offer limited spatiotemporal resolution and are incapable of precise, multi-site modulation^17^.

Recent breakthroughs in optoelectronic stimulation offer wireless, spatially resolved, and temporally precise modulation of cardiac tissue, enabling synchronized multi-site pacing and presenting a compelling platform for functional cardiac repair^3,18–22^. However, clinical translation of implantable optoelectronic stimulators remains hindered by fundamental materials and engineering challenges. Conventional photovoltaic materials, such as silicon-based semiconductors^23,24^, are intrinsically rigid and mechanically incompatible with the dynamic environment of biological tissues^10,25–27^. Additionally, efficient light-to-electricity conversion under the highly attenuated weak-light conditions of biological tissue remains an unsolved barrier^18,28–30^.

Antimony selenide (Sb_2_Se_3_), a one-dimensional (1D) inorganic semiconductor, has garnered growing utility in photovoltaics and photodetectors owing to intrinsic flexibility and superior photoelectric conversion efficiency under weak-light conditions^24,31–33^. Despite these advantageous properties, its potential in bioelectronic systems remains unexplored. Here, we report a mechanically compliant, optoelectronically tunable stimulator based on (Bi,Sb)_2_Se_3_ semiconductors for wireless, passive cardiac modulation. Bi incorporation enhances charge separation and photocurrent generation, achieving efficient light-driven voltage outputs and frequency modulation via pulsed illumination^33,34^.

**Fig. 1:**
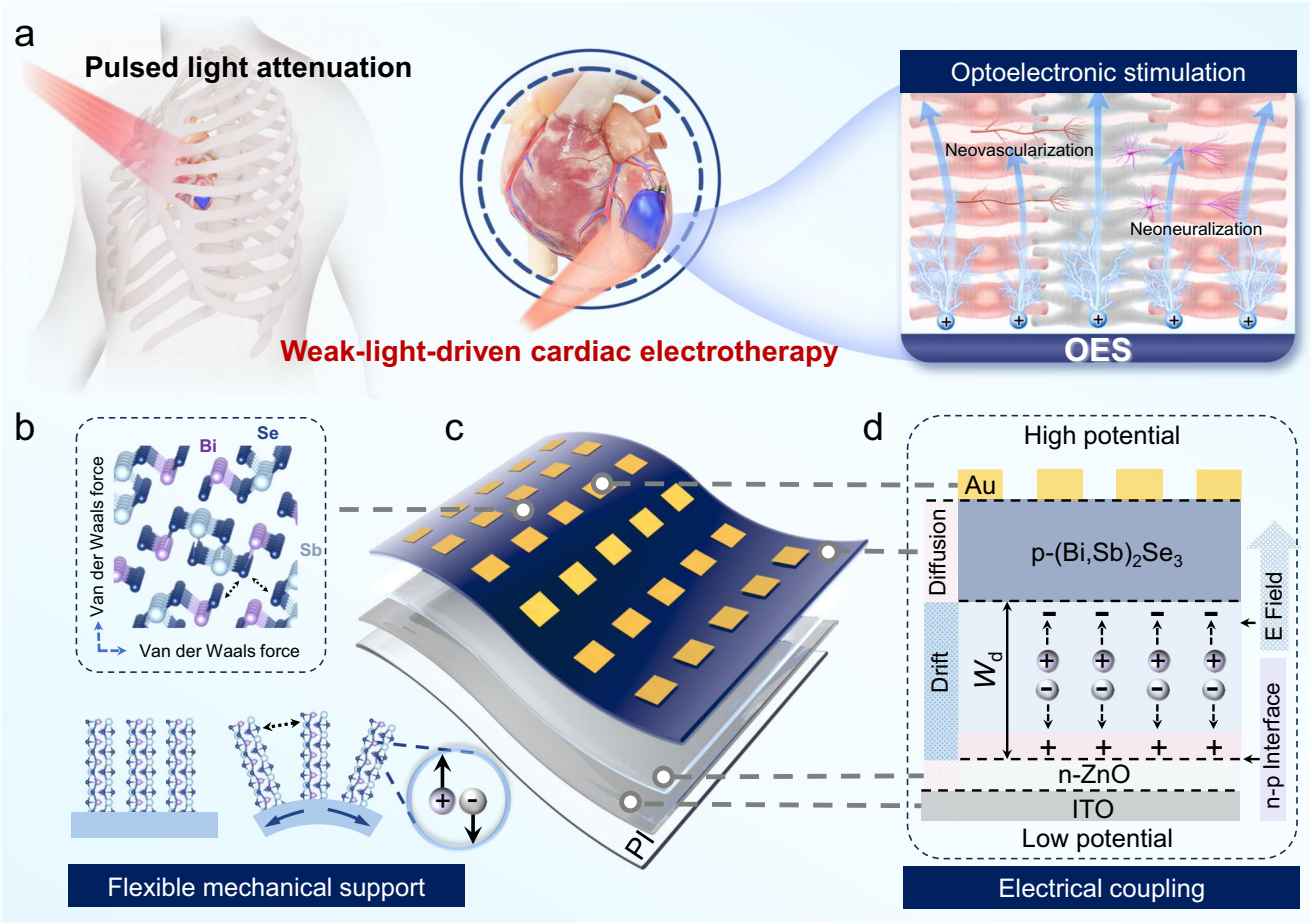
Design concept of the optoelectronic stimulator (OES). The OES is designed to achieve safe and effective deep-tissue optoelectronic stimulation under weak light illumination. **a** Schematic of the therapeutic concept and working mechanism. Pulsed light penetrates biological tissues and attenuates to weak intensity, yet remains sufficient to activate the OES. The device converts incident light into localized electrical stimulation through photovoltaic charge generation and separation, thereby promoting neurogenesis, neovascularization, and cardiomyocyte remodeling at infarction sites while avoiding phototoxicity to healthy cardiomyocytes. **b** Schematic of the mechanical adaptability of the OES. Owing to its one-dimensional (1D) inorganic semiconductor structure and weak interlayer molecular van der Waals interactions, the device can conformally integrate with dynamically beating cardiac tissues, thereby improving stimulation stability. **c**, **d** Structural design and working principle of the OES. The device consists of a flexible polyimide (PI) substrate layered with infrared-transparent indium tin oxide (ITO), n-type zinc oxide (n-ZnO), a photovoltaic p-(Bi,Sb)_2_Se_3_ layer, and Au electrodes. The ITO/ZnO/(Bi,Sb)_2_Se_3_ layers form a continuous multilayer stack, while only the top Au electrode is patterned into an array of addressable cells. The stacked functional layers are in continuous interfacial contact without physical separation within the device structure. The built-in electric field enables efficient photocarrier generation and separation, allowing effective light-to-electricity conversion even under weak illumination for deep-tissue applications.

The resulting device exhibits robust biocompatibility and negligible foreign body response. In vitro, optical stimulation of neonatal rat cardiomyocytes using the device elicited synchronized, high-frequency calcium transients and improved intercellular coupling. In a rat model of myocardial infarction, stimulation promoted electrical conduction and functional recovery. Importantly, scalable fabrication yields large-area devices without loss of optoelectronic performance as validated in a mature swine model, promoting real-time modulation of heart rate under clinically relevant light intensities. By integrating soft mechanics with efficient low-intensity light harvesting, this platform establishes a foundational strategy for wireless, light-controlled cardiac modulation and presents a clinically translatable approach for post-infarction cardiac repair (Fig. 1).

## Results

### Photovoltaic properties of the optoelectronic stimulator (OES)

**Fig. 2:**
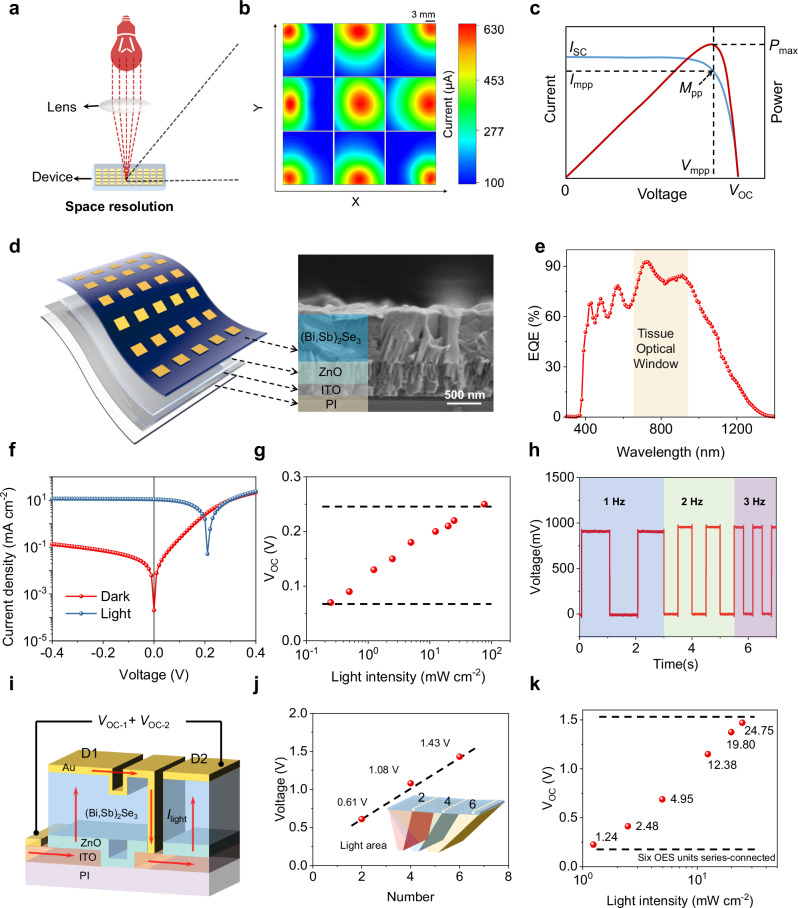
Photovoltaic properties of optoelectronic stimulator (OES). **a**, **b** 2D photocurrent mapping under 780 nm LED illumination using a localized light spot approximately 4.5 mm in diameter. The device was sequentially illuminated at nine distinct positions across the 3 × 3 electrode array. The nine panels correspond to nine illumination positions ordered left-to-right, top-to-bottom. **c** Schematic *I*-*V* and *P*-*V* plot illustrating the self-powered behavior of the device. *I*_SC_: short-circuit current; *V*_OC_: open-circuit voltage; *M*_PP_: maximum power point; *V*_mpp_/*I*_mpp_: voltage/current at maximum power point; *P*_max_: maximum output power. **d** Cross-sectional schematic showing the multilayer bottom-up architecture. Highly textured [*hk*1] polycrystalline (Bi,Sb)_2_Se_3_ layer with vertically oriented columnar grains to enhance carrier transport. **e** Spectral response of the OES across the near-infrared window (650–900 nm), ensuring efficient tissue penetration. **f**, **g** At 780 nm and 24.7 mW cm^−2^, a single OES unit generates a peak open-circuit voltage (*V*_OC_) of 0.22 V, which can be modulated from 0.02 to 0.22 V by tuning light intensity. **h** Time-resolved *V*oc traces showing rapid response under pulsed illumination at 1, 2, and 3 Hz. **i** Schematic of OES mini-modules in series for voltage tuning. The series interconnection is implemented during fabrication, and electrical measurements are taken from the two end terminals. **j** Voltage increases with the number of series-connected modules under a constant 780 nm light intensity of 24.7 mW·cm^−2^. **k** *V*_OC_ of a series-connected module with six cells as a function of incident light intensity. The value at each point indicates the corresponding *X*-axis light intensity.

The flexible OES was fabricated by sequentially depositing ITO, n-type ZnO, and p-type (Bi,Sb)_2_Se_3_, followed by Au electrode, onto polyimide (PI, 8 μm-thick) film pre-formed on glass. The completed device was then peeled off from the glass substrate to obtain a freestanding flexible OES enabling conformal integration with soft biological tissues (Figure S1). Two-dimensional photocurrent mapping under 780 nm illumination from multiple orientations reveals the excellent spatial resolution of OES (Fig. 2a, b, detail see “Methods”). At the core of the device, the ZnO/(Bi,Sb)_2_Se_3_ interface forms a p-n junction, where a built-in electric field drives efficient separation of photogenerated carriers. This results in a photovoltage between the Au (high potential) and ITO (low potential) electrodes, enabling localized, passive stimulation of adjacent cells and tissues (Fig. 2c).

Cross-sectional SEM imaging revealed a compact, nearly vertically aligned [*hk*1]-oriented grain structure in (Bi,Sb)_2_Se_3_, which supports efficient carrier transport and underpinned the device’s photoelectric performance (Figs. 2d, S3, and 4a). The OES exhibited a broadband spectral response spanning 365–1400 nm (Fig. 2e), encompassing the biological optical window (650–900 nm) associated with deep tissue penetration^35^. Within this window, the device achieved an external quantum efficiency (EQE) exceeding 80%, underscoring its high photoelectric conversion efficiency. The device was stimulated using a single-wavelength (780 nm) light source. The applied illumination intensity (0.05–24.7 mW cm^−2^) falls within the commonly used photobiomodulation range (~ 1–100 mW cm^−2^), which is generally considered safe and non-thermal for biological tissues^36^. Under 780 nm illumination at 24.7 mW cm^−2^, the device generated an open-circuit voltage of 0.22 V (Fig. 2f), sufficient for bioelectronic activation. The output voltage was tunable between 0.02 V and 0.22 V by varying the incident intensity from 0.05 to 24.7 mW cm^−2^ (Figs. 2g and S4c). Infrared thermal imaging further confirmed negligible photothermal effects, with only ~0.5 °C temperature variation under maximum illumination for 1 h (Fig. S5), well below reported thermal damage thresholds (> 600 mW cm^−2^)^37^. Moreover, the OES exhibited rapid response kinetics, supporting frequency modulation up to 3 Hz (Figs. 2h and S4d), precisely controlling stimulation dynamics in biological systems.

To extend output voltage and support broader applications, a modular architecture was developed by integrating multiple OES units in series (Figs. 2i, S6, and S7). Increasing the number of modules yielded stepwise voltage outputs of 0.61, 1.08, and 1.43 V for 2, 4, and 6 units, respectively (Fig. 2j). Across light intensities ranging from 1.24 to 24.7 mW cm^−2^, the system provided tunable voltages up to 1.47 V (Fig. 2k, and S4e, f), enabling adaptive control of electrical output for applications requiring dynamic, intensity-responsive bioelectronic stimulation.

### Mechanical compliance and durability of the OES

**Fig. 3:**
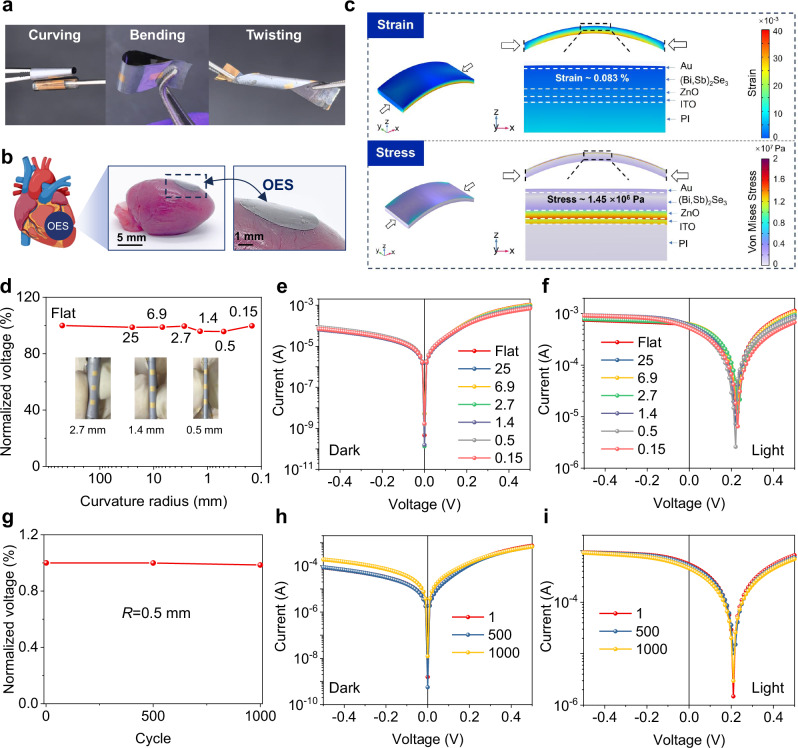
Mechanical flexibility and durability of the optoelectronic stimulator (OES). **a** Demonstration of mechanical compliance under various deformation modes, including curving, bending, and twisting. **b** The OES conforms tightly to cardiac tissue, exhibiting robust adhesive properties. Created in BioRender. ZHAO, R. (2026) https://BioRender.com/y3hfpkw. **c** Finite element analysis of the OES under bending, showing strain and von Mises stress distributions across individual functional layers (Au: 0.08 μm; (Bi,Sb)_2_Se_3_: 0.8 μm; ZnO: 0.34 μm; ITO: 0.2 μm; PI: 8 μm). **d** Normalized output voltage remains stable after repeated bending at curvature radii ranging from 25 to 0.15 mm. **e**, **f** *I-V* curves under dark and 780 nm illumination confirm preserved device performance across curvature radii. **g** The normalized output voltage exhibits negligible attenuation after 500 and 1000 bending cycles at 0.5 mm radius. **h**, **i** Corresponding *I-V* curves after 1000 cycles show a minor increase in reverse saturation current, while output voltage remains unchanged, indicating strong mechanical durability and functional integrity.

Fabricated on an optimized polyimide (PI) substrate, the integrated OES demonstrated excellent flexibility, accommodating complex deformations including bending, twisting, and folding without mechanical failure (Fig. 3a). When applied to the epicardial surface in vitro, the device conformed intimately to the tissue and delivered localized electrical signals with high spatial precision to the infarcted myocardium, an essential prerequisite for effective bioelectronic pacing (Fig. 3b)^38^.

The mechanical resilience of the OES arises from the intrinsic van der Waals bonding and benign grain boundaries within the one-dimensional crystal framework of (Bi,Sb)_2_Se_3_, as verified by TEM (Fig. S3), which confer exceptional tolerance to deformation and structural imperfections^31,39^. Upon bending, the (Bi,Sb)_2_Se_3_ layer experienced an in-plane strain of ~0.083%, corresponding to a stress of ~1.45 × 10^6^ Pa, significantly lower than that in conventional three-dimensional semiconductors such as ZnO and ITO (Fig. 3c). Finite element simulations revealed that reducing substrate thickness further mitigates mechanical stress and strain within the active layer under curvature (Figs. S8 and S9).

Mechanical robustness was further validated under extreme bending conditions: the device retained >95% of its initial output voltage when bent to a radius of 0.15 mm (Fig. 3d), and showed negligible change in dark current or photoresponse under 780 nm illumination following deformation (Fig. 3e, f). Durability testing over 500 and 1000 bending cycles at 0.5 mm radius revealed minimal loss in output performance (Fig. 3g). Although a slight increase in reverse saturation current was observed after 1000 cycles, indicating minor diode degradation (Fig. 3h), the open-circuit voltage remained stable (Fig. 3i). In situ SEM images showed that only slight surface microcracks appeared, while no obvious delamination of the active (Bi,Sb)_2_Se_3_ layer was observed even after 10,000 cycles (Fig. S10). This mechanical and photoelectric robustness is critical for long-term operation in dynamic physiological environments^40^.

### Weak-light conversion efficiency of the OES

**Fig. 4:**
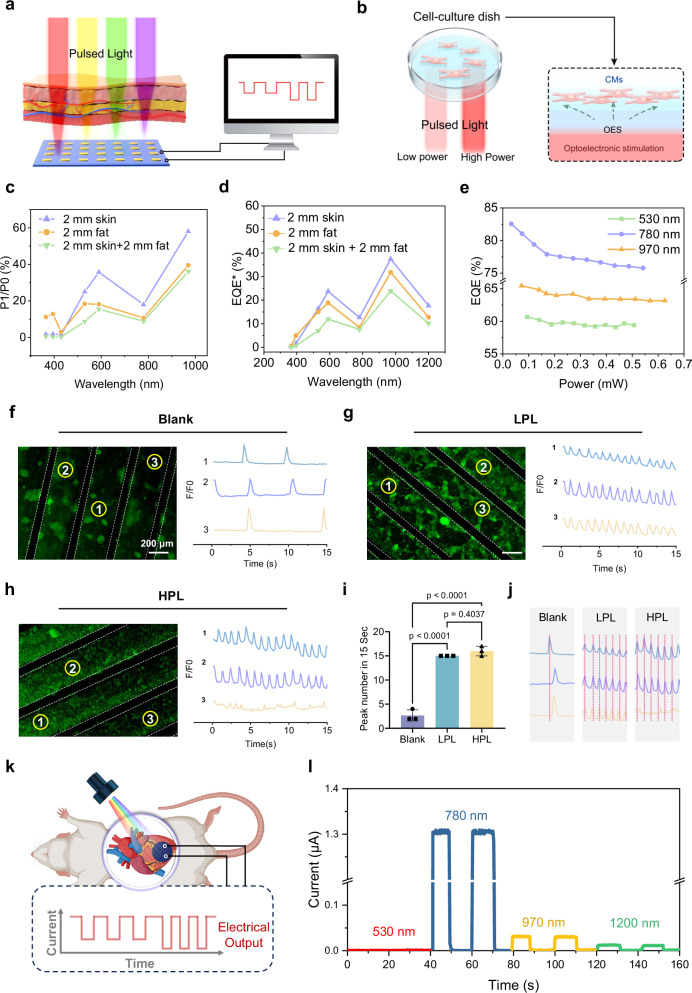
Weak-light conversion efficiency of the optoelectronic stimulator (OES). **a** Schematic illustrating OES activation by incident light of varying wavelengths, generating distinct photocurrent responses. **b** Myocardial cells cultured on the OES are modulated via photostimulation at different light intensities. **c** Comparison of incident (P0) and transmitted (P1) light intensity through different tissue layers (2 mm skin, 2 mm fat, and their combination). **d** EQE* of the device measured under light transmitted through the tissue layers. **e** EQE as a function of wavelength under different incident light intensity. **f–h** Calcium transient imaging of myocardial cells under increasing light intensity, showing frequency-dependent activation by OES even under low-light conditions (LPL, low power light; HPL, high power light). **i** Quantification of Ca^2+^ transient peak occurrences per 5 s from (**f**–**h**), revealing enhanced excitability with increasing light power. **j** Functional contractility assessment of cardiomyocytes across blank, LPL, and HPL conditions, validating light-induced modulation of cardiac activity. **k** Schematic illustration of in vivo implantation of the OES onto the myocardium of SD rats to evaluate wavelength-dependent photoresponsivity. Created in BioRender. ZHAO, R. (2026) https://BioRender.com/y3hfpkw. **l** Cardiac regions were irradiated with lasers of varying wavelengths (530, 780, 970, and 1200 nm), revealing that the peak photocurrent response (~ 1300 nA) was achieved at 780 nm. Data in this figure (**i**) are presented as mean ± S.D.; *n* = 3 independent experiments. Source data are provided as a Source Data file.

To evaluate the photoelectric performance of the OES under physiologically relevant light conditions, the device was placed beneath freshly excised porcine skin and subcutaneous fat to simulate in vivo light attenuation (Fig. 4a). A substantial fraction of incident light successfully traversed these biological tissues and activated the OES, generating robust photocurrents (Fig. 4c, d). Notably, the device maintained high external quantum efficiency (EQE) across a range of weak incident power densities (32.2 μW cm^−2^ to 0.626 mW cm^−2^), with peak EQE values reaching ~80% at 780 nm (Fig. 4e). This high near-infrared sensitivity, within the optical window optimal for deep tissue penetration, underscores the capacity of OES for effective operation under low-intensity, tissue-attenuated illumination.

To examine functional outcomes of optoelectronic stimulation, neonatal rat cardiomyocytes (NRCMs) were seeded on OES substrates micropatterned with 100 μm-wide grooves (Fig. 4b) and subjected to light stimulation at either high (7.62 mW cm^−2^) or low (0.247 mW cm^−2^) power densities (Fig. 4f–h). Fluorescence imaging of Ca^2+^ transients revealed that both illumination regimes induced rhythmic contractions of NRCMs with comparable transient amplitudes (Fig. 4i, j and Supplementary Movie 1), indicating that low-intensity light is sufficient to elicit bioelectric activation via the OES. Under the same experimental conditions, the cells maintained normal morphology, and viability assays confirmed no noticeable phototoxicity under the applied illumination (Fig. S11a), excluding direct light-induced cellular responses. Therefore, the observed calcium signaling modulation originates primarily from the optoelectronic response of the device rather than from direct light–cell interactions. When stimulated at 1 Hz, NRCMs exhibited synchronized contractions across the stimulated region, reflecting enhanced intercellular coupling and functional maturation mediated by photoelectrically triggered calcium signaling^41^.

To further dissect the role of tunable electrical outputs in cardiomyocyte function, we systematically varied electric field (EF) intensity and stimulation frequency (Fig. S12). A specific EF threshold (32 V cm^−1^) markedly enhanced Ca^2+^ transient synchronization and amplitude (Fig. S13 and Supplementary Movie 2), while increasing stimulation frequency selectively elevated oscillation rate without affecting amplitude (Fig. S14 and Supplementary Movie 3). No detectable phototoxic effects or morphological changes were observed under these stimulation conditions, confirming that the functional modulation was not caused by direct light-cell interactions (Fig. S11b, c). Notably, EF stimulation significantly upregulated cardiac troponin T (cTnT) expression, a hallmark of myocyte maturation, and improved sarcomeric organization, contractility, and intercellular junction integrity (Fig. S15).

To identify the optimal light wavelength for weak-light stimulation in vivo, OES devices were implanted into the myocardium of Sprague–Dawley rats (Figs. 4k and S16), and the cardiac region was illuminated with lasers of varying wavelengths (530, 780, 970, and 1200 nm) (Fig. 4l). Among these, 780 nm elicited the strongest photoresponse, generating photocurrents up to ~1300 nA, validating 780 nm as the optimal wavelength for in situ cardiac activation. These findings establish the OES as a highly sensitive, wavelength-tuned bioelectronic interface capable of mediating functional cardiac stimulation under clinically relevant weak-light conditions.

### Biocompatibility and therapeutic efficacy of the OES in myocardial repair

Comprehensive in vitro and in vivo assessments confirmed the cytocompatibility of the OES and its core photoactive component, (Bi,Sb)_2_Se_3_. No detectable cytotoxicity, hemolysis, or inflammatory response was observed (Figs. S17 and 18), indicating low immunogenicity and excellent tissue compatibility. We further immersed the all-inorganic OES in physiological saline for 45 days to simulate the biofluid environment. The OES exhibited negligible morphological changes and less than 5% degradation in photoelectric performance, owing to its intrinsic physicochemical stability (Fig. S19). These properties position the OES as a transdisciplinary platform material with potential for implantable bioelectronic applications.

**Fig. 5:**
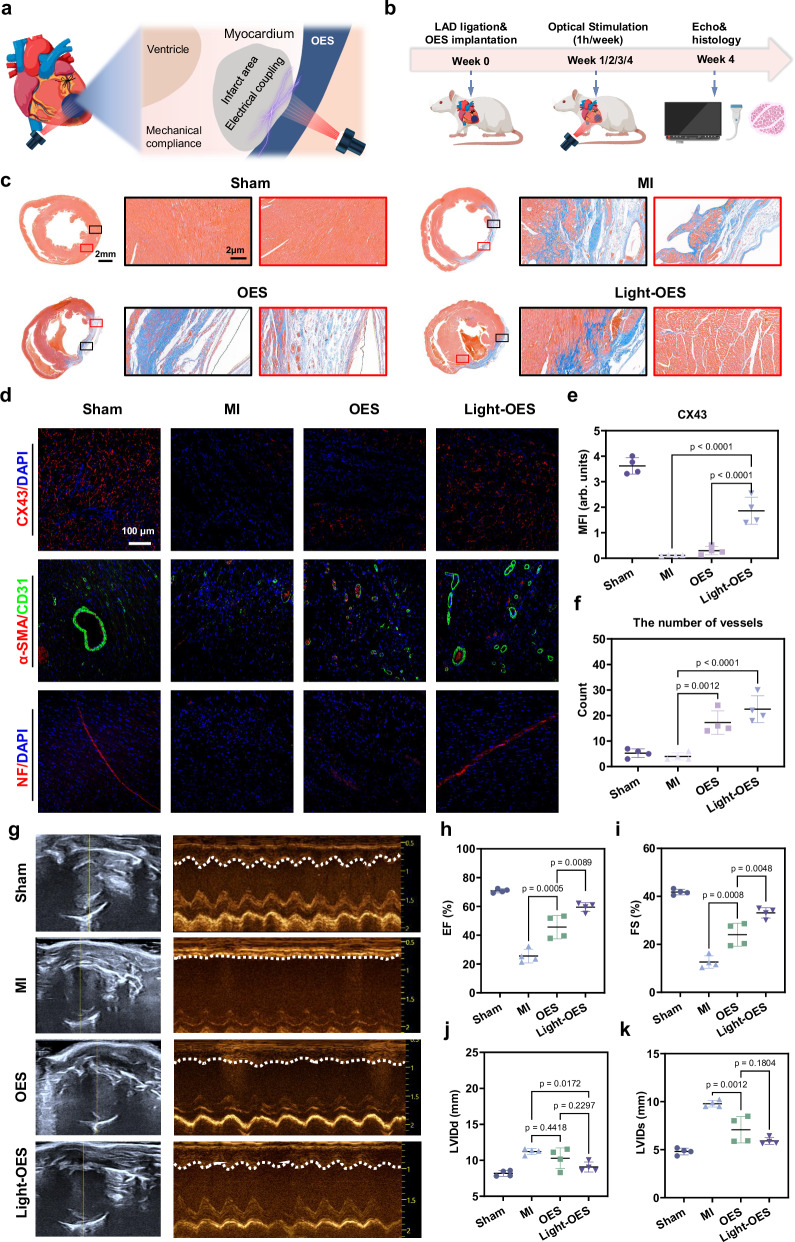
Photoelectric stimulation via OES promotes myocardial remodeling post-infarction. **a** Schematic illustrating implantation of the OES onto infarcted myocardium, designed to enhance mechanical compliance and electrical stimulation. Created in BioRender. ZHAO, R. (2026) https://BioRender.com/y3hfpkw. **b** Myocardial infarction (MI) was induced in rats via left anterior descending (LAD) artery ligation. OES was implanted at the infarct site, and the therapeutic effects of photoelectric stimulation (780 nm) were assessed 4 weeks post-implantation using echocardiography and histology. Created in BioRender. ZHAO, R. (2026) https://BioRender.com/y3hfpkw. **c** Masson’s trichrome staining shows reduced collagen deposition (blue) and increased viable myocardium (red) in the Light-OES group compared to MI and OES controls. **d** Representative immunofluorescence images of infarcted regions stained for Cx43 (electrical coupling), CD31/α-SMA (vascularization), NF (nerve fibers), and DAPI (nuclei). **e** Quantification of Cx43 mean fluorescence intensity (MFI) indicates increased Cx43 expression, suggesting enhanced intercellular electrical connectivity in the Light-OES group. **f** Quantitative analysis of CD31/α-SMA indicates significantly increased microvascular density with Light-OES treatment. **g** Echocardiography performed 4 weeks post-treatment shows improved ventricular wall motion and dimensions (white dashed lines). **h**–**k** Functional parameters of left ventricular performance demonstrate superior cardiac recovery in the Light-OES group. Data in this figure (**e**, **f**, **h**–**k**) are presented as mean ± S.D.; *n* = 4 independent experiments. Source data are provided as a Source Data file.

To evaluate therapeutic efficacy, a rat myocardial infarction (MI) model was established (Fig. 5a and Supplementary Movie 4). Animals were assigned to four groups: Sham, MI, OES (device implanted without light), and Light-OES (device implanted with 780 nm light stimulation at 1 Hz for 1 h per week). During operation, the OES devices with an 8 μm-thick PI substrate and a diameter of 5 mm were placed directly into the infarct zone, with the Au electrode facing the tissue and illuminated from the PI side (back-illumination) to avoid shading by the Au electrode. Light stimulation was administered in the Light-OES group (Fig. 5b and Supplementary Movie 5).

Histological analysis revealed extensive collagen deposition and scar formation in the MI group, whereas the Light-OES group exhibited markedly attenuated fibrosis (Fig. 5c). Connexin 43 (Cx43), a gap junction protein essential for intercellular electrical coupling, was significantly upregulated in the Light-OES group but remained low in MI and OES-only controls (Fig. 5d, e), suggesting restoration of myocardial electrical connectivity. Wheat germ agglutinin (WGA) and cardiac troponin T (cTnT) staining revealed preservation of cardiomyocyte identity and reduced pathological hypertrophy in the Light-OES group (Fig. S20).

Neovascularization was enhanced in both OES-treated groups, as evidenced by CD31/α-SMA co-staining, with significantly greater arteriole formation in the Light-OES group (Fig. 5d, f). Notably, regeneration of nerve fibers was observed exclusively in the Light-OES group, implicating bioelectrical stimulation in promoting coordinated neurovascular remodeling. Cardiac function was evaluated via echocardiography at 4 weeks post-implantation^42^. The MI group exhibited diminished anterior wall motion and severe functional decline, while the OES group showed partial improvement. In contrast, the Light-OES group demonstrated robust ventricular contraction and preserved wall motion (Fig. 5g). Quantitative analysis showed significant recovery in the Light-OES group, with ejection fraction (EF) reaching 59.5% and fractional shortening (FS) at 33.1%, substantially higher than MI or OES-alone groups (Fig. 5h–k). Left ventricular internal diameters at diastole (LVDd) and systole (LVDs) were also reduced, reflecting suppressed ventricular dilation and enhanced contractility^43^.

After 4 weeks of cardiac implantation, the OES was encapsulated by a thin fibrotic tissue layer on the heart surface, while remaining structurally intact after removal of the surrounding tissue (Fig. S21a). The explanted OES showed only minor surface cracks, likely associated with chronic cyclic deformation imposed by cardiac motion, while no obvious large-area delamination of the active layer was observed (Fig. S21c). The photocurrent of the device decreased by 8.9% compared with the pre-implantation value (Fig. S21b, d). This robustness under biological conditions was also observed in the subcutaneous implantation model (Fig. S21e–g). These results demonstrate that the OES retains substantial structural integrity and photoelectric functionality during mid-term in vivo implantation, despite the absence of encapsulation.

Together, these findings highlight the therapeutic potential of the OES system, demonstrating that photoelectric stimulation not only supports structural repair but also facilitates electrophysiological and neurovascular regeneration in infarcted myocardium. While effective in small animal models with thin thoracic walls, light penetration may be limited in thicker human tissues. Future work will focus on optimizing device design and illumination strategies to enable translation of this approach to clinically relevant settings.

### Cardiac pacing performance of the OES in small-animal models

To further validate the capability of the OES for direct cardiac excitation, in vivo pacing experiments were performed in Sprague–Dawley (SD) rats under closed-chest conditions (Fig. S22a). In the closed-chest experiments, the device was implanted on the epicardial surface, and optical stimulation was delivered through the intact chest wall (Fig. S22b). The OES reliably captured cardiac activity, with heart rate increasing from ~240 to ~300 bpm during light stimulation (Fig. S22c–e). No arrhythmias or tissue damage were observed, confirming that the device can effectively convert weak, tissue-attenuated light into electrical signals sufficient to drive cardiac pacing. These results demonstrate that, in addition to supporting myocardial repair, the OES functions as a minimally invasive bioelectronic pacemaker in small animal models, providing a mechanistic link between localized photoelectric stimulation and controlled cardiac excitation.

### Scalable fabrication and large-animal validation of the OES

**Fig. 6:**
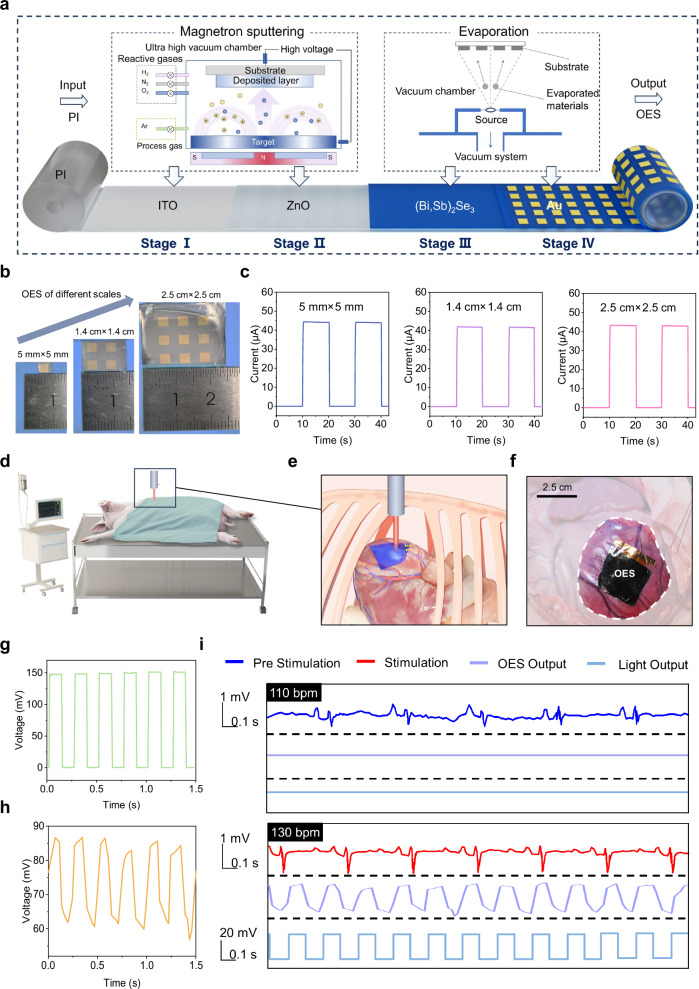
Scalable fabrication and large-animal validation of OES. **a** Schematic diagram of the OES mass production process. Initially, the ITO layer and ZnO layer were deposited using the magnetron sputtering method. Subsequently, the (Bi,Sb)_2_Se_3_ layer and Au electrode were fabricated via thermal evaporation. **b** Photographs of OES of different scales. **c** The *I-T* curves measured from OES of different scales indicate that the scale does not affect the overall performance of the OES. **d** An experimental setup schematically illustrates a mature swine undergoing open-chest cardiac pacing. The key apparatus includes a physiological monitoring station, an anesthetic respiratory support unit, and a computer workstation for regulating illumination parameters. **e** Photograph showing the LED light source emitting infrared light is about 5 cm away from the heart. **f** Device placement on the pig heart designed for multisite optical pacing on left ventricles. The device measures 2.5 × 2.5 cm. **g**, **h** Representative plots demonstrate the *V*_OC_. **g** and the output voltage on the heart surface (**h**) of the device under these test conditions. **i** The output waveforms of OES and light source, ECG signals recorded from the pig during the prestimulation and stimulation periods.

Building on the successful demonstration of cardiac pacing in small-animal models, we next explored the scalability of the OES for large-area device fabrication and its applicability in large-animal studies. The modular design of the OES enables scalable fabrication with tunable geometries, allowing precise adjustment of device dimensions without compromising performance (Fig. 6a, b). Notably, the current output from individual electrode units remained consistent across devices of varying sizes, confirming the stability and scalability of the photoelectric response (Fig. 6c). Consistent photocurrent outputs across multiple electrodes and independently fabricated batches further confirm the good spatial uniformity and reproducible device performance (Fig. S2).

To validate therapeutic potential in a clinically relevant setting, the OES was scaled up to ~2.5 × 2.5 cm and implanted in a porcine model, chosen for its anatomical and electrophysiological similarity to the human heart (Fig. 6d and Supplementary Movie 6). The device was activated using 780 nm near-infrared light (24.7 mW cm^−2^, 4 Hz, 30 min) (Fig. 6e, f and Supplementary Movie 7). In vitro measurements confirmed that the enlarged OES produced a peak open-circuit voltage of 150 mV (Fig. 6g). While in vivo recordings exhibited partial attenuation of the electrical signal, the device maintained rapid and faithful tracking of the input waveform (Fig. 6h). This signal degradation was attributed to low-pass filtering effects intrinsic to the resistance–capacitance (RC) characteristics of myocardial tissue, as supported by an equivalent circuit model (*R* ≈ 2 kΩ, *C* ≈ 50 nF) constructed from established electrophysiological parameters (Fig. S23).

Functional efficacy was assessed in vivo using a multichannel electrophysiological mapping system. Upon photoelectric stimulation, the heart rate increased from 110 to 130 beats per minute (Fig. 6i), demonstrating successful modulation of cardiac rhythm. Electrocardiographic analysis revealed a reduction in P-wave amplitude and a marked enhancement in QRS complex amplitude and sharpness, indicating improved signal conduction and ventricular activation. These results suggest that spatially confined electrical stimuli delivered via the OES enhance intercellular electrical coupling, likely by modulating the local electrophysiological microenvironment.

## Discussion

In summary, we report a wireless, ultra-flexible optoelectric stimulator (OES) based on a one-dimensional (Bi,Sb)_2_Se_3_ semiconductor, capable of efficient weak-light-to-electricity conversion and programmable electrical stimulation. By integrating soft mechanical support with localized photoelectric activation, the OES restored electrical conduction across infarcted regions and improved cardiac function under weak-light stimulation in a rat myocardial infarction model. Importantly, large-animal validation in swine demonstrated that the scalable OES preserves both the stability and tunability of the photoelectric response, reinforcing its translational potential. Together, these findings establish a wireless, ultraflexible, and weak light-driven bioelectronic platform for cardiac repair, while offering broader opportunities for bioelectrical modulation in the treatment of diverse tissue pathologies.

## Methods

### Device fabrication

The PI substrate was custom-fabricated by Wuhan Yimaide New Materials Technology Co., Ltd., with a thickness of 8 μm. Then, the high-infrared-transmittantance tin-doped indium oxide (ITO, Hebei Jiuyue New Materials Technology Co., Ltd.) was deposited on PI substrates by sputtering with a sheet resistance of ~15 Ω □^−1^ and a transmittance of 60% @1300 nm. Subsequently, the ZnO (Hebei Jiuyue New Materials Technology Co., Ltd.) buffer layer was deposited on the PI/ITO. The (Bi,Sb)_2_Se_3_ layer was deposited on top of the ZnO layer by dual-source VTD (Hefei, China, OTF-1200X-II) using Bi_2_Se_3_ and Sb_2_Se_3_ powders as the source. The temperatures of Bi_2_Se_3_ (Jiangxi Ketai Advanced Materials Co., Ltd.) and Sb_2_Se_3_ (Jiangxi Ketai Advanced Materials Co., Ltd.) were set as 600 and 540 °C, respectively, with a heating rate of 20 °C min^−1^. The substrates were placed close to the Sb_2_Se_3_ source with a substrate temperature of 420 °C, a chamber pressure of 2.7 Pa. The deposition time at the target temperatures was 2 min, resulting in a (Bi,Sb)_2_Se_3_ film thickness of approximately 0.8 μm. The gold electrode (80 nm) was deposited on top of the (Bi,Sb)_2_Se_3_ layer by a resistance evaporation system (Beijing Technol Science). Finally, manually peel off the PI from the glass substrate to obtain a complete flexible OES device. A schematic of the whole fabrication process shown in Fig. S1.

### Localized photocurrent mapping

A 780 nm LED (intensity: 24.7 mW cm^−2^) was used as the light source, generating a localized illumination spot with a diameter of approximately 4.5 mm. The LED was positioned approximately 4 cm above the device surface. The device (1.5 × 1.5 cm) consists of a 3 × 3 electrode array. The illumination spot was sequentially positioned to cover different electrode regions across the array. At each position, photocurrent responses from all nine electrodes were simultaneously recorded using a semiconductor parameter analyzer (Agilent B1500A) in a shielded enclosure. The spatial photocurrent distribution was reconstructed based on the recorded response matrix using Origin software.

### EQE test

To measure the external quantum efficiency (EQE) of the optoelectronic stimulation devices, broad-spectral white light from a xenon lamp (300 W, Oriel, 69911, Newport) was dispersed into monochromatic light using a monochromator (Oriel cornerstone^TM^ 130 1/8, Newport). The intensity of monochromatic light was calibrated by a standard silicon photodetector. The photocurrent was recorded using a pre-amplifier and a lock-in amplifier. Then, the EQE spectrum was obtained according to the light intensity and photocurrent. Then, the photoresponsivity (*R*) was calculated based on the equation^44^.

1 $$R={EQE}(q\lambda /{hc})$$

where q is the elementary charge, λ is the wavelength, h is the Planck constant, and c is the velocity of light.

### *I-V* curves test

The Current-Voltage (*I-V*) characteristics were measured from −1 to 1 V under dark conditions and 780 nm LED illumination using a semiconductor device parameter analyzer (Agilent B1500A) in an electrostatically shielded enclosure. Open-circuit voltage (*V*_OC_) was measured as a function of illumination intensity.

### Open-circuit voltage measurements under pulsed illumination

The open-circuit voltage (*V*_OC_) of the OES device was measured using a digital multimeter, with the positive and negative leads connected to the indium tin oxide (ITO) common electrode and the Au electrode, respectively. Pulsed illumination was provided by a calibrated light source at 24.7 mW cm^−2^ with a 50% duty cycle. The pulse frequency was varied (1, 2, and 3 Hz), and the corresponding *V*_OC_ of the OES was recorded under each condition.

### Laser scribing and module fabrication

(Bi,Sb)_2_Se_3_ mini-modules were fabricated using a standard three-step laser scribing process, following a previously reported procedure with minor modifications^45^. ITO substrates (5 × 5 cm) were first patterned by a 1064 nm femtosecond laser (KW-20W-cx, Wuhan, China) to form the P1 lines (10 W, 900 mm s^−1^), which served as the bottom electrode. A ZnO buffer layer followed by an (Bi,Sb)_2_Se_3_ absorber layer was subsequently deposited onto the patterned substrates. The P2 scribing (10 W, 1300 mm s^−1^) was then applied to locally remove the ZnO/(Bi,Sb)_2_Se_3_ layers, exposing the underlying ITO and enabling interconnection between adjacent sub-cells. After deposition of the Au electrode, P3 scribing (10 W, 1500 mm s^−1^) was performed to remove the Au electrode and electrically isolate neighboring sub-cells. The final (Bi,Sb)_2_Se_3_ mini-modules were formed by monolithic series interconnection of the individual sub-cells. Schematic of the fabrication workflow and photograph of the micro-modules are shown in Figs. S6 and S7.

### FEA modeling

Finite element models were built in COMSOL Multiphysics 6.1 to evaluate the mechanical responses of the flexible optoelectronic devices. A multilayer structure consistent with the fabricated device geometry was constructed. An in-plane compressive load was applied to the side of the film, and the loaded surface was constrained to allow only in-plane displacement. The strain at the center of the (Bi,Sb)_2_Se_3_ layer was extracted, and the corresponding stress was obtained from the same simulation results. A hybrid mesh combining quadrilateral and tetrahedral elements was used for computation. Finite element parameters used in the model, including thickness, Young’s modulus, and Poisson’s ratio for each functional layer are detailed in Table S1.

### Mechanical bending test

Mechanical bending was applied using a motorized tensile tester (CTM6050). The OES devices were pre-compressed and then subjected to repeated stretch-compression cycles. The pre-bending displacement was adjusted according to different bending radii, with larger compression distances used to achieve smaller radii and higher strain levels. After cyclic bending for the specified number of cycles, the devices were subsequently characterized for optoelectronic performance and morphological changes.

### In vitro electric field stimulation using interdigitated electrodes

For in vitro stimulation, the positive and negative terminals of the mini-modules were connected to an interdigitated electrode (IDE) array composed of parallel gold fingers (width: 100 μm; spacing: 300 μm). The electric field strength was approximated as E = V/d, where V is the applied potential difference and d is the inter-finger spacing. Field magnitude was tuned by adjusting the output voltage and selecting different electrode contact pairs. Stimulation frequency was controlled by illuminating the mini-modules with pulsed light at programmable frequencies, generating electrical outputs at corresponding frequencies. Neonatal rat cardiomyocytes (NRCMs) were seeded on the IDE surface and stimulated with defined field strengths and frequencies.

### Isolation and culture of NRCMs

Sprague–Dawley rats of 1-day-old from Hubei Provincial Center for Disease Control were used. Briefly, in a sterile environment, the chest was promptly opened, and the fresh heart was taken out. After the epicardium was peeled off, the remaining myocardial tissue was minced and digested at 40 °C with digestive fluid (containing 0.25% trypsin and type II collagen) to convert the myocardial tissue into single cardiomyocytes. After 3 h of differential adhesion, non-adherent NRCMs (> 90%) were collected and subsequently seeded onto interdigital electrode. Cells were cultured in Dulbecco’s Modified Eagle Medium (DMEM, Gibco), supplemented with 10% (v/v) fetal bovine serum (FBS, HyClone) and 1% (v/v) penicillin/streptomycin (PS, Gibco) in a humidified 5% CO_2_ incubator at 37 °C.

### In vitro biocompatibility assessment

H9C2(2-1) cells were procured from HaiXing (Suzhou, China). The cells were cultivated with the leach liquor of flexible (Bi,Sb)_2_Se_3_. After 1, 2, and 3 days of incubation, the cells were washed with phosphate buffer saline (PBS) three times.

CCK-8: 10 μL of Cell Counting Kit-8 (CCK-8; Biosharp, China) was added. After 1 h of incubation, the cell viability was determined using a microplate reader based on the optical density at 450 nm.

Live/Dead staining: The cells were stained with calcein-AM/PI (YEASEN, China) in accordance with the manufacturer’s instructions. The stained cells were observed using an inverted fluorescence microscope. (Nikon, ECLIPSE Ts2, Japan).

β-actin/DAPI (4’,6-diamidino-2-phenylindole) staining (Sigma-Aldrich, USA): The cells were fixed with 4% paraformaldehyde, treated with 0.1% Triton X-100 and stained with β-actin and DAPI as per the manufacturer’s instructions. The stained cells were observed using inverted fluorescence microscope. (Nikon, ECLIPSE Ts2, Japan).

For the co-culture experiment, Sb_2_Se_3_ solutions at varying concentrations were prepared using high-glucose medium (1 μg mL^−1^, 10 μg mL^−1^, 100 μg mL^−1^, 1 mg mL^−1^). These solutions were subsequently co-cultured with H9C2 cells and NRCMs. All subsequent experiments were conducted in accordance with the previously outlined procedural steps.

### In vivo biosafety assessment

In vivo biosafety assessment was conducted on 6 male SD rats (8 weeks old) obtained from the Hubei Provincial Center for Disease Control and Prevention. The SD rats were housed in a standard environment with a temperature ranging from 24 to 26 °C, a humidity of 70–75%, a 12-h light–dark cycle, and typical laboratory meals. All the experimental protocols in this study were approved by the Committee of Huazhong University of Science and Technology. A flexible (Bi,Sb)_2_Se_3_ device with a diameter of 5 mm was subcutaneously implanted beneath the dorsal skin of the rats for 30 days. Subsequently, major organs (heart, liver, spleen, lung, and kidney) as well as the skin directly above the flexible (Bi,Sb)_2_Se_3_ implant site were harvested for histological examination.

### Calcium transient imaging

After a 7-day culture, the NRCMs were stained with Fluo-4 AM (Thermo Fisher, F14201) to observe the Ca^2+^ activity in accordance with the production instruction. Briefly, the samples were washed with PBS and then incubated in the Fluo-4 AM working solution at 37 °C with 5% (v/v) CO_2_ for 15–20 min. Finally, the samples were washed using PBS and replaced with the normal medium. The calcium activity was recorded under a fluorescence microscope (NIS-Elements D Software). The video data were analyzed by ImageJ software and Origin 2021.

### Immunofluorescence staining and confocal microscopy

After 7 days of culture, the NRCMs were fixed in 4% formaldehyde for 30 min at room temperature. Then, cells were permeabilized with 0.5% Triton X-100 at room temperature for 20 min, and washed three times with PBS, 3 min/time. Blocked with 5% BSA at room temperature for 45 min. Incubated with cTnT (Abcam, ab209813, 1:500) overnight at 4 °C. Followed incubated with Goat Anti-Rabbit IgG, TGFBR3-Specific Polyclonal antibody (Proteintech, 20000-1-AP, 1:300) for 45 min at room temperature. Nuclei were counterstained with 4’,6-diamidino-2-phenylindole (DAPI, Sigma Aldrich).

### Establishment of rat MI model and transplantation of OES

The myocardial infarction (MI) model was established in 200 ± 20 g SD male rats (provided by Hubei Provincial Center for Disease Control and Prevention). They were categorized into the sham operation group, MI group, OES group, and Light-OES group.

Briefly, rats were anesthetized intraperitoneally with 1% pentobarbital sodium (0.8 mL/200 g), followed by endotracheal intubation on a small animal ventilator. Subsequently, the third and fourth ribs on the left side of the rib cage were opened to expose the beating heart. Rats in the sham operation group underwent thoracotomy, while the other rats underwent ligation of the left anterior descending branch after thoracotomy. OES was implanted on the surface of the infarcted myocardium, and the edge of the patch was sutured to the epicardial membrane of the infarcted myocardium using 7-0 polypropylene sutures. The chest cavity was closed after the operation.

### Histological, immunohistochemical, and immunofluorescence analysis

Four weeks after surgery, all the animals were euthanized, the isolated hearts were PFA-fixed, paraffin-embedded, and then sectioned at a thickness of 5 μm. For immunofluorescence staining, the sections were incubated with Cx43 (Abcam, ab11370, 1:500), WGA (Servicebio, G1730, 1:200), NF (Proteintech, 21471-1-ap, 1:200), CD31 (Abcam, ab182981, 1:500), α-SMA (Proteintech, 67735-1-ap, 1:500), and cTnT (Abcam, ab209813, 1:500) overnight at 4 °C followed by incubation with the secondary antibodies for 1 h. Afterward, the paraffin sections were stained with DAPI, ImageJ software was used to quantify their relative expression.

### Echocardiography test

Four weeks following OES transplantation, the left ventricular (LV) function of the rats were assessed using an echocardiography system (VINNO6 LAB). M-mode and B-mode imaging, which reflect the morphology and contractile activity of the LV anterior wall, were utilized. Functional parameters such as fractional shortening (FS) and ejection fraction (EF) were calculated based on three consecutive cardiac cycles.

### In vivo cardiac pacing in SD rats

SD rats received a tail vein injection of verapamil hydrochloride saline solution (3 mg mL^−1^, 0.6–0.8 mL) to reduce the heart rate to approximately one-half of the baseline value. All subsequent procedures were completed within 20 min after drug administration. A thoracotomy was performed to expose the heart, and the flexible OES device was gently bent and inserted into the space between the pericardium and myocardium. The device was stably maintained in position by conformal contact with surrounding cardiac tissues, without the use of sutures or adhesive materials. The OES functions as a self-powered photovoltage source, while the stimulation field is established in the tissue by a lateral potential difference between the Au and ITO electrodes, rather than within the multilayer stack itself. After device placement, the chest cavity was closed.

External LED illumination was applied to activate the OES while electrocardiogram (ECG) signals were recorded simultaneously. The illumination parameters were 780 nm wavelength, 24.7 mW cm^−2^ optical intensity, 5 Hz flicker frequency, and a square wave with a 50% duty cycle.

### In vivo stimulation of the porcine heart

A deep level of anesthesia was induced in an adult pig using inhalation anesthesia. Concurrently, a detection system was employed to continuously monitor the animal’s electrocardiogram (ECG), heart rate (HR), end-tidal CO_2_ (EtCO_2_), blood oxygen saturation (SpO_2_), respiratory rate (RR), blood pressure (BP), and body temperature in real time. During the procedure, the pig was positioned in a supine posture, and a median sternotomy was performed to expose the heart. Following the opening of the pericardium, the optoelectronic stimulator (OES) was placed on the surface of the left ventricle, ensuring its natural adherence. Stable contact was achieved by conformal contact with the cardiac tissue. The overall dimensions of the OES are approximately 2.5 × 2.5 cm, with electrodes arranged in an array at 0.5 cm intervals. Voltage data from the OES were recorded both on the cardiac surface and in the open circuit using the DMM6500 (Keithley). LED light sources were used to illuminate the surface of the OES, thereby activating photoelectric stimulation. The illumination parameters were as follows: emission wavelength of 780 nm, optical intensity of 24.7 mW cm^−2^, flicker frequency of 4 Hz, and signal form of a square wave with a 50% duty cycle. ECG data were acquired using the TOP-2001 series multi-channel electrophysiological system before and during stimulation to evaluate cardiac function.

### Statistics and reproducibility

All data were presented as means ± standard deviation. Statistical analyses were conducted using GraphPad Prism version 10.0 and Origin 2021 software. For two-group comparisons, Student’s *t*-test was employed, while one-way analysis of variance (ANOVA) followed by the post hoc Bonferroni test was used to assess differences among multiple groups. *p* < 0.05 was considered statistically significant. All experiments were independently repeated at least three times with similar results. Representative SEM, HRTEM, fluorescence, histological, and immunofluorescence images shown in the manuscript were selected from these independent experiments. Male SD rats and male pigs were used in this study. Although sex was considered as a biological variable, only male animals were included to minimize the potential confounding effects of estrogen on cardiac repair after myocardial infarction.

### Ethical

All animal experiments were carried out following protocols approved by the relevant ethical committees. Experiments involving Sprague–Dawley rats were approved by the Institutional Animal Care and Use Committee of Hubei Provincial Center for Disease Control and Prevention (No. 202420126). Experiments involving pigs were approved by the Animal Ethics Committee of Yinshe (Shanghai) Medical Technology Co., Ltd. (SHYS-No.2025049).

### Reporting summary

Further information on research design is available in the Nature Portfolio Reporting Summary linked to this article.

## Supplementary information

Supplementary information

Description of Additional Supplementary Files

Supplementary Movie 1

Supplementary Movie 2

Supplementary Movie 3

Supplementary Movie 4

Supplementary Movie 5

Supplementary Movie 6

Supplementary Movie 7

Reporting Summary

Transparent Peer Review file

## Source data

Source Data

## Data Availability

All data supporting the findings of this study are available within the article and its supplementary files. Any additional requests for information can be directed to and will be fulfilled by the corresponding authors. Source data are provided with this paper.
