# Supplementary material for: Ultra-flexible optoelectronic stimulator converts tissue-attenuated weak light into electrical signals for cardiac remodeling
Source: Nat Commun. 2026 Jul 13;17:8622. doi: 10.1038/s41467-026-75495-7 (PMC13486475; doi:10.1038/s41467-026-75495-7)
Supplement: Supplementary file 1 [file 41467_2026_75495_MOESM10_ESM.pdf]

nature portfolio

Corresponding author(s): Chuanbin Mao; Jianglin Wang; Kanghua Li

Last updated by author(s): May 8, 2026

Reporting Summary

Nature Portfolio wishes to improve the reproducibility of the work that we publish. This form provides structure for consistency and transparency in reporting. For further information on Nature Portfolio policies, see our Editorial Policies and the Editorial Policy Checklist.

Statistics

For all statistical analyses, confirm that the following items are present in the figure legend, table legend, main text, or Methods section.

n/a	Confirmed	
☐	☒	The exact sample size (*n*) for each experimental group/condition, given as a discrete number and unit of measurement
☐	☒	A statement on whether measurements were taken from distinct samples or whether the same sample was measured repeatedly
☐	☒	The statistical test(s) used AND whether they are one- or two-sided<br>*Only common tests should be described solely by name; describe more complex techniques in the Methods section.*
☒	☐	A description of all covariates tested
☐	☒	A description of any assumptions or corrections, such as tests of normality and adjustment for multiple comparisons
☐	☒	A full description of the statistical parameters including central tendency (e.g. means) or other basic estimates (e.g. regression coefficient) AND variation (e.g. standard deviation) or associated estimates of uncertainty (e.g. confidence intervals)
☐	☒	For null hypothesis testing, the test statistic (e.g. *F*, *t*, *r*) with confidence intervals, effect sizes, degrees of freedom and *P* value noted<br>*Give P values as exact values whenever suitable.*
☒	☐	For Bayesian analysis, information on the choice of priors and Markov chain Monte Carlo settings
☒	☐	For hierarchical and complex designs, identification of the appropriate level for tests and full reporting of outcomes
☒	☐	Estimates of effect sizes (e.g. Cohen's *d*, Pearson's *r*), indicating how they were calculated

Our web collection on statistics for biologists contains articles on many of the points above.

Software and code

Policy information about availability of computer code

Data collection	FV31S-SW, Ultrasound imaging system, IO control software, BL-420N biological signal acquisition and analysis system, Multi-lead ECG analysis system, Origin 2021 and GraphPad prism10.0.
Data analysis	Calcium imaging data were analyzed using Image J. Fluorescent images were analyzed by FV31S-DT.

For manuscripts utilizing custom algorithms or software that are central to the research but not yet described in published literature, software must be made available to editors and reviewers. We strongly encourage code deposition in a community repository (e.g. GitHub). See the Nature Portfolio guidelines for submitting code & software for further information.

Data

Policy information about availability of data

All manuscripts must include a data availability statement. This statement should provide the following information, where applicable:

- Accession codes, unique identifiers, or web links for publicly available datasets
- A description of any restrictions on data availability
- For clinical datasets or third party data, please ensure that the statement adheres to our policy

All data needed to evaluate the conclusions are present in the paper and/or the Supplementary Materials.

Research involving human participants, their data, or biological material

Policy information about studies with human participants or human data. See also policy information about sex, gender (identity/presentation), and sexual orientation and race, ethnicity and racism.

Reporting on sex and gender	*Use the terms sex (biological attribute) and gender (shaped by social and cultural circumstances) carefully in order to avoid confusing both terms. Indicate if findings apply to only one sex or gender; describe whether sex and gender were considered in study design; whether sex and/or gender was determined based on self-reporting or assigned and methods used. Provide in the source data disaggregated sex and gender data, where this information has been collected, and if consent has been obtained for sharing of individual-level data; provide overall numbers in this Reporting Summary. Please state if this information has not been collected. Report sex- and gender-based analyses where performed, justify reasons for lack of sex- and gender-based analysis.*
Reporting on race, ethnicity, or other socially relevant groupings	*Please specify the socially constructed or socially relevant categorization variable(s) used in your manuscript and explain why they were used. Please note that such variables should not be used as proxies for other socially constructed/relevant variables (for example, race or ethnicity should not be used as a proxy for socioeconomic status). Provide clear definitions of the relevant terms used, how they were provided (by the participants/respondents, the researchers, or third parties), and the method(s) used to classify people into the different categories (e.g. self-report, census or administrative data, social media data, etc.) Please provide details about how you controlled for confounding variables in your analyses.*
Population characteristics	*Describe the covariate-relevant population characteristics of the human research participants (e.g. age, genotypic information, past and current diagnosis and treatment categories). If you filled out the behavioural & social sciences study design questions and have nothing to add here, write "See above."*
Recruitment	*Describe how participants were recruited. Outline any potential self-selection bias or other biases that may be present and how these are likely to impact results.*
Ethics oversight	*Identify the organization(s) that approved the study protocol.*

Note that full information on the approval of the study protocol must also be provided in the manuscript.

Field-specific reporting

Please select the one below that is the best fit for your research. If you are not sure, read the appropriate sections before making your selection.

☒ Life sciences ☐ Behavioural & social sciences ☐ Ecological, evolutionary & environmental sciences

For a reference copy of the document with all sections, see nature.com/documents/nr-reporting-summary-flat.pdf

Life sciences study design

All studies must disclose on these points even when the disclosure is negative.

Sample size	The minimum n≥3 biological replicates were used for all experiments. n values was reported in figure legends and supporting information.
Data exclusions	No data were excluded from analysis.
Replication	All experimental data were reproduced and all preparation, processing and characterization of materials were performed a minimum of three times. All in vivo experiments were performed at least three times.
Randomization	Samples were randomly allocated to each group.
Blinding	Throughout the experiments, personnel who contributed to data acquistion remained unaware of group identities while analyses were performed.

Reporting for specific materials, systems and methods

We require information from authors about some types of materials, experimental systems and methods used in many studies. Here, indicate whether each material, system or method listed is relevant to your study. If you are not sure if a list item applies to your research, read the appropriate section before selecting a response.

Materials & experimental systems

n/a	Involved in the study	
☐	☒	Antibodies
☐	☒	Eukaryotic cell lines
☒	☐	Palaeontology and archaeology
☐	☒	Animals and other organisms
☒	☐	Clinical data
☒	☐	Dual use research of concern
☒	☐	Plants

Methods

n/a	Involved in the study	
☒	☐	ChIP-seq
☒	☐	Flow cytometry
☒	☐	MRI-based neuroimaging

Antibodies

Antibodies used	cTnT(Rabbit,abcam,ab209813,1:500);CX43(Rabbit,abcam,ab11370,1:500);CD31(Mouse,abcam,ab182981,1:500);iNOS(Mouse,abcam, ab49999,1:400); α-SMA(Mouse,Proteintech,67735-1-ap, 1:500); TNF-α(Rabbit,Proteintech,17590-1-ap,1:200); IL-1β (Rabbit, Proteintech, 26048-1-ap, 1:200);NF(Rabbit, Proteintech, 21471-1-ap, 1:200)
Validation	Antibodies were validated according to manufacture's description.

Eukaryotic cell lines

Policy information about cell lines and Sex and Gender in Research

Cell line source(s)	Neonatal rat cardiomyocytes (NRCMs) were isolated in our lab according to Wang, L, et al. Nat. Biomed. Eng. 5, 1157 (2021). H9C2 cells were purchased from Haixing Biosciences (TCR-C607).
Authentication	No cell line authentication was performed. However, at least three biological replicates were included for each experiment to ensure reproducibility.
Mycoplasma contamination	We confirm that all cell lines were negative for mycoplasmas contamination.
Commonly misidentified lines (See ICLAC register)	There were no commonly misidentified lines for our study.

Animals and other research organisms

Policy information about studies involving animals; ARRIVE guidelines recommended for reporting animal research, and Sex and Gender in Research

Laboratory animals	1–3-days old Sprague-Dawley rats were used for the isolation of cardiomyocytes. Adult male Sprague-Dawley rats (7 weeks old, 200–220 g) were used for myocardial infarction (MI) modeling, OES transplantation, and pacing experiments. Male Large White × Landrace crossbred pigs(50 kg) were used for pacing experiments.
Wild animals	No wild animals were used in this study.
Reporting on sex	The results apply to both females and males.
Field-collected samples	This study did not involve samples from the field.
Ethics oversight	All animal experiments were carried out in accordance with the Regulations for the Administration of Affairs Concerning Experimental Animals (China). Experiments involving Sprague-Dawley rats were approved by the Institutional Animal Care and Use Committee of Hubei Provincial Center for Disease Control and Prevention (No. 202420126). Experiments involving pigs were approved by the Animal Ethics Committee of Yinshe (Shanghai) Medical Technology Co., Ltd. (SHYS-No.2025049).

Note that full information on the approval of the study protocol must also be provided in the manuscript.

Plants

Seed stocks

Report on the source of all seed stocks or other plant material used. If applicable, state the seed stock centre and catalogue number. If plant specimens were collected from the field, describe the collection location, date and sampling procedures.

Novel plant genotypes

Describe the methods by which all novel plant genotypes were produced. This includes those generated by transgenic approaches, gene editing, chemical/radiation-based mutagenesis and hybridization. For transgenic lines, describe the transformation method, the number of independent lines analyzed and the generation upon which experiments were performed. For gene-edited lines, describe the editor used, the endogenous sequence targeted for editing, the targeting guide RNA sequence (if applicable) and how the editor was applied.

Authentication

Describe any authentication procedures for each seed stock used or novel genotype generated. Describe any experiments used to assess the effect of a mutation and, where applicable, how potential secondary effects (e.g. second site T-DNA insertions, mosiacism, off-target gene editing) were examined.
