# Supplementary material for: Ultra-flexible optoelectronic stimulator converts tissue-attenuated weak light into electrical signals for cardiac remodeling
Source: Nat Commun. 2026 Jul 13;17:8622. doi: 10.1038/s41467-026-75495-7 (PMC13486475; doi:10.1038/s41467-026-75495-7)
Supplement: Supplementary file 2 [file 41467_2026_75495_MOESM11_ESM.pdf]

nature portfolio

Peer Review File

Ultra-flexible Optoelectronic Stimulator Converts Tissue-Attenuated Weak Light into Electrical Signals for Cardiac Remodeling

Corresponding Author: Professor Chuanbin Mao

This file contains all reviewer reports in order by version, followed by all author rebuttals in order by version.

Version 0:

Reviewer comments:

Reviewer #1

(Remarks to the Author)
Review for the paper "Ultra-flexible Optoelectronic Stimulator Converts Tissue-Attenuated Weak Light into Electrical Signals for Cardiac Remodeling" (also attached as a doc file)

Overall comments:
In this article, the authors present an elegant design for a flexible device proposed to perform cardiac stimulation through optoelectronic conversion. For this purpose, the authors propose using a p-n junction formed between ZnO and $(Bi,Sb)_2Se_3$ which is supported by the flexible substrate for the increased biocompatibility. The device relies on efficient conversion of infrared light, 780nm, into open circuit voltage by the pn-junction which is then activates cardiac cells. The device is well characterized in dry conditions, followed by in vitro and animal testing. Overall the device is showing very interesting behavior. As a result, this article will certainly be interesting to the wide audience of Nature Communications. However there are a few major corrections that have to be addressed to reach the necessary quality level of the journal. At the moment, there are missing parts of fabrication and measurement details which do not provide sufficient information for reproducibility purposes. There also a few questions regarding the device performance in biological environments, such as (i) device stability as the active layer is in direct contact with the tissues and liquids and (ii) phototoxicity of the device and induced heating effects. Hence, my recommendation is to accept the manuscript with major corrections.

Major corrections:
1. Fig. 1 schematic suggests that the material fully opens up upon bending, if this is the case such materials would be expected to have quite low stability in the biological environment. While authors refer to the material as vdW layers, there is no either references provided or TEM measurements provided to confirm the exact structure of the material. Additionally, there is very little SEM images provided to characterize the material quality, there is only a small inset in fig 2d. Please provide SEM images at lower magnification to demonstrate the film quality over large scale for the supplementary. Furthermore, the conclusions that there are pertrusions going all the way down in the film are not very well supported. The side view SEM of the film shows that it is polycrystalline certainly. Perhaps authors have more data to support this or more references.
2. In the Figure 2b, the authors provide the current mapping of the device. In general, it is indeed a great way to understand the responsivity of a photodiode. However, the authors do not provide an explanation of where the electrodes are connected or what the maps are. There is no scale bar, there is no explanation how far off the gold pad they are illuminating, there is also no note about the intensity of laser source they are using.
3. Figure 2c shows a typical diode performance curves, however this is very unclear if those are measured for this specific device. If they are measured for this particular device, there should be explicit numbers for current and for voltage, furthermore, there is no reference or explanation what abbreviations provided in the graph mean, which can make it hard to follow the paper for people coming from a broad range of backgrounds.
4. Figure 2 e-h, it is unclear how the devices have been characterized. Given, that the device mapping in 2b are highly non-uniform with a current varying at least 6 times across the device, it is not clear how those performance metrics have been measured then. Was the position of the 780nm diode illumination positioned at the point of maximum photocurrent? If so, this should be clearly stated. Given that a 780nm diode is used for the measurements is focused using a lens, what is the illumination spot is reached based on FWHM?

5. The authors show the results for a range of light illuminations, but what is a safe range for tissue illumination and when the heating/damage occurs? "The output voltage was tunable between 0.02 V and 0.22 V by varying the incident intensity from 0.05 to 24.7 mW cm-2"
6. There is almost no explanation how the minimodules are fabricated. The general explanation of the unit fabrication is also missing some significant information which can result in very low reproducibility of these results (see more comments on this below). The only minimodule fabrication details are provided briefly in the supplementary and are not sufficient for reproducibility. It is quite important to see the sem images of those minimodules as well. Provided info "For the mini-module fabrication, the three-step laser etching technique was employed, including the P1 etching to cut off the ITO bottom electrode, P2 etching to remove the ZnO and (Bi,Sb)2Se3 layer, and P3 etching to cut off the Au top electrode." How all of these etching steps are done without damaging polyimide is unclear as well.
7. Figure 2j what is the light intensity used? Figure 2k barely possible to read the light intensity scale, perhaps use 1, 10, 100 mW/cm2 but again what are the biological limitations in terms of heating/tissue damage?
8. "Fabricated on an optimized polyimide (PI) substrate" there are no details about the thickness of polyimide used nor the fabrication steps used to make it.
9. How is this measured or estimated? There are no details in the text: "Upon bending, the $(Bi,Sb)_2Se_3$ layer experienced an in-plane strain of ~0.083%, corresponding to a stress of ~1.45×10^6 Pa, significantly lower than that in conventional three-dimensional semiconductors such as ZnO and ITO (Fig. 3b)." In figure 3c, there are results of Comsol calculations for 8um PI substrate, is this the thickness used in the paper? There is no mentioning of the actual thickness of the PI layer used for these devices.
10. Re the mechanical resilience, it is quite important to check the active material properties post-bending with SEM, those would show if there was any material delamination or significant crack propagation. "The mechanical resilience of the OES arises from the intrinsic van der Waals bonding and benign grain boundaries within the one-dimensional crystal framework of $(Bi,Sb)_2Se_3$, which confer exceptional tolerance to deformation and structural imperfections." Minor: Why there is an intrinsic vdW bonding, please provide references?
11. The mechanical robustness curves are quite impressive, however, similarly to the comment number 3 above, it is unclear how this is measure and how the device is connected, based on the photo of the devices, those are not mini-modules from the authors explanations, is this correct? As from the schematics the minimodules appear to have continuous gold electrode.
12. Fig 4 c-e, same comments as N3 and N10, where the illumination is located and how is it calculated.
13. Beautiful stimulation results for the cardiac cells observed, minor questions regarding the duration of the culture on the device prior the measurements? The major questions, are the cells survive after such stimulation? i e phototoxicity assessment? Major question, how do cell correspond to the illumination at these powers without the device present, i e is there any direct cell heating at those illumination intentisities?
14. How does the device look like post implantation? Was there any SEM assessment of the active layers degradation? The active material is in direct contact with the biological tissues, surely this will lead to a significant degradation and delamination, as there is no encapsulation used.
Minor corrections:
1. In abstract, the following sentence needs to be paraphrased as the meaning is somewhat unclear, especially for a broad audience of the journal: "clinical translation is hindered by the rigidity and weak-light inefficiency of conventional silicon-based devices" Please specify more explicitly what is the issue of weak-light inefficiency. Perhaps including another sentence on requirements for illumination conditions would be helpful too.
2. For the fig 2b, the scale bar can be in microamperes, hence better readability and no need to x10^-4.
3. Fig 3c has quite important device information regarding thicknesses of the layers, but those are very challenging to read, similarly to the scale bars.
4. Figure 4, for the sensitive audience, please move the actual photo of a mouse and a setup to the supplementary.
5. In the supplementary section, the fabrication details are not fully provided which can reduce the reproducibility of these devices:
a. Please include a reference here: "The PI substrates with high-temperature resistance and high transmittance were fabricated similarly to the previous report [XX]." How thick is the PI layer?
a. "Subsequently, the ZnO buffer layer was deposited on the PI/ITO." Is this sputtering and what thicknes? What thickness is the ITO layer?
b. If the method for (Bi,Sb)2Se3 layer fabrication has been already reported by the team, please include references on your past papers with this material. " The (Bi,Sb)2Se3 layer…dual-source VTD using Bi2Se3 and Sb2Se3 powders as the source [XXX]." Important details might be missing for the reproducibility purposes, such as vacuum value during the deposition process and temperature of the substrate (was it water cooled?). From analysis of the references for the main article, it was not possible to locate previous work on using this material system.
c. One of the concerns regarding the fabrication techniques on the polyimide substrates is the temperature budget of the flexible substrate and induced strain. In our fabrication with polyimide substrates, we've seen the challenges of the strain build-up in the substrates which can lead to the material unintended deformation. Hence, it would be very valuable if the team can comment on those.
d. This sentence is quite confusing: "The temperature of Bi2Se3 and Sb2Se3 was set as 600 °C and 540 °C, respectively, with a heating rate of 20 °C min-1 and "deposition?" duration of 2 min to obtain the target thickness of XXX."
e. This information might not be sufficient yet again for reproducibility of the fabrication process, it might be beneficial to provide more specific information and the importance of the distance variation for the film quality. "The substrates were placed close to the Sb2Se3 source."
f. Is it only gold deposited without any adhesion layer (Ti or Cr)? "The gold electrode was deposited on top of the (Bi,Sb)2Se3 layer by a resistance evaporation system (Beijing Technol Science)."
g. Based on the Figure S1 there is also exfoliation and peeling off steps. But those are not describe in the fabrication section.

Reviewer #2

(Remarks to the Author)
The manuscript "Ultra-flexible optoelectronic stimulator converts tissue-attenuated weak light into electrical signals for cardiac remodeling" by Zhao and colleagues describes the development and validation of a light-driven, flexible stimulator for cardiac therapy. The authors engineer a high-sensitivity photodetector based on bismuth-doped antimony selenide, p-$(Bi,Sb)_2Se_3$. This material choice is intended to facilitate the conversion of low-intensity light into electrical impulses delivered to the heart tissue. The study provides comprehensive characterizations, including optoelectronic performance and mechanical flexibility. Furthermore, the authors present both in vitro and in vivo validations to demonstrate the utility in stimulating cardiac cells and tissue. This work represents a promising step toward the development of light-coupled therapies for myocardial infarction regeneration and the realization of wireless, optical pacemakers. While the results are compelling and address a significant need for less invasive cardiac interfaces, the manuscript currently suffers from a lack of detail in the methodology. These omissions hinder a complete interpretation of the data and the reproducibility of the findings. Specific concerns regarding the experimental design and data reporting are detailed below.

Major concerns:
1) Device fabrication methods are not sufficiently described, and authors leave a lot of room for interpretation to the reader. Authors must comprehensively describe all the device fabrication methods including the scalable fabrication. Below are some examples.
1a. According to the methods in the SI, the device is fabricated as stacked multilayer, but then the devices in most figures are represented as isolated island. This is confusing.
1b. The authors indicate they fabricate mini-modules, but the method is not clear at all. What type of PVD where use? For instance, in order to fabricate the mini-modules, the gold electrode need to be grown on top of the active area, which is not a concern for any evaporation method; however, the side of the module is about 1.22 µm thick. Ebeam evaporation is not possible to produce such contact metallization requirement.
1c. The mini-modules are designed and fabricated in a way that they are internally connected in series, such as in Fig 2i. and only two electrodes are needed to perform the wired characterization, is this the case?

2) According to the device description the device electrodes produce low and high potential at the ITO and gold, respectively. When stimulating cells, for instance, the two electrodes produce an electric field that drives the cell stimulation. In this case, the geometry of the device points out that this electric field is buried inside the device. In this case, it is not obvious how the device electrode geometry produces electric fields that extend into and stimulates the tissue. In fact, authors do use interdigitated electrodes that are connected to their device to produce different levels of electric field on myocardial cells in their in-vitro validations. However, it was not explicitly described in the text nor the SI methods. It was until I saw Fig S5 that I realized this was the configuration.

3) In contrast with the in-vitro experiment where the OES device works as energy harvester and connected to the interdigitated electrodes to produce the stimulation to cells, the OES device interfaces directly with the heart it is the gold electrodes that produce the stimulation. In this case, the in-vitro validation is not relevant to indicate the OES produces electric field into tissue in vi-vivo because the conditions are very different.

4) Although the results are exciting, this reviewer lost enthusiasm when realizing there is not a clear discussion or consideration how the device would be capable to operate robustly in-vivo when considering biological conditions. For instance, devices suffer from fouling due to biofluids around it and scar tissue formation, which is common in implantable devices. Here, the device is not encapsulated by any means, which seems a good thing at first, but is this relevant for demonstrating a translatable technology of this nature?

Minor concerns:
1) It was not clear what is the direction of the device. On one side there is 80nm of gold, whereas on the other side there is 8 or 40 µm of PI substrate. I assume the gold side in on contact with the tissue. This way, light impinging from the PI substrate will be attenuated the less before light reaching the active area. If this is the case, authors must clearly describe the operation.

2) Following up on the previous comment. It is not explicitly written what is the thickness of the PI used for the devices. It is mentioned that there was improvement in stress and strain when reducing the PI film thickness, but not what the final thickness was for the devices. In fact, authors omit key technical information like total thickness of the device, the area of the devices, etc.

3) Authors demonstrate that 780 nm light penetrates through freshly excise skin and fat, which is expected considering the EQE reported and the low optical absorption of tissue in the near infrared band. However, how much is the thickness of this tissue? In addition, the heart is protected by the ribcage, such as in humans. The authors do not discuss, and support with experiment, how thick the tissue should be to replicate the intended tissue thickness of the animal models, including humas. In addition, the effect of the ribcage bone and cartilage is not mentioned nor studied at all nowhere in the text or SI.

4) The viability experiment that uses Sb_2Se_3 co-cultured with H9C2 cells and NRCMs did not include antimony. What is the rationale of excluding antimony considering its toxicity?

5) Based on required implantation conditions for a device of this nature (long implantation), these devices will remain in

contact with moisture and under body temperature conditions. These two important parameters were not included in any in-vitro experiments, thus there is not a prediction of how long these devices could operate in-vivo. For example, what is the performance of the device when it remains under biological conditions for long time, say at 37C under saline buffer?

6) The rat myocardial infarction model, according to the manuscript, uses 1hr of optical stimulation every week. Authors report that the Light-OES group demonstrated robust ventricular contraction and preserved wall motion at week four from implantation. Is one hour a week, 4 hours in that period, enough to achieve the results? Following on my major concern 1 above the descriptions of the in-vivo experiments should be more detailed, perhaps a chronological graph that indicates implantation, stimulation, validations, etc., which is typical in these types of animal experiments.

Reviewer #3

(Remarks to the Author)
Your manuscript presents an innovative and well-executed study demonstrating an ultraflexible optoelectronic stimulator (OES) based on $(Bi,Sb)_2Se_3$ for weak-light–driven modulation of cardiac tissue. The work successfully integrates material-level flexibility, high-efficiency photoelectric conversion under near-infrared illumination, and in vivo demonstration in both rat and swine models. The results provide compelling evidence that the proposed OES platform can restore myocardial conduction and improve cardiac function under low-power stimulation, while maintaining performance across scalable device geometries. This combination of weak-light sensitivity, cardiac applicability, and scalability will be of substantial interest to the bioelectronics and cardiovascular engineering communities. While the study is strong and highly promising, several aspects require clarification and improvement to fully support the translational narrative, particularly regarding device uniformity, mechanical durability, and long-term biostability. If the authors successfully address the major and minor comments outlined below, I believe the manuscript would be suitable for publication in Nature Communications, as it presents a meaningful bioelectronic platform with clear clinical relevance and technological novelty. I therefore recommend addressing the following points for revision.

Major comments
1. In Figure 1, the current explanation appears insufficient to convey the overall research objective and the operating principle of the device. Since Figure 1 serves as a conceptual schematic introducing the study, more detailed descriptions for each caption would be better. I suggest clearly describing the functional significance of each panel and what advantage it represents.

2. In Figure 2a, b, although it is stated that photocurrent mapping was performed under illumination from multiple orientations, the methodological details remain unclear. Specifically, it is not clear what pixel size or device area was mapped, nor by what measurement approach the mapping was conducted. With the current figure alone, it is difficult to interpret the spatial resolution or how the mapped distribution corresponds to the device structure. Therefore, additional clarification or figure improvement appears necessary.

3. The mechanical bending test appears to have been conducted considering the curvature of the cardiac environment. However, 1000 bending cycles seem insufficient to evaluate reliability under an implantable cardiac setting. Since the heart undergoes continuous contraction-relaxation cycles, a minimum of approximately 10,000 bending cycles would be required to substantiate claims regarding mechanical durability.

4. This study includes biocompatibility assessments, but data regarding the biostability of the device and its operational lifetime in an implant environment are not presented. For implantable bioelectronic devices, it is important to demonstrate how long the device can maintain functionality under physiological conditions. Therefore, the lifetime of device assessment would be necessary.

5. This research presents data showing that the electrical output varies with optical stimulation intensity. However, there are no data demonstrating whether uniform output is maintained across individual device cells. For expandable platforms such as this one, spatial uniformity is an important characteristic, and in applications involving multi-site stimulation or large-area operation, cell-to-cell variation could significantly influence stimulation outputs. Therefore, providing comparative photocurrent or voltage output data across individual cells would support the reliability and scalability of the device.

6. The main theme of this study is the capability of the OES to convert tissue-attenuated weak light into electrical signals for cardiac pacing and myocardial regeneration. While Figure 5 demonstrates the regenerative effects, the demonstration of cardiac pacing presented in Figure 6 was conducted under open-chest conditions in a porcine model. I acknowledge the technical difficulties associated with performing closed-chest stimulation in large animal models. However, to validate the device's advantage, I recommend that the authors include additional in vivo pacing data (just like Figure 6g, h, i) in rat or other small animals, but in closed-chest conditions.

Minor comment
1. Several minor text-related corrections appear to be needed. In Figure 2c, the label "Pmax" is clipped, and in Figure 2f, part of the "Voltage (V)" label is also cut off. In Figure 3d, the "Curvature radius (mm)" text is clipped, and in Figure 3i, the "Voltage (V)" label is partially cut off as well. In Figure 4d, the term "2mm skin" should be written with a space as "2 mm".
2. Current description of Figure 2c in the main text could lead readers to misinterpret the figure as plotting actual measured data. I suggest revising the main text to explicitly state that Figure 2c is a schematic representation of device's working

principle.
3. In Figure 2k, it can be inferred from the context that the device is composed of six cells; however, the figure alone does not clearly indicate how many cells are connected in the module. It would be helpful to explicitly indicate the number of integrated cells within the figure.
4. The text discusses stress distribution and cites figure 3b, but I guess it should be figure 3c. Please correct this citation.
5. In Figure 3b, the presented image does not sufficiently show conformal contact between the device and the tissue. Providing a closer or higher-resolution view of the interface would better substantiate the conformal adhesion described in the manuscript.
6. In Figure 4c, y-axis variables are not defined either in figure caption or main text. I suggest adding a brief explanation.
7. When examining the graphs in Figure 4l, m, n, o, the use of different scales makes it difficult to visually compare the results. It would be preferable to unify the scales and display the data within a single graph using different colors, so that the differences can be interpreted more intuitively.
8. Figure 6a appears to be disconnected from the rest of Figure 6. To maintain the coherence of the figure, I suggest moving the fabrication schematic to the supplementary information. Furthermore, the current illustration in Figure 6a is bit hard to understand. It would be more effective to replace it with a clear, step-by-step diagram that shows sequential deposition

Version 1:

Reviewer comments:

Reviewer #1

(Remarks to the Author)
Overall, the authors have greatly improved the manuscript and presented additional experimental results that strongly support the initial evidence. The authors' considerable effort in addressing the previous comments is highly appreciated. The manuscript would certainly be very interesting for the community working on novel materials for biointerfaces. The manuscript just needs a few minor revisions and should be ready for publication.

In detail, the authors have substantially improved the device discussion by presenting additional SEM and TEM results for the material characterization as well as including the additional details on the device fabrication and device characterization. The thermal analysis of the devices looks quite promising and certainly encouraging. The cyclic bending test and post analysis with SEM also look very encouraging. Similarly, analysis of the device stability in the physiological conditions looks very promising. Phototoxicity tests are also very helpful to strengthen the study.

Minor corrections:
1. Main text:
a. Page 3 please specify the PI thickness in the text, i e 8um, as this is critical information for the readers
b. For Fig 1 caption, please specifically not that there is no physical separation between the layers as it can be very misleading
c. For Fig 2 caption please specify the size of the spot used for the device mapping a,b , please specify in the caption that for e onwards it's not focused, still it would be useful to know the size of illumination, are we talking about 5x5mm or 1x1mm or 1x1cm. This certainly makes a difference for reproducibility of the reported results.
d. for the cardiac pacing, it is interesting that the device does not show any slippage from the beating heart, was it sutured or adhered with some gel? as it reads at the moment, the device is just placed on top of the tissue directly, however one would wonder how the adhesion to the tissue was promoted.

2. Supplementary, 1 Device fabrication:
a. For the gold electrode deposition, please include the thickness of gold deposited, right now it says 80um, which is either a typo (i e 80nm) or a size of one of the squares.
3. Supplementary, 2 Photocurrent mapping:
a. What was the spot size? i e 10um or 1mm? this helps to interpret correctly the presented photocurrent maps. Additionally it helps to understand the regime of illumination used to extract the Voc parameters, please also include this information in the caption for fig 2 as discussed above.

Reviewer #2

(Remarks to the Author)
This reviewer acknowledges the authors' efforts to improve the clarity and readability of the manuscript by adding much needed technical details. The revised version addresses most of this and other reviewers' previous comments; however, a fundamental conceptual weakness remains regarding the device architecture.

As described on page 4 (end of the first paragraph), the photodetector architecture generates photocarriers that accumulate at the low-potential (ITO) and high-potential (gold) terminals. While this effect is correct in an optoelectronic device, it raises a critical concern (initially raised in major comment 2 in the previous review report) regarding the spatial distribution of the resulting electric field. Because the generated potential appears to be primarily "buried" within the film, or in-between the two electrodes, there is no evident overlap between the electric field and the target tissue. This reviewer acknowledges that this

lack of field penetration is not an problem for the in vitro experiments, where the photodetector functioned as an energy harvester connected to the interdigitated electrodes. The authors must provide a clear, physical description of how the electric field generated within the photodetector is projected externally to reach the tissue in contact with the gold electrodes.

Reviewer #3

(Remarks to the Author)
I have carefully reviewed the authors’ responses and the revised manuscript. The authors have addressed all of my concerns with great diligence and provided compelling experimental evidence to support their claims. In particular, the main figure has been significantly improved, and the updated conceptual schematic now allows readers to readily understand the advantages of the device.
In addition, the main conceptual image in Fig. 1 has been effectively refined, and the design rationale and operating principle of the device are now conveyed more clearly. From the perspective of mechanical reliability, the bending tests have been sufficiently extended, and the durability under repeated deformation has been well validated. Furthermore, the in-vivo chronic tests conducted over a certain period provide a systematic evaluation of the device stability and functional retention in a biological environment.
Moreover, the spatial uniformity of the device has been appropriately demonstrated by presenting individual cell performance, thereby confirming both cell-to-cell uniformity and batch-to-batch reproducibility. The authors have also recognized the limitations of the open-chest condition and further validated the device performance under closed-chest conditions, effectively demonstrating its applicability in more physiologically relevant scenarios.
In addition, the inclusion of extensive data on mechanical durability and biostability has significantly strengthened the overall reliability of the platform. The methodological details and previously ambiguous expressions have also been clearly clarified. I believe that the manuscript has been sufficiently improved to meet the high standards of this journal. In conclusion, the manuscript is now suitable for publication.

Open Access This Peer Review File is licensed under a Creative Commons Attribution 4.0 International License, which permits use, sharing, adaptation, distribution and reproduction in any medium or format, as long as you give appropriate credit to the original author(s) and the source, provide a link to the Creative Commons license, and indicate if changes were made.
In cases where reviewers are anonymous, credit should be given to 'Anonymous Referee' and the source.
The images or other third party material in this Peer Review File are included in the article’s Creative Commons license, unless indicated otherwise in a credit line to the material. If material is not included in the article’s Creative Commons license and your intended use is not permitted by statutory regulation or exceeds the permitted use, you will need to obtain permission directly from the copyright holder.
To view a copy of this license, visit https://creativecommons.org/licenses/by/4.0/

Point-to-point response to each reviewer:

Reviewer #1

Overall comments:

In this article, the authors present an elegant design for a flexible device proposed to perform cardiac stimulation through optoelectronic conversion. For this purpose, the authors propose using a p-n junction formed between ZnO and (Bi,Sb)2Se3 which is supported by the flexible substrate for the increased biocompatibility. The device relies on efficient conversion of infrared light, 780nm, into open circuit voltage by the pn-junction which is then activates cardiac cells. The device is well characterized in dry conditions, followed by in vitro and animal testing. Overall the device is showing very interesting behavior. As a result, this article will certainly be interesting to the wide audience of Nature Communications. However there are a few major corrections that have to be addressed to reach the necessary quality level of the journal. At the moment, there are missing parts of fabrication and measurement details which do not provide sufficient information for reproducibility purposes. There also a few questions regarding the device performance in biological environments, such as (i) device stability as the active layer is in direct contact with the tissues and liquids and (ii) phototoxicity of the device and induced heating effects. Hence, my recommendation is to accept the manuscript with major corrections.

Major Comments:

Major-1. Fig. 1 schematic suggests that the material fully opens up upon bending, if this is the case such materials would be expected to have quite low stability in the biological environment. While authors refer to the material as vdW layers, there is no either references provided or TEM measurements provided to confirm the exact structure of the material. Additionally, there is very little SEM images provided to characterize the material quality, there is only a small inset in fig 2d. Please provide SEM images at lower magnification to demonstrate the film quality over large scale for the supplementary. Furthermore, the conclusions that there are pertrusions going all the way down in the film are not very well supported. The side view SEM of the film shows that it is polycrystalline certainly. Perhaps authors have more data to support this or more references.

Response:

We thank the Reviewer for this detailed and constructive comment. We clarify that the "fully opened" appearance in **Fig. 1** is a schematic intended only to convey the quasi-1D chain structure and the weak inter-chain vdW coupling in $(Bi,Sb)_2Se_3$, rather than a physical separation of the film. The strong covalent bonds within $(Sb_4Se_6)_n$ chains and the weak vdW bonds between chains endow it with significant mechanical anisotropy and high strain tolerance. Together with its chemical stability, these features confer superior *in vivo* stability compared with conventional 3D optoelectronic materials. To verify, we have added high-resolution transmission electron microscope (HRTEM) characterization as shown in **Fig. R1**, which resolves the chain-like features with [001] direction and reveals an inter-ribbon along *b*-axis pacing of ~3.99 Å[1].

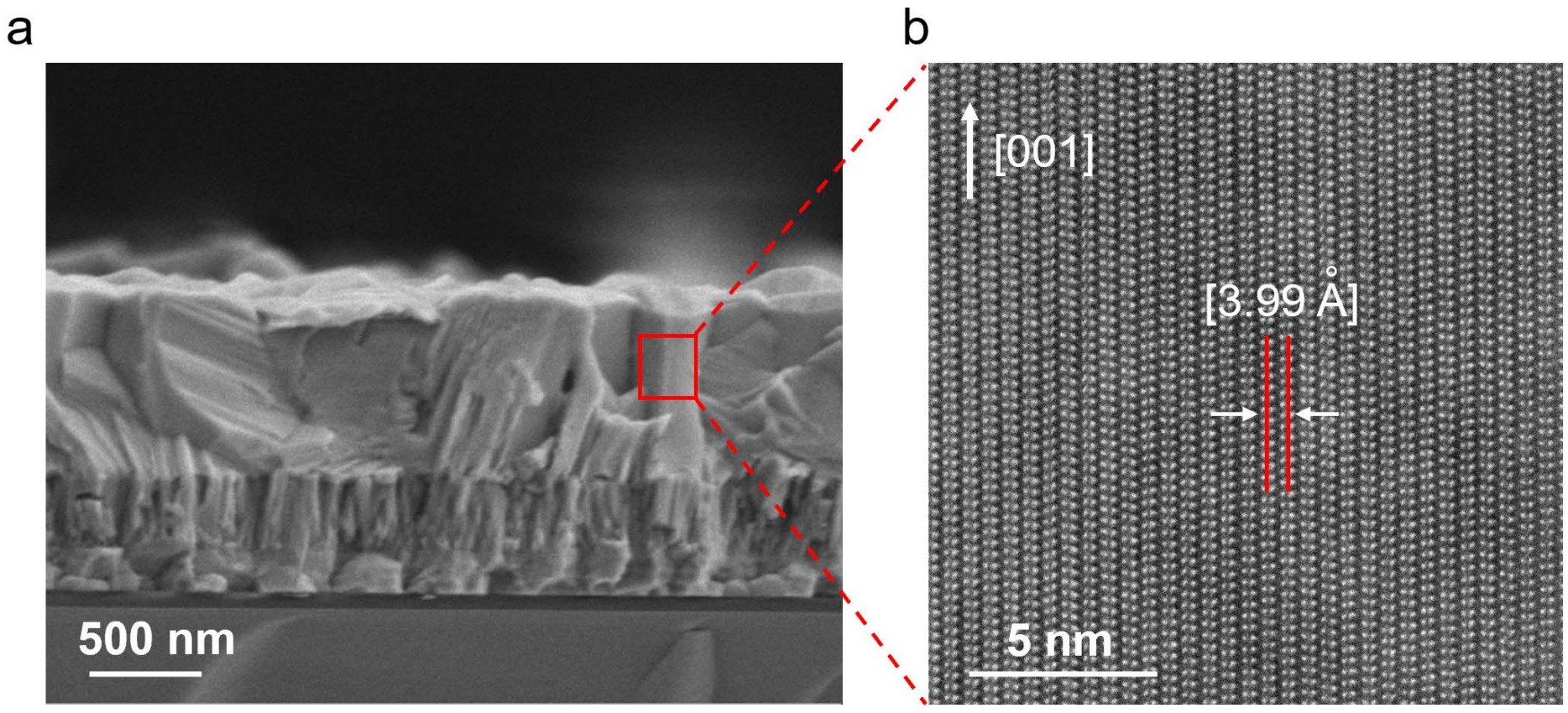

Fig. R1. Morphology of (a) vertically oriented columnar grains in the cross-section of the $(Bi,Sb)_2Se_3$ layer, and (b) the corresponding magnified HRTEM image.

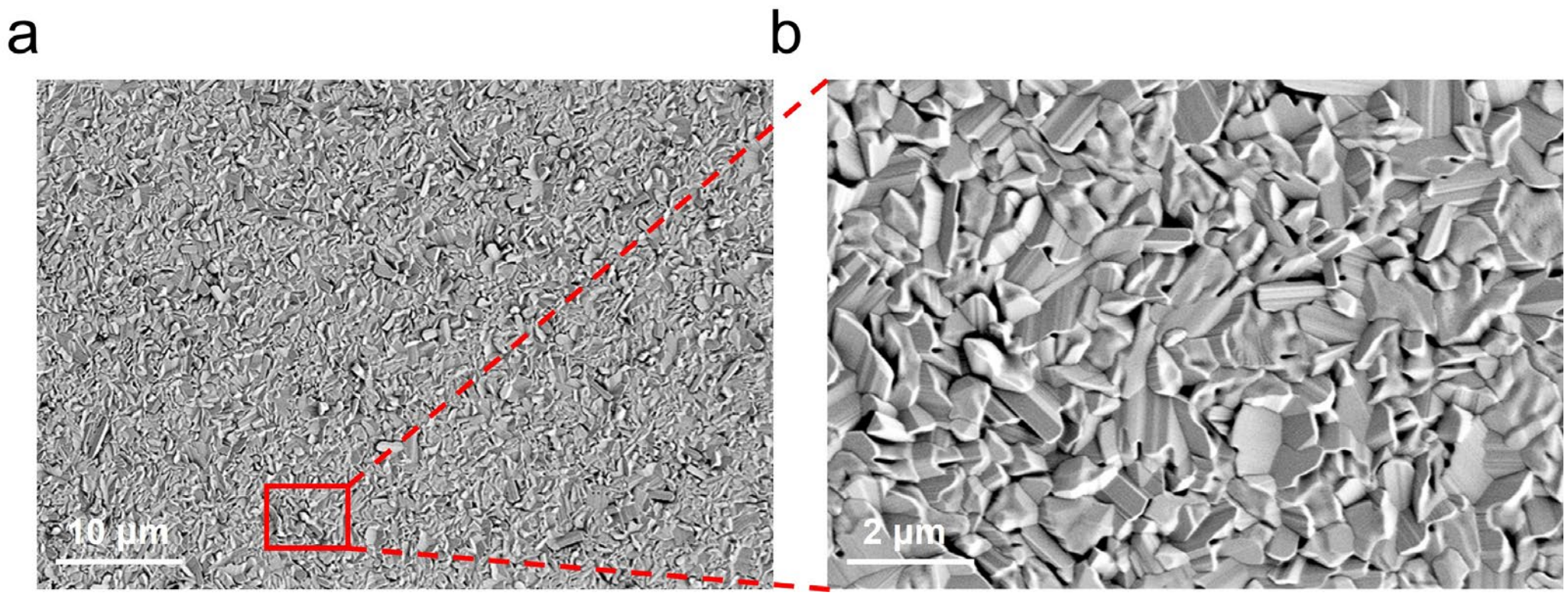

Fig. R2. Plan-view SEM images of the surface morphology of the $(Bi,Sb)_2Se_3$ film: (a) low-magnification and (b) high-magnification views.

Moreover, as shown in **Fig. R2**, we added low-magnification plan-view SEM images demonstrating that the $(Bi,Sb)_2Se_3$ film is uniform and compact over large areas.

Finally, as the Reviewers pointed out, the $(Bi,Sb)_2Se_3$ prepared in this work is not

an ideal [001]-oriented single crystal film. It contains both grains that are completely perpendicular to the substrate and grains with certain tilt angles. However, overall, the film is highly textured [*lk*1] polycrystalline with vertically oriented columnar grains (supported by cross-section SEM and XRD texture in **Fig. S4a**). In the added TEM (**Fig. R1**), the "protrusions" that are completely vertically penetrating to the deep part were selected and added to the revised manuscript to further support the microstructural interpretation.

To address this comment, **Fig. R1**and **Fig. R2** have been added as **Fig. S3** and **Fig. S4 g,h**, respectively. We also revised the **Fig. 1b** caption in the revised manuscript to avoid misunderstanding.

Major-2. In the Figure 2b, the authors provide the current mapping of the device. In general, it is indeed a great way to understand the responsivity of a photodiode. However, the authors do not provide an explanation of where the electrodes are connected or what the maps are. There is no scale bar, there is no explanation how far off the gold pad they are illuminating, there is also no note about the intensity of laser source they are using.

Response:

We thank the Reviewer for the valuable suggestion. This question is similar to Reviewer 3/Major 2. The experimental details have been clarified in the revised manuscript. Specifically, a 780 nm LED (intensity: 24.7mW cm^{-2}) was focused by a convex lens to form a localized spot on a device with a 3×3 electrode array. The light source was positioned 4 cm above the device. The focused light spot was controlled to scan across different positions on the array, and at each position the photocurrent signals from all nine electrodes were recorded simultaneously. The currents were monitored using a semiconductor device parameter analyzer (Agilent B1500A) in a shielded enclosure.

The acquired current data were subsequently subjected to smoothing to enhance spatial continuity, and the processed data were used to construct spatially resolved photocurrent 2D mapping, enabling visualization of the localized illumination position. The nine mappings shown in **Fig. 2b** represent the different illumination positions on the 3×3 electrode array, in sequential order from left-to-right and from top-to-bottom. The mapping results demonstrate that the fabricated device possesses precise spatial addressability, allowing site-specific photoelectric stimulation as required.

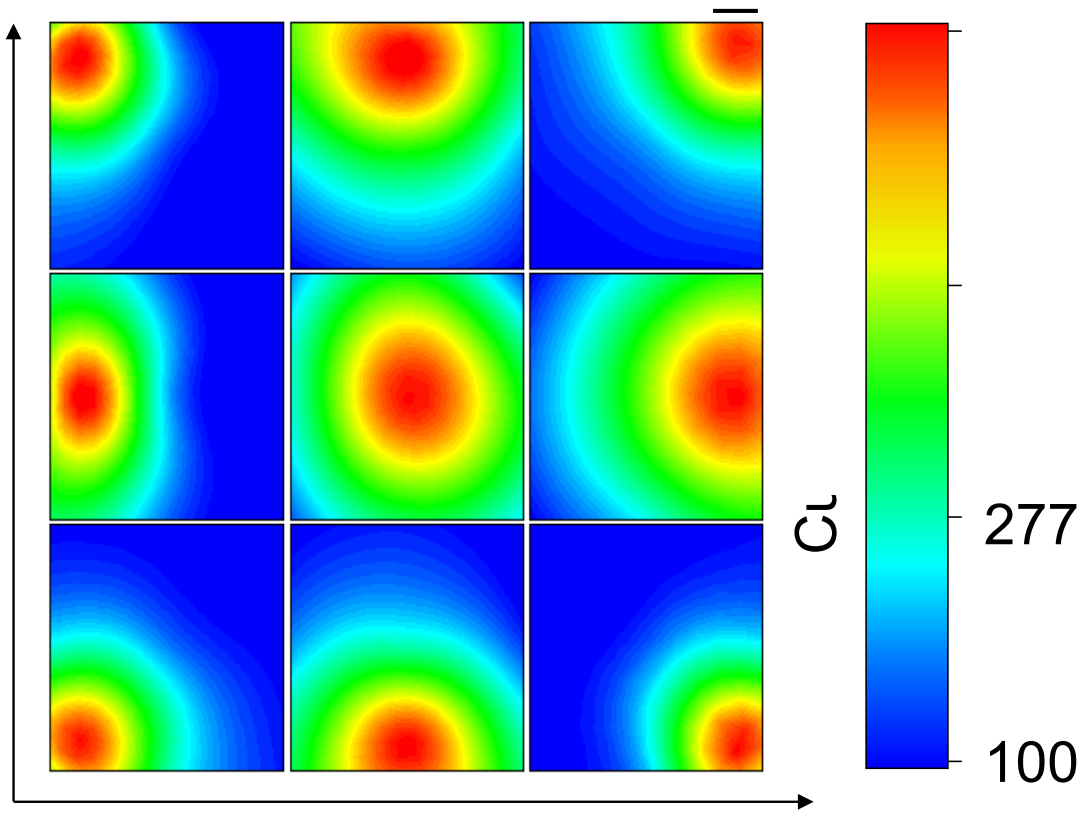

Fig. R3. 2D photocurrent mapping under 780 nm illumination from multiple orientations, demonstrating the high spatial resolution of the OES. The nine panels correspond to nine illumination positions ordered left-to-right, top-to-bottom.

To address this comment, we added a scale bar to **Fig. 2b**, and also expanded the description and experimental detail for **Fig. 2b** in the Methods section and revised figure caption.

Major-3. Figure 2c shows a typical diode performance curves, however this is very unclear if those are measured for this specific device. If they are measured for this particular device, there should be explicit numbers for current and for voltage, furthermore, there is no reference or explanation what abbreviations provided in the graph mean, which can make it hard to follow. the paper for people coming from a broad range of backgrounds.

Response:

We thank the Reviewer for pointing out the ambiguity. This question is similar to Reviewer 3/Minor 2. In the original version, **Fig. 2c** was intended as a schematic illustration to define the classical photovoltaic parameters (*I*-*V* and *P*-*V* characteristics) rather than a measured curve, which indeed may lead to confusion.

To address this comment, we have therefore revised **Fig. 2c** and its caption to (i) explicitly state that this panel is a schematic plot, and (ii) define all abbreviations used in the schematic figure (I_{SC}, short-circuit current; V_{OC}, open-circuit voltage; M_{PP}, maximum power point; V_{mpp}/I_{mpp}, voltage/current at maximum power point; P_{max}, maximum output power). These changes improve readability for readers from diverse backgrounds.

Major-4. Figure 2 e-h, it is unclear how the devices have been characterized. Given, that the device mapping in 2b are highly non-uniform with a current varying at least 6 times across the device, it is not clear how those performance metrics have been measured then. Was the position of the 780nm diode illumination positioned at the point of maximum photocurrent? If so, this should be clearly stated. Given that a 780nm diode is used for the measurements is focused using a lens, what is the illumination spot is reached based on FWHM?

Response:

We thank the Reviewer for this insightful comment. We clarify that the non-uniform current distribution in **Fig. 2b** reflects the intrinsic spatial variation of the device response, as discussed in Comment #2 above. As the Reviewer noted, the locations of maximum photocurrent in Fig. 2b correspond to the illumination positions of the focused light spot (diameter=3 mm). Only the experiment in **Fig. 2b** used a lens to focus the light, which was intended to demonstrate the photodetector's controllable spatial response for potential *in vivo* implantation. In contrast, no focusing optics were used for the measurements in **Figs. 2e-h**, which were performed with direct LED illumination.

To address this comment, we have clarified the discretion of **Fig. 2b** in the revised manuscript. Furthermore, the characterization of the devices shown in **Figs. 2e-h** was performed as below and has also been added to the Methods section:

EQE test

To measure the external quantum efficiency (EQE) of the optoelectronic stimulation devices, broad-spectral white light from a xenon lamp (300 W, Oriel, 69911, Newport) was dispersed into monochromatic light using a monochromator (Oriel cornerstone ™ 130 1/8, Newport). The intensity of monochromatic light was calibrated by a standard silicon photodetector. The photocurrent was recorded using a pre-amplifier and a lock-in amplifier. The EQE spectrum can be obtained according to the light intensity and photocurrent. Then, the photoresponsivity (R) was calculated based on the equation[2]:

$$R = EQE(q\lambda/hc) \qquad (1)$$

where q is the elementary charge, λ is the wavelength, h is the Planck constant, and c is the velocity of light.

***I-V* curves test**

The Current-Voltage (I-V) characteristics were measured from -1 to 1 V under dark conditions and 780 nm LED illumination using a semiconductor device parameter

analyzer (Agilent B1500A) in an electrostatically shielded enclosure. Open-circuit voltage (V_{OC}) was measured as a function of illumination intensity.

Open-circuit voltage measurements under pulsed illumination

The open-circuit voltage (V_{OC}) of the OES device was measured using a digital multimeter, with the positive and negative leads connected to the indium tin oxide (ITO) common electrode and the Au electrode, respectively. Pulsed illumination was provided by a calibrated light source at 24.7 mW cm^{-2} with a 50% duty cycle. The pulse frequency was varied (1, 2, and 3 Hz), and the corresponding V_{OC} of the OES was recorded under each condition.

Major-5. The authors show the results for a range of light illuminations, but what is a safe range for tissue illumination and when the heating/damage occurs? "The output voltage was tunable between 0.02 V and 0.22 V by varying the incident intensity from 0.05 to 24.7 mW cm^{-2}".

Response:

We thank the Reviewer for this important comment. The illumination intensity used in this work (0.05-24.7 mW cm^{-2} at 780 nm) lies within the commonly used photo biomodulation irradiance levels (typically ~1-100 mW cm^{-2}) for NIR light sources[3], which is generally considered safe and non-thermal for biological tissues. Thermal damage has been reported to occur only at substantially higher power densities (>600 mW cm^{-2})[4].

Consistently, we experimentally monitored the photothermal effects under our maximum irradiance condition by infrared camera (UNI-T Uti260E). As shown in Fig R4, under 780 nm illumination at 24.7 mW cm^{-2} (pulsed at 1 Hz) for 1 h, the measured temperature remained stable with only a small variation of ~0.5 °C (23.7-24.2 °C), indicating negligible photothermal effects under our experimental conditions.

To address this comment, **Fig. R4** has been added as **Fig. S5**. A discussion on the biocompatibility and photothermal safety of the illumination has been added to the revised manuscript (Page 4, highlight in blue).

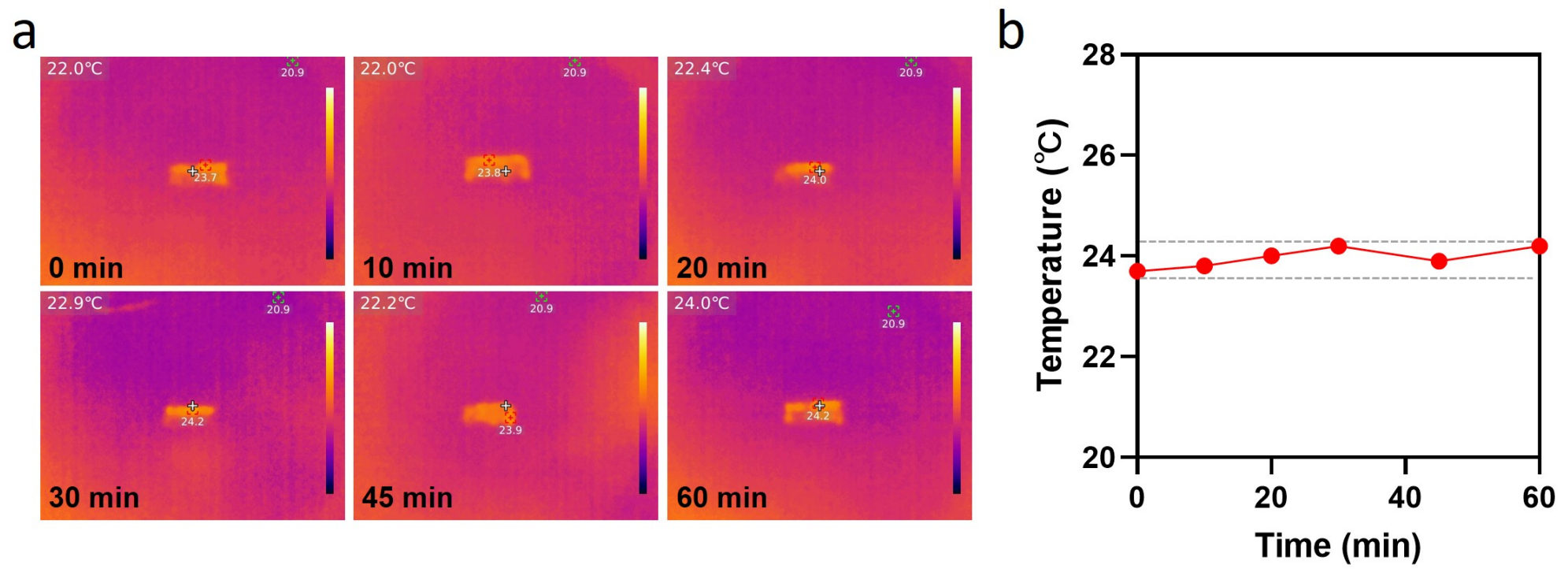

Fig. R4. Photothermal response of the OES under pulsed light illumination. (a) Infrared thermal images acquired at different time points during pulsed light stimulation (24.7 mW cm^{-2}, 1Hz). (The temperature indicated by the red cursor corresponds to the OES, the green cursor denotes the minimum temperature within the captured frame, and the value shown in the upper-left corner represents the average temperature of the entire image.) (b) Statistical analysis of the temperature variation, showing a minimal fluctuation (≈0.5 °C over 1 h), which confirms stable thermal behavior and negligible photothermal effects.

Major-6. There is almost no explanation how the minimodules are fabricated. The general explanation of the unit fabrication is also missing some significant information which can result in very low reproducibility of these results (see more comments on this below). The only minimodule fabrication details are provided briefly in the supplementary and are not sufficient for reproducibility. It is quite important to see the sem images of those minimodules as well. Provided info "For the mini-module fabrication, the three-step laser etching technique was employed, including the P1 etching to cut off the ITO bottom electrode, P2 etching to remove the ZnO and $(Bi,Sb)_2Se_3$ layer, and P3 etching to cut off the Au top electrode." How all of these etching steps are done without damaging polyimide is unclear as well.

Response:

We thank the Reviewer for the valuable comments on the description and reproducibility of the mini-module fabrication process. This question is similar to Reviewer 2/Major 1. In response, we have added a schematic of the micro-module fabrication workflow (**Fig R5**), and expanded the fabrication details as follows:

Laser scribing and module fabrication

$(Bi,Sb)_2Se_3$ mini-modules were fabricated using a standard three-step laser scribing process, following a previously reported procedure with minor modifications[5].

ITO substrates (5 cm × 5 cm) were first patterned by a 1,064 nm femtosecond laser (KW-20W-cx, Wuhan, China) to form the P1 lines (10 W, 900 mm s^{-1}), which served as the bottom electrode. A ZnO buffer layer followed by an $(Bi,Sb)_2Se_3$ absorber layer was subsequently deposited onto the patterned substrates. The P2 scribing (10 W, 1300 mm s^{-1}) was then applied to locally remove the ZnO/$(Bi,Sb)_2Se_3$ layers, exposing the underlying ITO and enabling interconnection between adjacent sub-cells. After deposition of the Au electrode, P3 scribing (10 W, 1,500 mm s^{-1}) was performed to remove the Au electrode and electrically isolate neighboring sub-cells. The final $(Bi,Sb)_2Se_3$ mini-modules were formed by monolithic series interconnection of the individual sub-cells. The scribing depth was precisely controlled by adjusting the femtosecond laser power and the scan speed. Photograph of the micro-modules and corresponding SEM images of representative regions are shown in ***Fig. S7****.*

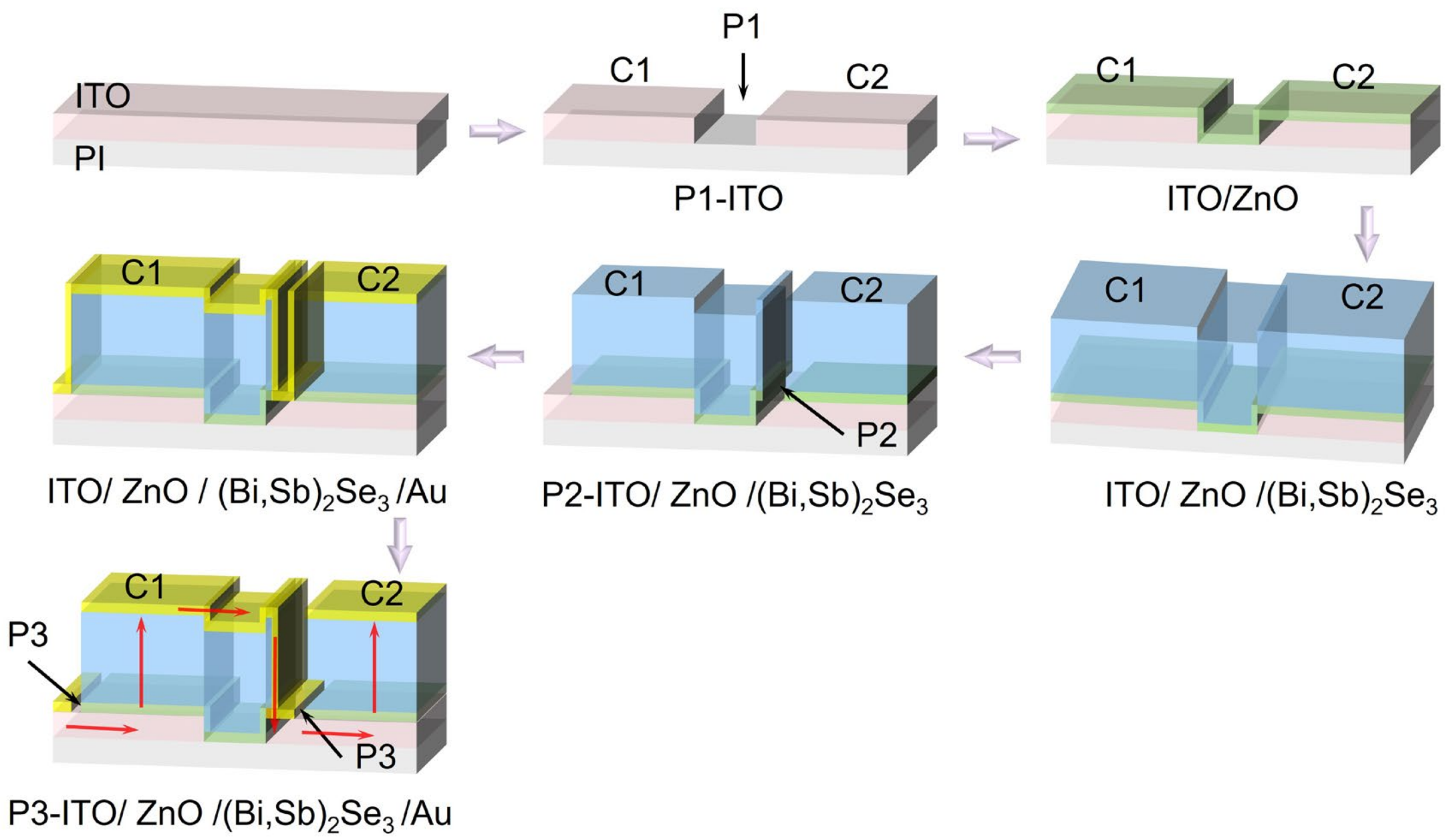

Fig. R5. Schematic of the micro-module fabrication flowchart.

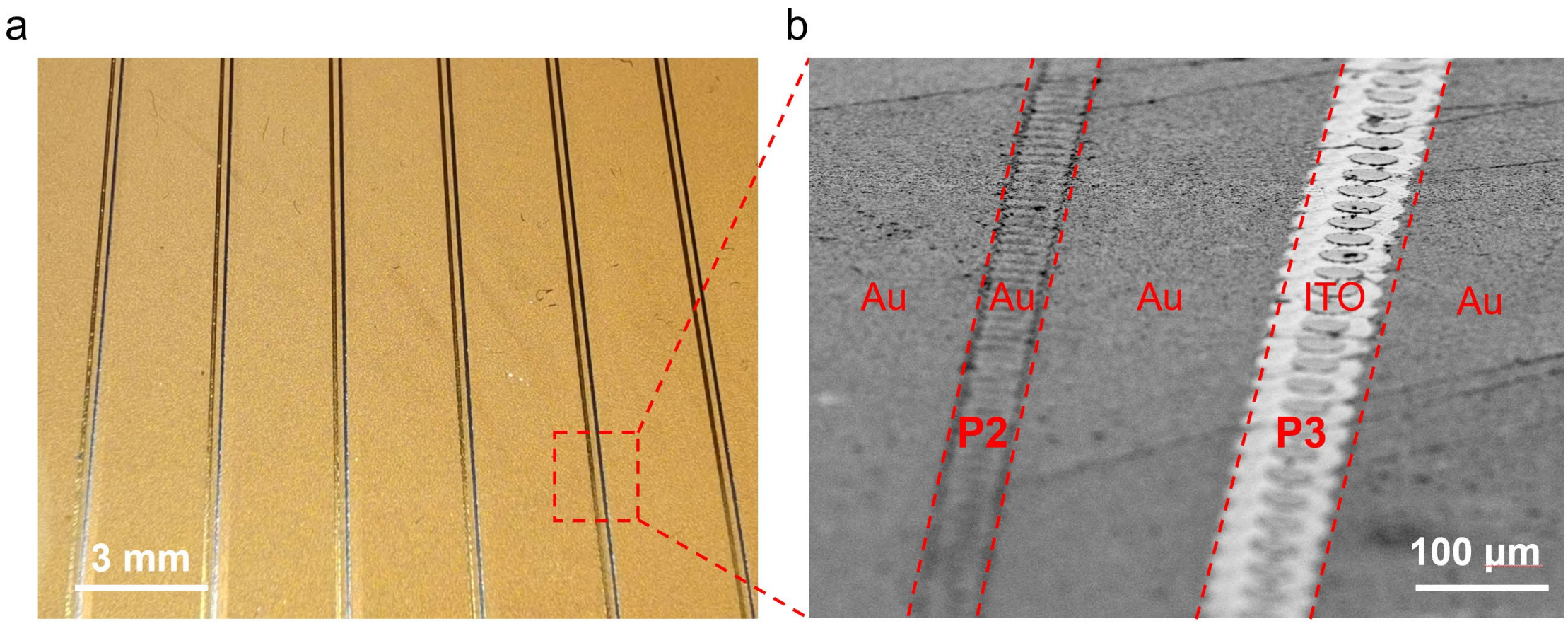

Fig. R6. (a) Photograph of the micro-modules and (b) SEM images of representative regions. The P1 scribe is shallow and is subsequently covered by $ZnO/(Bi,Sb)_2Se_3/Au$ layers, making it almost invisible in the image.

To address this comment, **Fig. R5** and **Fig. R6** have been included in the Supplementary Information as **Fig. S6** and **Fig. S7**, respectively. The fabrication details of micro-modules have been added to the Methods section.

Major-7. Figure 2j what is the light intensity used? Figure 2k barely possible to read the light intensity scale, perhaps use 1, 10, 100 mW/cm^2 but again what are the biological limitations in terms of heating/tissue damage?

Response:

We thank the Reviewer for the helpful comments.

1. Regarding the light intensity in Fig. 2j:

The light intensity used in this experiment is 24.7 mW cm^{-2}, which has now been explicitly stated in the revised **Fig. 2j** and its caption.

2. Regarding the readability of the light intensity scale in Fig.2k:

We have marked the corresponding X-axis values at each data point to improve readability. **Fig. 2k** has been replaced with **Fig. R7** in the revised manuscript.

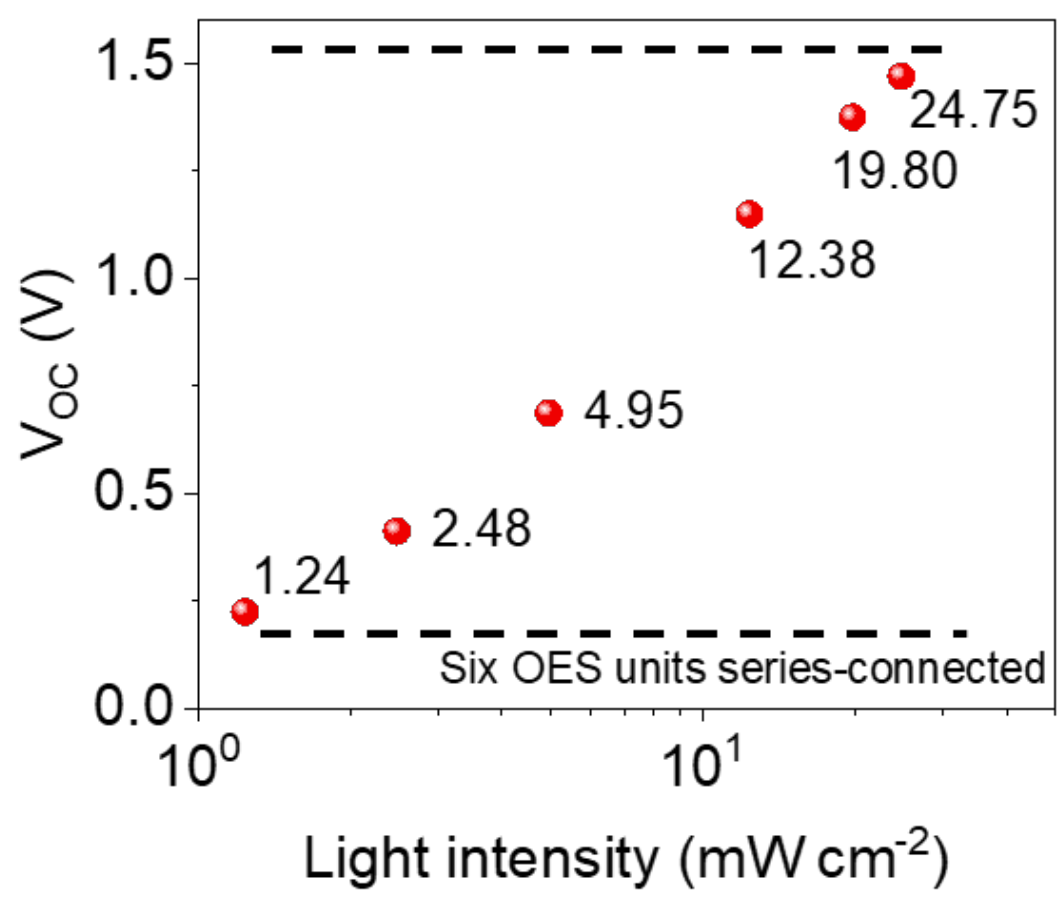

Fig. R7. V_{OC} of a series-connected modules with six cells as a function of incident light intensity. The value at each point indicates the corresponding X-axis light intensity.

3. Regarding biological limitations such as heating and tissue damage:

As discussed in Comment #Major-5 above, the illumination intensity (0.05-24.7 mW cm^{-2} at 780 nm) falls within the range commonly used for photobiomodulation (~1-100 mW cm^{-2}), which is generally regarded as safe and non-thermal for biological tissues. The negligible temperature rise observed experimentally in **Fig R4** further confirms tissue safety.

Major-8. "Fabricated on an optimized polyimide (PI) substrate" there are no details about the thickness of polyimide used nor the fabrication steps used to make it.

Response:

We thank the Reviewer for this valuable comment. This question is similar to Reviewer 2/Minor 2. In this work, the PI substrate was custom-fabricated by Wuhan Yimaide New Materials Technology Co., Ltd with an optimized thickness of 8 μm, which was selected to balance mechanical flexibility and structural stability. We have added the supplier information and the thickness of PI substrate to the revised Method section.

Major-9. How is this measured or estimated? There are no details in the text: "Upon bending, the $(Bi,Sb)_2Se_3$ layer experienced an in-plane strain of ~0.083%, corresponding to a stress of~1.45×10^6 Pa, significantly lower than that in conventional three-dimensional semiconductors such as ZnO and ITO (Fig. 3b)." In figure 3c, there are results of Comsol calculations for 8um PI substrate, is this the thickness used in the paper? There is no mentioning of the actual thickness of the PI layer used for these devices.

Response:

We thank the Reviewer for highlighting this missing methodological detail. The in-plane strain (~0.083%) and stress (~1.45×10^6 Pa) reported for the $(Bi,Sb)_2Se_3$ layer were estimated from finite-element simulations rather than directly measured experimentally. In the revised Experimental Section, we have explicitly described the detail:

FEA modeling: Finite element models were built in COMSOL Multiphysics 6.1 to evaluate the mechanical responses of the flexible photoelectric devices. A multilayer structure consistent with the fabricated device geometry was constructed. An in-plane compressive load was applied to the side of the film, and the loaded surface was constrained to allow only in-plane displacement. The strain at the center of the $(Bi,Sb)_2Se_3$ layer was extracted, and the corresponding stress was obtained from the same simulation results. A hybrid mesh combining quadrilateral and tetrahedral elements was used for computation. Finite element parameters used in the model, including thickness, Young's modulus, and Poisson's ratio for each functional layer are detailed in **Table S1**.

Importantly, the PI substrate thickness used in the fabricated devices is 8 μm, and the COMSOL model in **Fig. 3c** uses the same thickness.

To address this comment, we have added the simulation detail to the revised Methods section.

Major-10. Re the mechanical resilience, it is quite important to check the active material properties post-bending with SEM, those would show if there was any material delamination or significant crack propagation. "The mechanical resilience of the OES arises from the intrinsic van der Waals bonding and benign grain boundaries within the one-dimensional crystal framework of $(Bi,Sb)_2Se_3$, which confer exceptional tolerance to deformation and structural imperfections." Minor: Why there is an intrinsic vdW bonding, please provide references?

Response:

We thank the Reviewer for this insightful comment. This question is similar to Reviewer 3/Major 3. To evaluate the potential delamination and crack propagation after repeated bending, the flexible OES was subjected to 10,000 bending cycles under a minimum bending radius of 1 mm, as shown in **Fig. R8a**. This condition is substantially more severe than the deformation encountered under normal operating conditions. In situ SEM observation in the bent state revealed no obvious cracks on the surface of the $(Bi,Sb)_2Se_3$ layer (**Fig. R8b**). In addition, SEM images taken after 0, 100, 1,000, and 10,000 bending cycles show that slight surface microcracks appear after 1,000 cycles, while no obvious delamination of the active layer was observed even after 10,000 cycles (**Fig. R8c**).

Despite the appearance of limited surface microcracks after extensive cycling, the optoelectronic output remains largely preserved, decreasing by only 2.8% after 10,000 bending cycles (**Fig. R8d**), and the time-resolved photocurrent response under repeated light on/off cycles remains stable (**Fig. R8e**). These results indicate that the OES maintains good interfacial integrity and crack-tolerant mechanical performance under extreme bending conditions.

This mechanical compliance of $(Bi,Sb)_2Se_3$ is related to its quasi-one-dimensional crystal structure, featuring strong covalent bonding within the $(Sb_4Se_6)_n$ ribbons/chains and weak van der Waals interactions between adjacent ribbons/chains, which gives rise to pronounced structural anisotropy[6,7].

To address this comment, **Fig. R8** has been added as **Fig. S10**. The relevant discussion of mechanical resilience has been added to the revised manuscript (Page 5, highlight in blue), and the corresponding references have been added as Refs. [31,39].

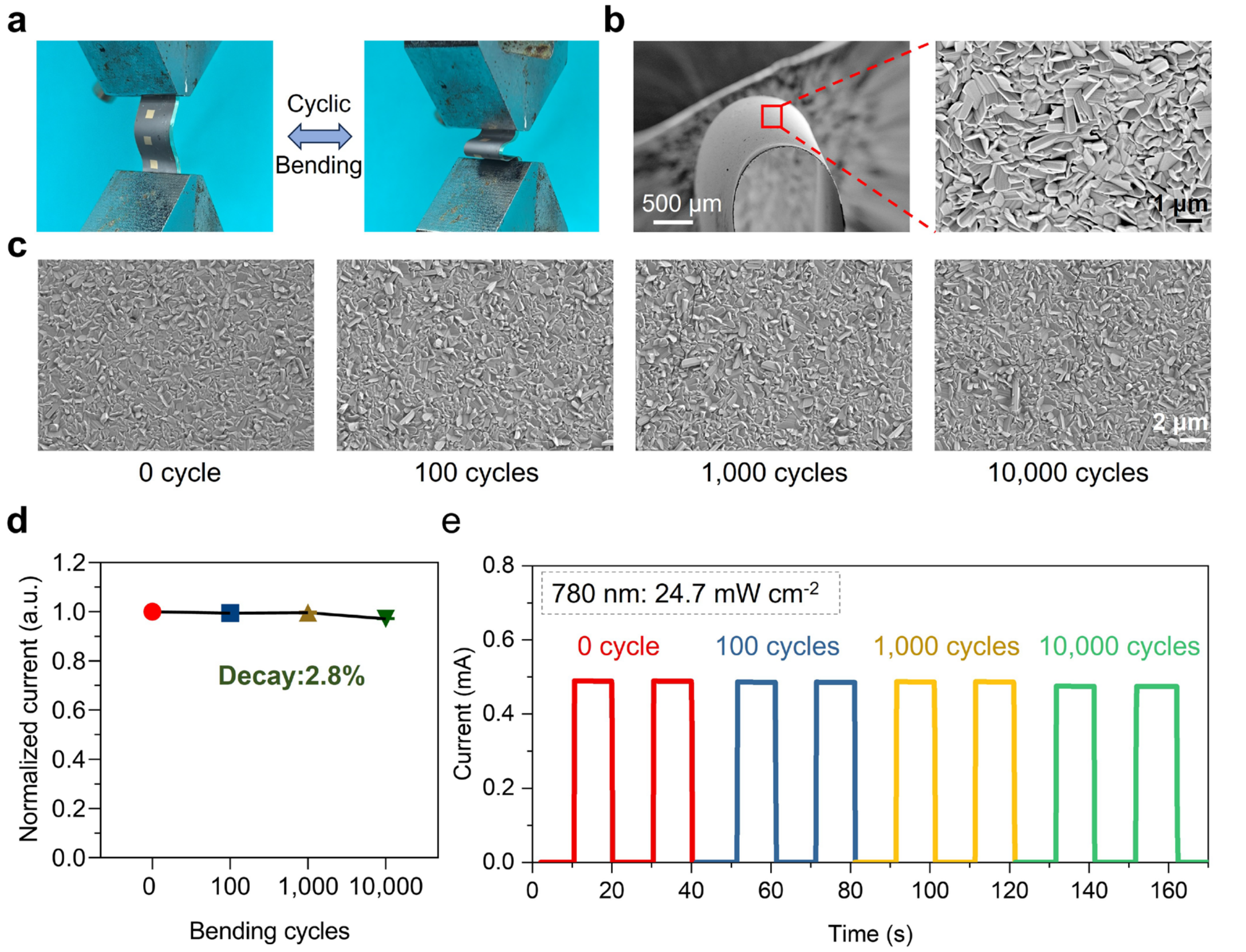

Fig. R8. (a) Photographs of the cyclic bending test. (b) In situ and higher-magnification surface SEM images of the OES in the bent state at a bending radius of 1 mm. (c) SEM images of the OES after 0, 100, 1,000, and 10,000 bending cycles. (d) Normalized photocurrent of the OES measured after 0, 100, 1,000, and 10,000 bending cycles. (e) Time-resolved photocurrent (*I-T*) response of the OES under repeated light on/off cycles (780 nm, 24.7 mW^{-2}).

Major-11. The mechanical robustness curves are quite impressive, however, similarly to the comment number 3 above, it is unclear how this is measure and how the device is connected, based on the photo of the devices, those are not mini-modules from the authors explanations, is this correct? As from the schematics the minimodules appear to have continuous gold electrode.

Response:

We thank you for this comment. The mechanical bending was applied using a motorized tensile tester (CTM6050): OES devices were pre-compressed and then subjected to repeated stretch–compression cycles, the minimum bending radius of 0.5 mm. The tensile tester only imposed the bending; after the desired number of cycles, the device was removed and its photocurrent measured. The *I–V* characteristics were recorded from −1 to 1 V under dark conditions and 780 nm LED illumination using an Agilent B1500A in an electrostatically shielded enclosure. The devices used for mechanical testing were single OES devices, not discrete mini-modules.

To address this comment, the experimental details of the bending cycle test have been added to the revised Methods Section, and we have also clarified the type of device used in all experiments in the revised manuscript.

Major-12. Fig 4 c-e, same comments as N3 and N10, where the illumination is located and how is it calculated.

Response:

We thank the Reviewer for this insightful comment. In Figs. 4c-e, the distance between the light source and the device is fixed at 4 cm in all measurements. This information has been added to the figure captions and the Methods section.

For Fig. 4c, P0 is defined as the incident light intensity without tissue obstruction, whereas P1 denotes the transmitted light intensity after passing through different biological tissues, including 2 mm skin, 2 mm fat, or 2 mm skin + 2 mm fat.

For Fig. 4d, *External quantum efficiency (EQE*)* denotes the external quantum efficiency of the device under illumination transmitted through the tissue layers described above.

For Fig. 4e, EQE represents the external quantum efficiency measured as a function of wavelengths under different incident light intensities.

To address this comment, we revised the captions of Fig. 4c-e to explicitly indicate the illumination direction and conditions, thereby improving the clarity and reproducibility of the results.

Fig. 4c: Comparison of incident (P0) and transmitted (P1) light intensity through different tissue layers (2 mm skin, 2 mm fat, and their combination).

Fig. 4d: EQE of the device measured under transmitted through the tissue layers.*

Fig. 4e: EQE as a function of wavelength under different incident light intensity.

Major-13. Beautiful stimulation results for the cardiac cells observed, minor questions regarding the duration of the culture on the device prior the measurements? The major questions, are the cells survive after such stimulation? i e phototoxicity assessment? Major question, how do cell correspond to the illumination at these powers without the device present, i e is there any direct cell heating at those illumination intensities?

Response:

We thank the Reviewer for the valuable comments. Neonatal rat cardiomyocytes (NRCMs) were cultured on the device for 7 days before measurements to ensure stable adhesion and physiological activity.

Control experiments were performed under identical illumination power density without the device. No discernible effects on the cells were observed, indicating that the illumination alone did not induce direct stimulation or heating (**Fig. R4**), consistent with the thermal effect tests discussed in Comment #5 above. Under the same experimental conditions (varying stimulation frequency and electric field strength), the cells maintained normal morphology, and viability assays confirmed no noticeable phototoxicity under the applied illumination (**Fig. R9**).

Therefore, the observed calcium signaling modulation originates from the optoelectronic response of the device rather than from direct light-cell interactions.

To address this comment, **Fig. R9** and its related discussion have been added as **Fig. S11** and the revised manuscript (Page 6, highlight in red).

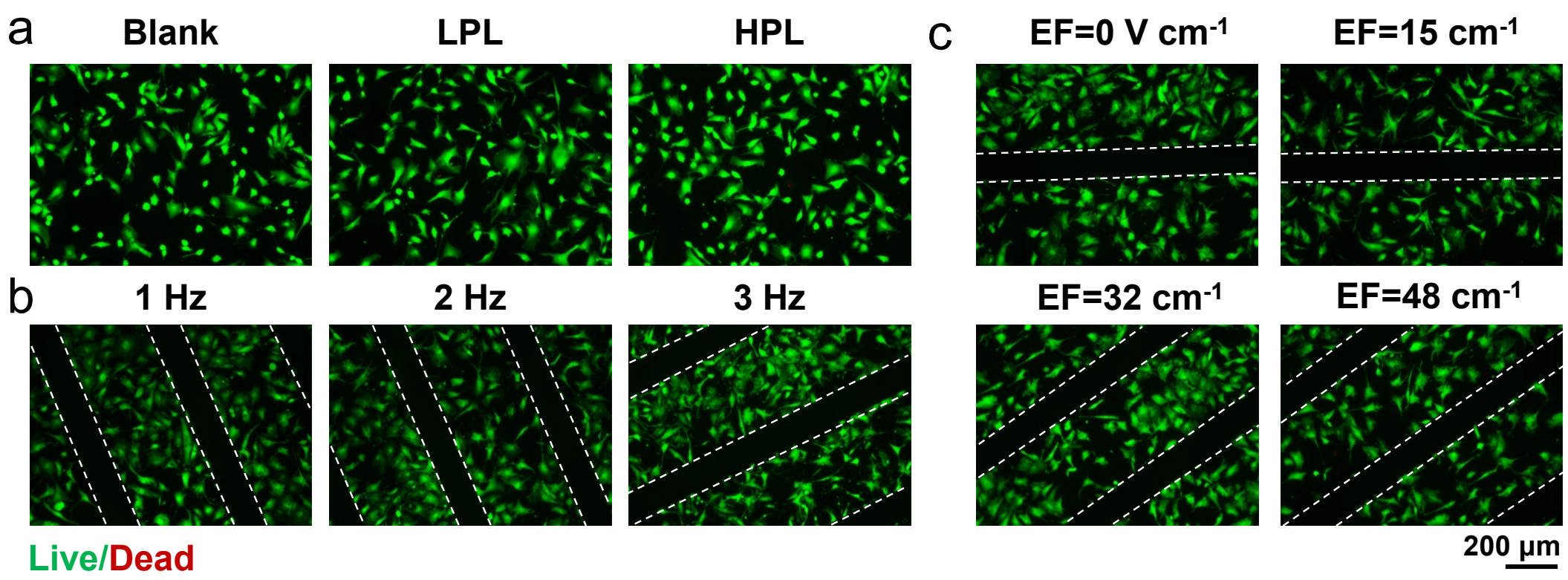

Fig. R9. Live/dead staining of NRCMs under different conditions. (a) NRCMs exposed to optical illumination at identical power density without the device, showing negligible phototoxicity. (b) NRCMs after optoelectronic stimulation at different frequencies. (c) NRCMs after stimulation under different electric field strengths. Dashed lines indicate the electrode positions. (LPL: low power light; HPL: high power light)

Major-14. How does the device look like post implantation? Was there any SEM assessment of the active layers degradation? The active material is in direct contact with the biological tissues, surely this will lead to a significant degradation and delamination, as there is no encapsulation used.

Response:

We thank the Reviewer for this important comment. This question is similar to Reviewer 2/Major 4 and Reviewer 3/Major 4. In the revised manuscript, we added morphology and photoresponse characterization of the OES after 4 weeks of implantation on the SD rat heart.

Optical images show that the OES was encapsulated by a thin fibrotic tissue layer on the heart surface, while remaining structurally intact after removal of the surrounding tissue (**Fig. R10a**). SEM observation of the explanted device show only minor surface cracks in the $(Bi,Sb)_2Se_3$ layers, likely associated with chronic cyclic deformation imposed by cardiac motion, while no obvious large-area delamination of the active layer was observed (**Fig. R10c**). In addition, the photocurrent of the device explanted after 4 weeks showed a decrease of 8.9% compared to the pre-implantation value (**Fig. R10b**). These results demonstrate that the OES maintains substantial structural integrity and photoelectric functionality after 4 weeks of direct implantation, despite the absence of encapsulation.

To avoid overstatement, we have also clarified in the revised figure caption that these results support mid-term *in vivo* stability. Long-term implantation will still require effective encapsulation strategies to prevent degradation caused by prolonged mechanical friction and exposure to biofluids.

To address this comment, **Fig. R10** has been added as **Fig. S21a-d**, and the description of device robustness under biological conditions has been added in the revised manuscript (Page 8-9, highlight in blue).

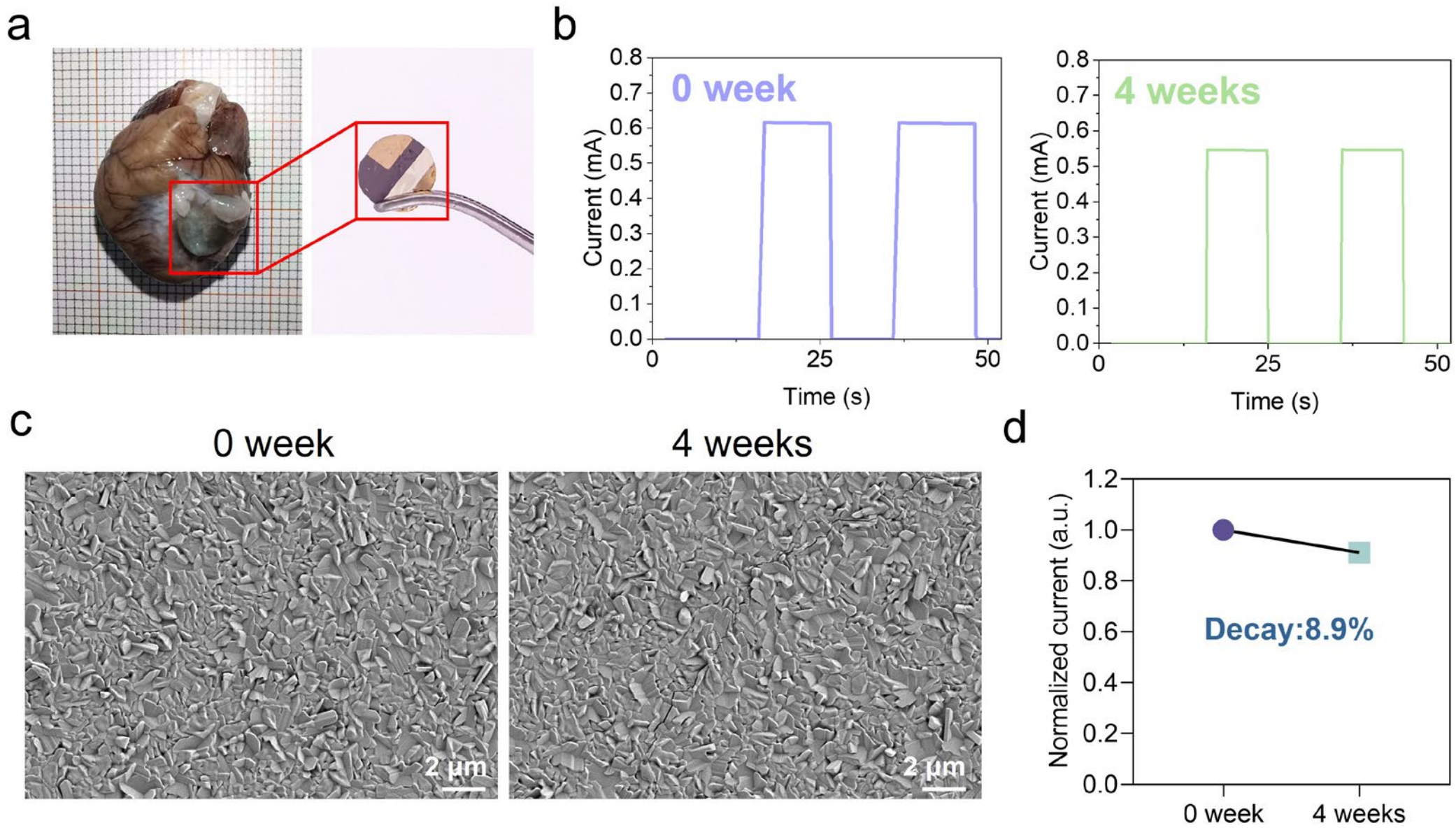

Fig. R10. (a) Optical images of the OES retrieved after 4 weeks of implantation on the SD rat heart. (b) *I-T* curves recorded before and after implantation, under a illumination of 24.7 mW cm^{-2} at 780 nm. (c) SEM images of the $(Bi,Sb)_2Se_3$ layers before implantation and retrieved after 4 weeks, indicating minor surface cracks without obvious delamination. (d) Photocurrent response of the OES after retrieval following 4 weeks of implantation.

Minor comments:

Minor-1. In abstract, the following sentence needs to be paraphrased as the meaning is somewhat unclear, especially for a broad audience of the journal: "clinical translation is hindered by the rigidity and weak-light inefficiency of conventional silicon-based devices" Please specify more explicitly what is the issue of weak-light inefficiency. Perhaps including another sentence on requirements for illumination conditions would be helpful too.

Response:

We thank the Reviewer for this helpful suggestion. In the original abstract, "weak-light inefficiency" was meant to describe a practical limitation of conventional silicon-based implantable optoelectronic devices *in vivo*: after absorption by overlying tissue, the irradiance reaching the implant is often ~μW cm^{-2}, under which silicon photovoltaics may fail to generate sufficient voltage for effective stimulation, thereby necessitating higher illumination that can be impractical and may raise heating and phototoxicity concerns.

To address this comment, we have revised the abstract and clarified the

illumination requirement for clinical translation (i.e., reliable operation under tissue-attenuated, low-power near-infrared light). These edits improve clarity for a broad audience.

Minor-2. For the fig 2b, the scale bar can be in microamperes, hence better readability and no need to x10^-4.

Response:

We thank the Reviewer for this helpful suggestion. As shown in **Fig. R11**, we have revised the color bar in Fig. 2b to microamperes (μA) and removed the $\times 10^{-4}$ notation.

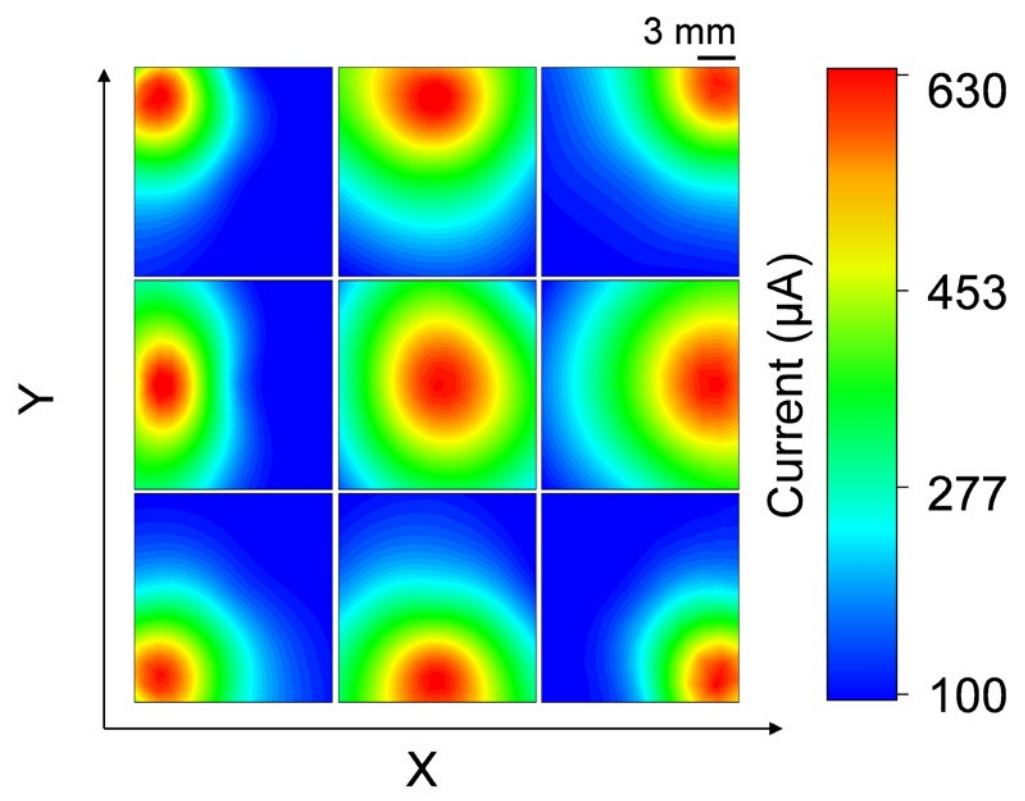

Fig. R11. 2D photocurrent mapping under 780 nm illumination from multiple orientations, demonstrating the high spatial resolution of the OES.

To address this comment, **Fig. 2b** has been replaced by **Fig. R11** in the revised manuscript.

Minor-3. Fig 3c has quite important device information regarding thicknesses of the layers, but those are very challenging to read, similarly to the scale bars.

Response:

We thank the Reviewer for this helpful suggestion. To enhance the readability of the image, we enlarged the font size within **Fig. 3c** and moved the layer thickness information to the figure caption, as shown in **Fig. R12.**

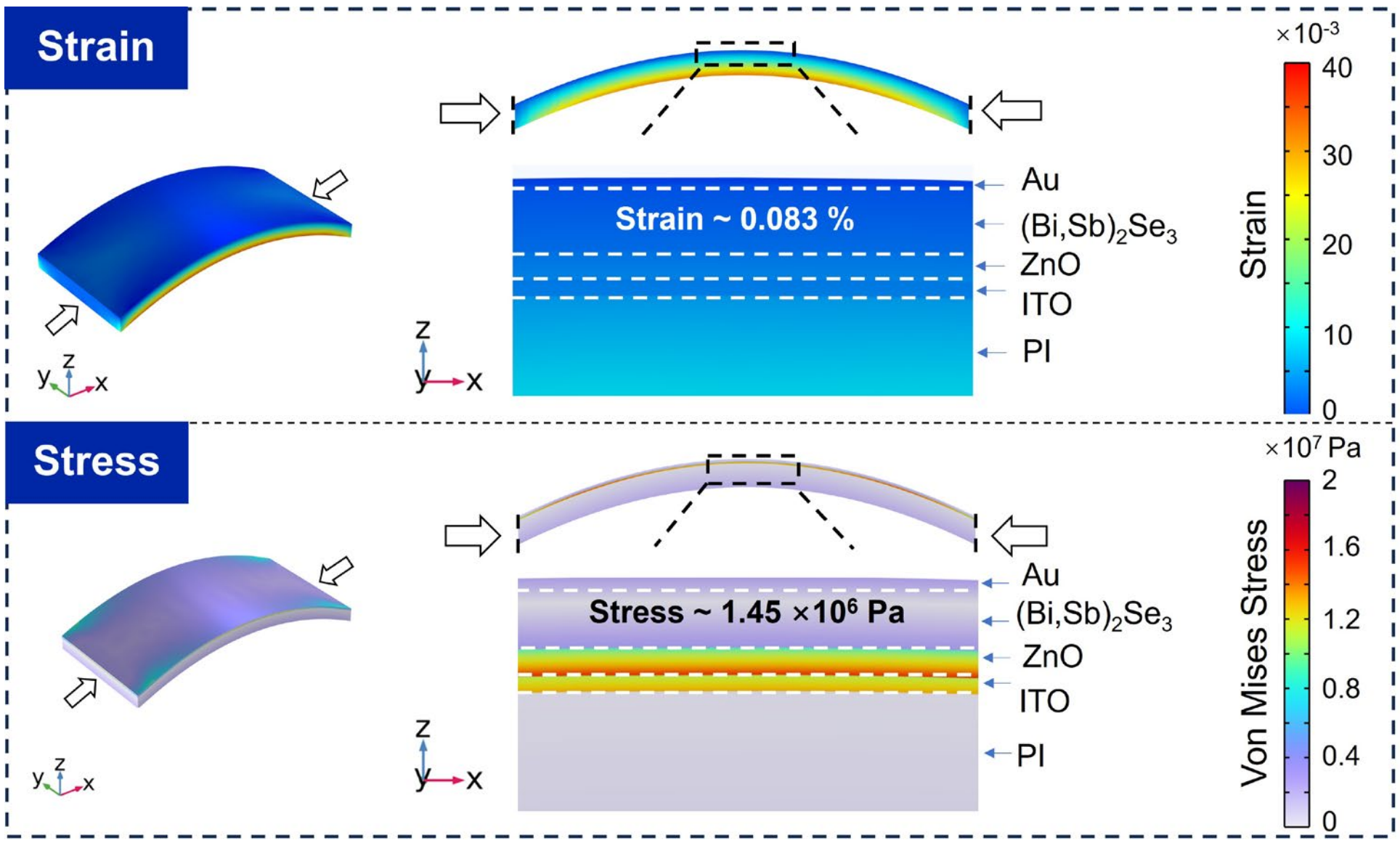

Fig R12. Finite element analysis of the OES under bending, showing strain and von Mises stress distributions across individual functional layers (Au: 0.08 μm; $(Bi,Sb)_2Se_3$: 0.8 μm ; ZnO: 0.34 μm; ITO:0.2 μm; PI: 8 μm).

To address this comment, **Fig 3c** has been replaced by **Fig R12** in the revised manuscript.

Minor-4. Figure 4, for the sensitive audience, please move the actual photo of a mouse and a setup to the supplementary.

Response:

We thank the Reviewer for raising this point. We have moved the photographs of the mouse and the experimental setup from **Fig. 4k** to the Supplementary Information (**Fig. S16**).

Minor-5. In the supplementary section, the fabrication details are not fully provided which can reduce the reproducibility of these devices:

a. Please include a reference here: "The PI substrates with high-temperature resistance and high transmittance were fabricated similarly to the previous report [XX]." How thick is the PI layer?

Response:

We thank the Reviewer for this helpful comment. A reference to the previously reported flexible PI substrate has now been added. The PI film used in this work is consistent with the previous report[5]. The PI substrate was prepared following the method provided by Wuhan Imide New Materials Technology Co., Ltd. In addition, the thickness of the PI layer (~8 μm) has now been specified in the revised Method section.

a. "Subsequently, the ZnO buffer layer was deposited on the PI/ITO." Is this sputtering and what thickness? What thickness is the ITO layer?

Response:

We thank the Reviewer for this helpful comment. The deposition method and thicknesses of both the ZnO buffer layer and ITO layer have been clarified in the revised manuscript. The ZnO buffer layer was deposited by magnetron sputtering with a thickness of approximately 340 nm, and the thickness of the ITO layer was approximately 200 nm.

b. If the method for $(Bi,Sb)_2Se_3$ layer fabrication has been already reported by the team, please include references on your past papers with this material. "The $(Bi,Sb)_2Se_3$ layer···dual-source VTD using Bi_2Se_3 and Sb_2Se_3 powders as the source [XXX]." Important details might be missing for the reproducibility purposes, such as vacuum value during the deposition process and temperature of the substrate (was it water cooled?). From analysis of the references for the main article, it was not possible to locate previous work on using this material system.

Response:

We thank the Reviewer for this constructive comment. The dual-source vapor transport deposition (VTD, Hefei, China, OTF-1200X-II) process used for the $(Bi,Sb)_2Se_3$ layer was previously reported in our earlier work[8,9]. The fabrication conditions followed those reported in that study, with a substrate temperature of 420 °C, a chamber pressure of 2.7 Pa, and air cooling during the deposition process. These details have been specified in the revised Methods section.

c. One of the concerns regarding the fabrication techniques on the polyimide

substrates is the temperature budget of the flexible substrate and induced strain. In our fabrication with polyimide substrates, we've seen the challenges of the strain build-up in the substrates which can lead to the material unintended deformation. Hence, it would be very valuable if the team can comment on those.

Response:

We thank the Reviewer for this comment. The polyimide substrate used in this work was fabricated using an industry-optimized process provided by Wuhan Imide New Materials Technology Co., Ltd., offering improved thermal stability and mechanical robustness. The deposition was conducted within the substrate's thermal tolerance, and no noticeable wrinkling or deformation was observed. Excellent flexibility and stable performance under bending indicate negligible strain buildup.

d. This sentence is quite confusing: "The temperature of Bi_2Se_3 and Sb_2Se_3 was set as 600 °C and 540 °C, respectively, with a heating rate of 20 °C min-1 and "deposition?" duration of 2 min to obtain the target thickness of XXX."

Response:

We thank the Reviewer for pointing out this ambiguity. The sentence has been revised for clarity. The temperatures of Bi_2Se_3 and Sb_2Se_3 were set to 600 °C and 540 °C, respectively, with a heating rate of 20 °C min^{-1}. The deposition time at the target temperatures was 2 min, resulting in a $(Bi,Sb)_2Se_3$ film thickness of approximately 0.8 μm. The revised word has been updated in the Method section.

e. This information might not be sufficient yet again for reproducibility of the fabrication process, it might be beneficial to provide more specific information and the importance of the distance variation for the film quality. "The substrates were placed close to the Sb_2Se_3 source."

Response:

We thank the Reviewer for this comment. The substrates were positioned approximately 6 cm from the Sb_2Se_3 source. This distance is critical for achieving uniform film deposition and high-quality crystallinity, and it has now been clarified in the Methods section to facilitate reproducibility.

f. Is it only gold deposited without any adhesion layer (Ti or Cr)? "The gold electrode was deposited on top of the $(Bi,Sb)_2Se_3$ layer by a resistance evaporation system (Beijing Technol Science)."

Response:

We thank the Reviewer for the comment. Only Au was deposited as the electrode

material without any additional adhesion layer such as Ti or Cr. The gold electrodes were directly deposited on the $(Bi,Sb)_2Se_3$ layer using a resistance evaporation system.

g. Based on the Figure S1 there is also exfoliation and peeling off steps. But those are not describe in the fabrication section.

Response:

We thank the Reviewer for pointing this out. The exfoliation and peeling-off steps shown in **Fig. S1** were not clearly described in the original fabrication section. These steps have now been added to the revised Method section to provide a complete description of the fabrication process.

Reviewer #2

The manuscript "Ultra-flexible optoelectronic stimulator converts tissue-attenuated weak light into electrical signals for cardiac remodeling" by Zhao and colleagues describes the development and validation of a light-driven, flexible stimulator for cardiac therapy. The authors engineer a high-sensitivity photodetector based on bismuth-doped antimony selenide, p-(Bi,Sb)2Se3. This material choice is intended to facilitate the conversion of low-intensity light into electrical impulses delivered to the heart tissue. The study provides comprehensive characterizations, including optoelectronic performance and mechanical flexibility. Furthermore, the authors present both in vitro and in vivo validations to demonstrate the utility in stimulating cardiac cells and tissue. This work represents a promising step toward the development of light-coupled therapies for myocardial infarction regeneration and the realization of wireless, optical pacemakers. While the results are compelling and address a significant need for less invasive cardiac interfaces, the manuscript currently suffers from a lack of detail in the methodology. These omissions hinder a complete interpretation of the data and the reproducibility of the findings. Specific concerns regarding the experimental design and data reporting are detailed below.

Major comments:

Major-1. Device fabrication methods are not sufficiently described, and authors leave a lot of room for interpretation to the reader. Authors must comprehensively describe all the device fabrication methods including the scalable fabrication. Below are some examples.

1a. According to the methods in the SI, the device is fabricated as stacked multilayer, but then the devices in most figures are represented as isolated island. This is confusing.

Response:

We thank the Reviewer for this important comment and apologize for the confusion caused by the schematic illustration. We clarify that the device is fabricated as a continuous multilayer thin-film stack on the flexible substrate. Specifically, ITO/ZnO/$(Bi,Sb)_2Se_3$ layers are deposited sequentially as <u>uniform, laterally continuous films</u> across the entire PI substrate. <u>Only the top Au electrode is patterned</u> (via a shadow mask) into an array to define multiple addressable device cells (pixel-like units) on the same continuous multilayer stack.

In the original schematic (**Fig. 1c**), the array-format Au electrodes were illustrated

to convey the multi-cell architecture, but this could be misinterpreted as if the entire multilayer stack were etched into isolated "islands". We clarify that the multilayer stack remains continuous, and only the top electrode is patterned.

To address this comment, we have revised the figure caption to explicitly state this point.

1b. The authors indicate they fabricate mini-modules, but the method is not clear at all. What type of PVD where use? For instance, in order to fabricate the mini-modules, the gold electrode need to be grown on top of the active area, which is not a concern for any evaporation method; however, the side of the module is about 1.22 μm thick. Ebeam evaporation is not possible to produce such contact metallization requirement.

Response:

We thank the Reviewer for the valuable comments on the description of the mini-module fabrication process. This question is similar to Reviewer 1/Major 6. In response, we have added a schematic of the micro-module fabrication workflow (**Fig R13**), and expanded the fabrication details as follows:

Laser scribing and module fabrication

*$(Bi,Sb)_2Se_3$ mini-modules were fabricated using a standard three-step laser scribing process, following a previously reported procedure with minor modifications[5]. ITO substrates (5 cm × 5 cm) were first patterned by a 1064 nm femtosecond laser (KW-20W-cx, Wuhan, China) to form the P1 lines (10 W, 900 mm s^{-1}), which served as the bottom electrode. A ZnO buffer layer followed by an $(Bi,Sb)_2Se_3$ absorber layer was subsequently deposited onto the patterned substrates. The P2 scribing (10 W, 1300 mm s^{-1}) was then applied to locally remove the $ZnO/(Bi,Sb)_2Se_3$ layers, exposing the underlying ITO and enabling interconnection between adjacent sub-cells. After deposition of the Au electrode, P3 scribing (10 W, 1500 mm s^{-1}) was performed to remove the Au electrode and electrically isolate neighboring sub-cells. The final $(Bi,Sb)_2Se_3$ mini-modules were formed by monolithic series interconnection of the individual sub-cells. The scribing depth was precisely controlled by adjusting the femtosecond laser power and the scan speed. Photograph of the micro-modules and corresponding SEM images of representative regions are shown in **Fig. R14**.*

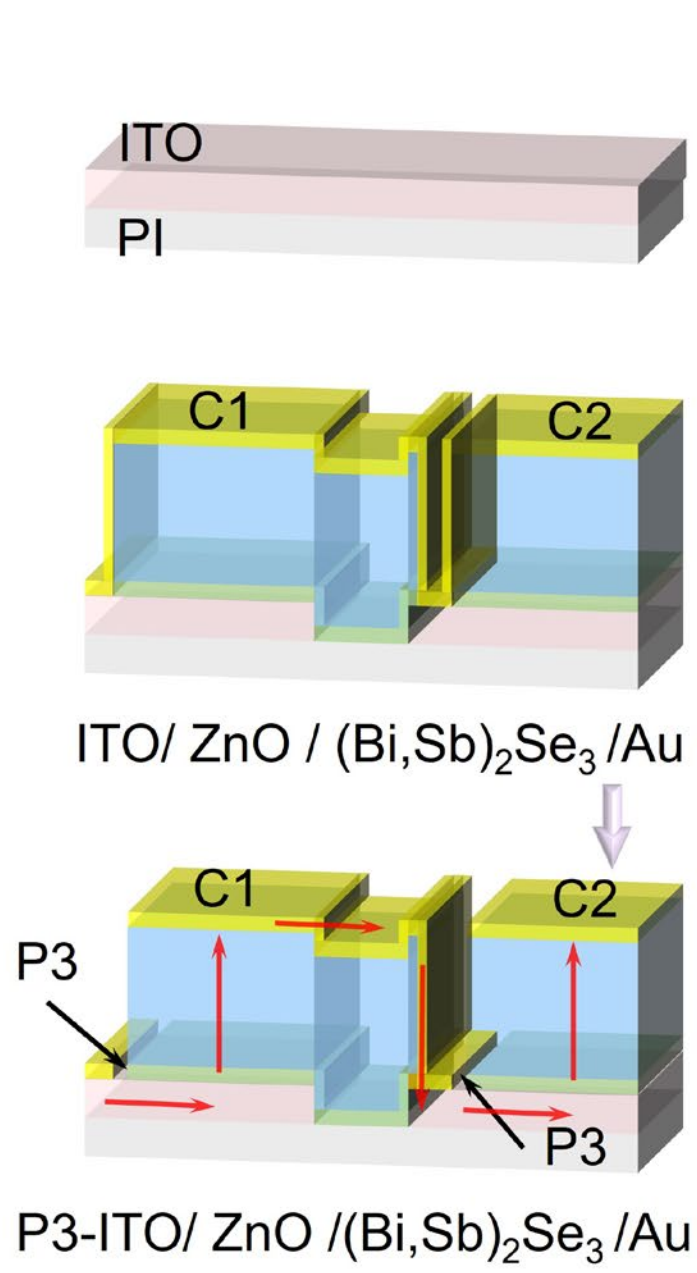

Fig. R13. Schematic of the micro-module fabrication flowchart.

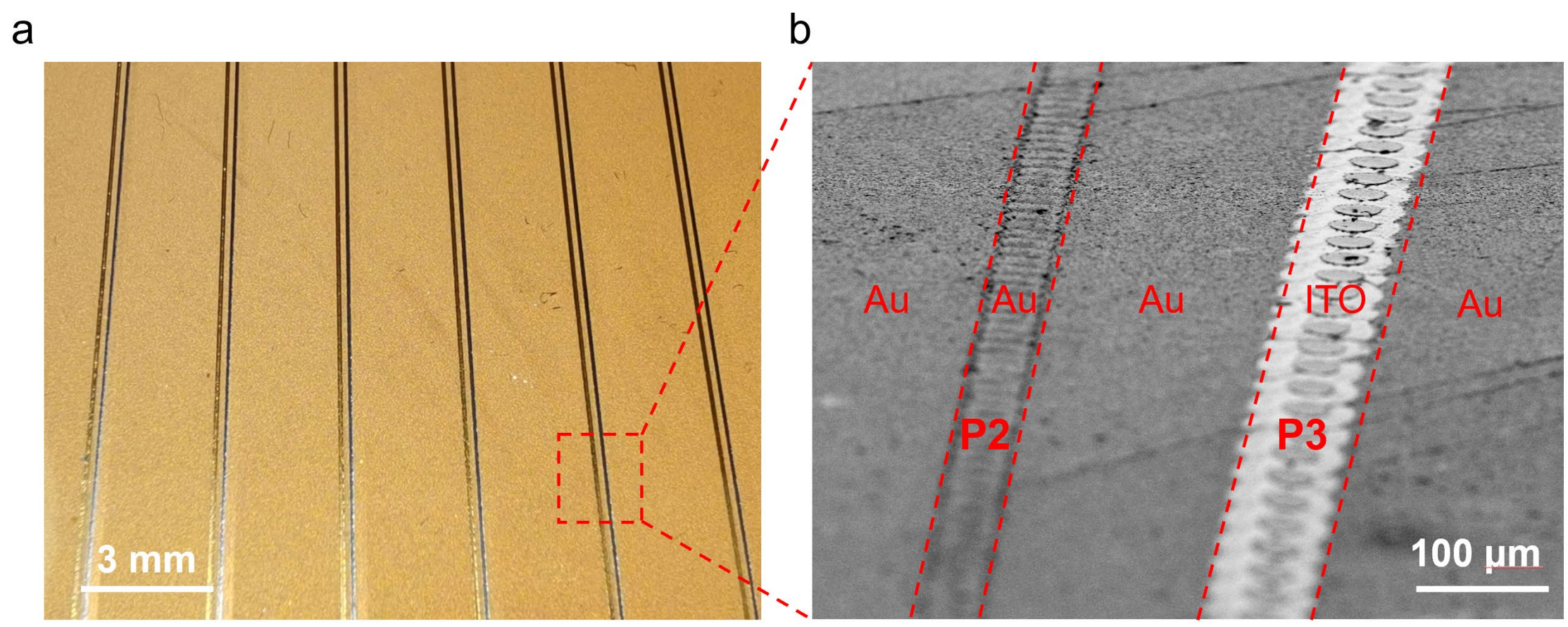

Fig. R14. (a) Photograph of the micro-modules and (b) SEM images of representative regions. The P1 scribe is shallow and is subsequently covered by ZnO/(Bi,Sb)$_2$Se$_3$/Au layers, making it almost invisible in the image.

Secondly, regarding deposition, the mini-modules were fabricated using the same deposition processes as the single-cell devices. Specifically, ITO and ZnO layers were deposited by RF magnetron sputtering (Beijing Technol Science, JCP500), the (Bi,Sb)$_2$Se$_3$ absorber was deposited by dual-source vapor transport deposition (VTD) (OTF-1200X), and the Au top electrode was deposited by resistive thermal evaporation system (Beijing Technol Science, TEMD-600). These fabrication details are now explicitly stated in the revised Experimental section (Section 1. Device fabrication).

Thirdly, the Au electrode can be uniformly deposited on the surface, including regions above the scribed channels, to form a continuous conductive layer. In practice,

the scribed regions exhibit ramped edges (not perfectly vertical), and the Au deposition was performed with substrate rotation, which further improves side of the module coverage over the shallow topography.

To address this comment, **Fig. R13** and **Fig. R14** have been included in the Supplementary Information as **Fig. S6** and **Fig. S7**, respectively. The fabrication details of micro-modules have been added to the Methods section.

1c. The mini-modules are designed and fabricated in a way that they are internally connected in series, such as in Fig 2i. and only two electrodes are needed to perform the wired characterization, is this the case?

Response:

We thank the Reviewer for the careful observation. Yes, the mini-modules are internally connected in series during fabrication, as discussed in fabrication details of micro-modules above. Specifically, adjacent sub-cells are monolithically interconnected through patterned conductive interconnects. Therefore, only two external terminals (located at the two ends of the mini-module) are required for wired electrical characterization. The measured output voltage corresponds to the sum of the photovoltages generated by the serially connected sub-cells.

To address this comment, we have revised the **Fig. 2i** caption to explicitly state that the series interconnection is realized during fabrication and that electrical measurements are performed using the two end terminals.

Major-2. According to the device description the device electrodes produce low and high potential at the ITO and gold, respectively. When stimulating cells, for instance, the two electrodes produce an electric field that drives the cell stimulation. In this case, the geometry of the device points out that this electric field is buried inside the device. In this case, it is not obvious how the device electrode geometry produces electric fields that extend into and stimulates the tissue. In fact, authors do use interdigitated electrodes that are connected to their device to produce different levels of electric field on myocardial cells in their in-vitro validations. However, it was not explicitly described in the text nor the SI methods. It was until I saw Fig S5 that I realized this was the configuration.

Response:

We thank the Reviewer for raising this important point regarding the electric field

distribution and stimulation configuration. We would like to clarify that the electric field generation mechanisms differ slightly between the *in vivo* and *in vitro* experiments, although they are based on the same fundamental principle of potential difference-induced field stimulation.

In vitro (cell studies): The Au and ITO electrodes of the OES were routed to an interdigitated electrode (IDE) pair (**Fig. S12**), such that adjacent fingers carry opposite potentials. This produces a well-defined lateral electric field in the culture medium, enabling controlled studies of field-dependent cardiomyocyte responses.

In vivo (animal studies): The OES photovoltage between Au and ITO electrodes drives stimulation through electrodes interfacing with tissue, where the potential drop occurs in the conductive tissue and electrolyte near the exposed electrode surfaces.

To address this comment, we have added the Methods section with a detailed description of the interdigitated electrode stimulation, as below:

In Vitro Electric Field Stimulation Using Interdigitated Electrodes

For in vitro stimulation, the positive and negative terminals of the mini-modules were connected to an interdigitated electrode (IDE) array composed of parallel gold fingers (width: 100 μm; spacing: 300 μm). The electric field strength was approximated as E=V/d, where V is the applied potential difference and d is the inter-finger spacing. Field magnitude was tuned by adjusting the output voltage and selecting different electrode contact pairs. Stimulation frequency was controlled by illuminating the mini-modules with pulsed light at programmable frequencies, generating electrical outputs at corresponding frequencies. Neonatal rat cardiomyocytes (NRCMs) were seeded on the IDE surface and stimulated with defined field strengths and frequencies.

Major-3. In contrast with the in-vitro experiment where the OES device works as energy harvester and connected to the interdigitated electrodes to produce the stimulation to cells, the OES device interfaces directly with the heart it is the gold electrodes that produce the stimulation. In this case, the in-vitro validation is not relevant to indicate the OES produces electric field to tissue in vi-vivo because the conditions are very different.

Response:

We thank the Reviewer for this important point and agree that the electrode geometry and the surrounding medium differ between the in-vitro and in-vivo experiments. We designed the in-vitro interdigitated-electrode (IDE) configuration was

to provide a well-defined and controllable electric-field environment for parametric evaluation of cardiomyocyte responses (**Fig. S12**), which would be difficult to quantify directly on beating tissue. Importantly, although the electrode shape differ, the fundamental stimulation principle is the same: illumination generates a photovoltage between the electrodes (Au at higher potential and ITO at lower potential), and stimulation arises from the potential drop in the conductive biological medium (culture electrolyte or cardiac tissue).

To demonstrate that the OES produces effective bioelectric stimuli under the direct heart-interface condition, we rely on our in-situ validations: (i) Photoelectric stimulation via OES effectively promotes myocardial remodeling post-infarction. (**Fig. 5**); (ii) in the swine model we directly recorded the output voltage on the heart surface and observed rhythm modulation, with heart rate increasing from 110 to 130 bpm upon photoelectric stimulation (**Fig. 6i**). These in-vivo measurements confirm that, despite tissue impedance-related attenuation (discussed via an RC model), the photovoltage generated by the OES is effectively delivered to cardiac tissue and is sufficient for functional stimulation.

Major-4. Although the results are exciting, this Reviewer lost enthusiasm when realizing there is not a clear discussion or consideration how the device would be capable to operate robustly in-vivo when considering biological conditions. For instance, devices suffer from fouling due to biofluids around it and scar tissue formation, which is common in implantable devices. Here, the device is not encapsulated by any means, which seems a good thing at first, but is this relevant for demonstrating a translatable technology of this nature?

Response:

We thank the Reviewer for this important comment. This question is similar to Reviewer 1/Major 14 and Reviewer 3/Major 4. To evaluate device robustness under biological conditions, we performed post-implantation morphology and photoresponse characterization of the OES following both cardiac and subcutaneous implantation.

After cardiac implantation after 4 weeks, optical images show that the OES was encapsulated by a thin fibrotic tissue layer on the heart surface, while remaining structurally intact after removal of the surrounding tissue (**Fig. R15a**). SEM images of the explanted device show only minor surface cracks in the $(Bi,Sb)_2Se_3$ layers, likely associated with chronic cyclic deformation imposed by cardiac motion, while no

obvious large-area delamination of the active layer was observed (**Fig. R15c**). In addiation, the photocurrent of the device showed a decrease of 8.9% compared to the pre-implantation value (**Fig. R15b**). These results demonstrate that the OES maintains substantial structural integrity and photoelectric functionality after 4 weeks of direct implantation, despite the absence of encapsulation.

In addiation, OES devices were implanted subcutaneously and retrieved at 15, 30, and 45 days. The devices remained intact at all time points (**Fig. R15e**), and although the normalized photocurrent gradually decreased, a stable photoresponse was preserved (**Fig. R15f, g**). After 45 days, the photocurrent decreased by 13.7% compared to the initial value.

To avoid overstatement, we have also clarified in the revised figure caption that these results support mid-term *in vivo* stability. Longer-term implantation will still require effective encapsulation strategies to prevent degradation caused by prolonged mechanical friction and exposure to biofluids.

To address this comment, **Fig. R15** has been added as **Fig. S21**, and the description of device robustness under biological conditions has been added in the revised manuscript (Page 8-9, highlight in blue).

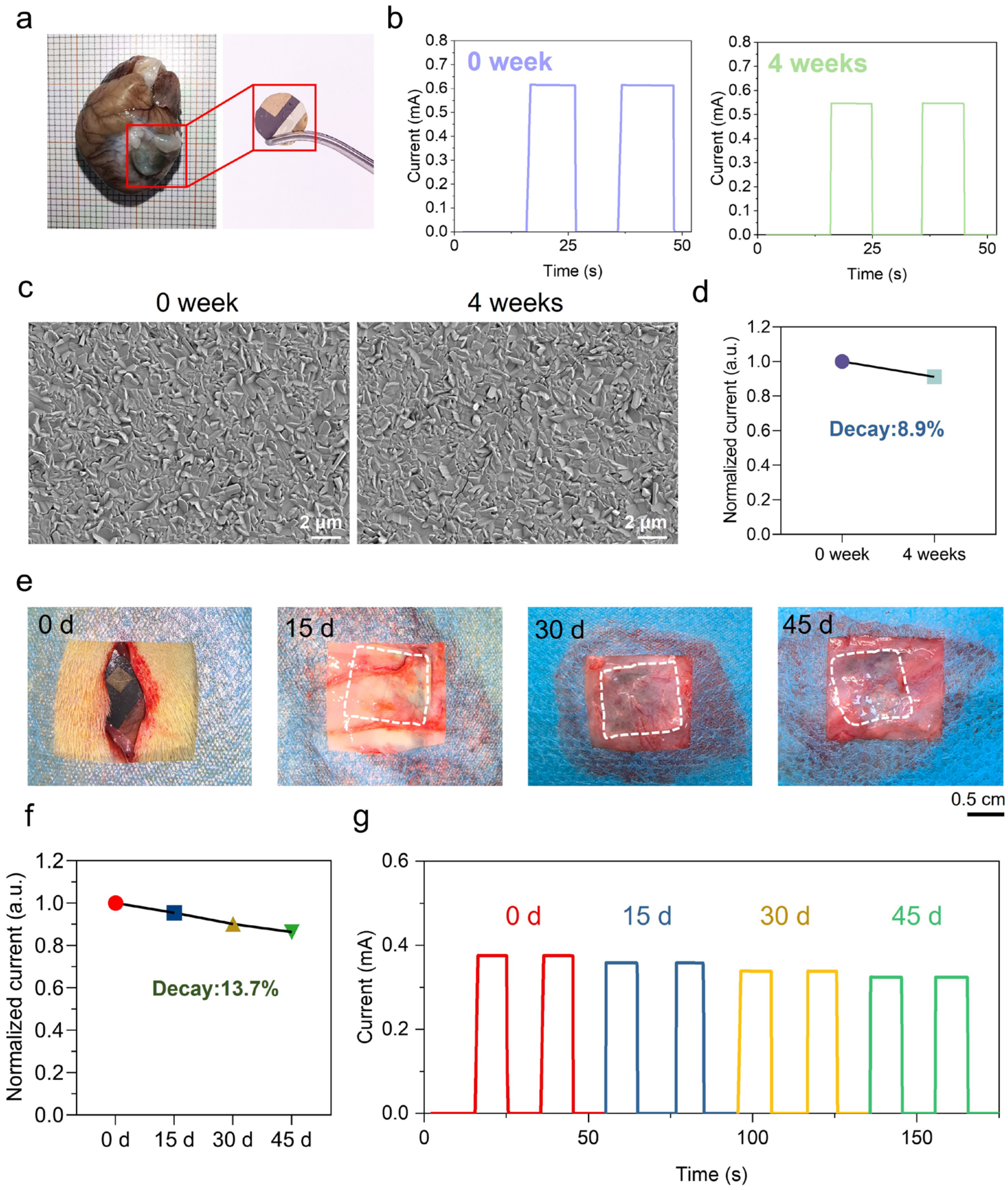

Fig. R15. Optical images of the OES retrieved after 4 weeks of implantation on the SD rat heart. (b) *I-T* curves recorded before and after implantation. (c) SEM images of the $(Bi,Sb)_2Se_3$ layers before implantation and retrieved after 4 weeks, indicating minor surface cracks without obvious delamination. (d) Photocurrent response of the OES after retrieval following 4 weeks of implantation. (e) Optical images of OES after subcutaneous implantation for 0, 15, 30, and 45 days. (f, g) Normalized photocurrent and *I-T* curves versus subcutaneous implantation time, under a illumination of 24.7 mW cm^{-2} at 780 nm.

Minor comments:

Minor-1. It was not clear what is the direction of the device. On one side there is 80 nm of gold, whereas on the other side there is 8 or 40 μm of PI substrate. I assume the gold side in on contact with the tissue. This way, light impinging from the PI substrate will be attenuated the less before light reaching the active area. If this is the case, authors must clearly describe the operation.

Response:

We thank the Reviewer for pointing out the need to clarify the device orientation and operating configuration. The Reviewer's assumption is correct. In our experiments, the 80 nm Au electrode side faces and contacts the tissue, serving as the stimulation interface, whereas the 8 μm PI substrate side faces outward. Illumination is applied from the PI side (back-illumination) so that light passes through the thin PI/ITO side with minimal attenuation and reaches the photoactive junction efficiently, while avoiding optical blocking by the opaque Au layer.

To address this comment, we have explicitly indicated the direction of the device in the revised main text (Page 7, highlight in red): "During operation, OES devices with an 8 μm-thick PI substrate and a diameter of 5 mm were placed directly into the infarct zone, with the Au electrode facing the tissue and illuminated from the PI side (back-illumination) to avoid shading by the Au electrode".

Minor-2. Following up on the previous comment. It is not explicitly written what is the thickness of the PI used for the devices. It is mentioned that there was improvement in stress and strain when reducing the PI film thickness, but not what the final thickness was for the devices. In fact, authors omit key technical information like total thickness of the device, the area of the devices, etc.

Response:

We thank the Reviewer for pointing out the omission of key technical parameters. The final PI thickness used in the devices is 8 μm. This question is similar to Reviewer 1/Major 8. This thickness is selected to balance mechanical compliance and handling robustness, as discussed in the mechanical characterization section. The total thickness of the device, including the PI substrate, ITO layer (0.2 μm), ZnO layer (0.34 μm), photoactive layers (0.8 μm), and gold electrode (0.08 μm), is approximately 9.42 μm. For the *in vivo* experiments, the devices were fabricated in a circular geometry (diameter=5 mm), corresponding to an area of ~19.6 mm^2, to cover the targeted cardiac

region while maintaining conformal contact.

To address this comment, we have added more details of device information in the in the revised manuscript (Page 7, highlight in red).

Minor-3. Authors demonstrate that 780 nm light penetrates through freshly excise skin and fat, which is expected considering the EQE reported and the low optical absorption of tissue in the near infrared band. However, how much is the thickness of this tissue? In addition, the heart is protected by the ribcage, such as in humans. The authors do not discuss, and support with experiment, how thick the tissue should be to replicate the intended tissue thickness of the animal models, including humas. In addition, the effect of the ribcage bone and cartilage is not mentioned nor studied at all nowhere in the text or SI.

Response:

In our *in vitro* experiments, we evaluated 780 nm light penetration through well-defined tissue thicknesses, including 2 mm skin, 2 mm fat, and a combined 4 mm skin-fat stack. We agree that in adult humans the skin-to-heart distance can reach ~30 mm[10], which is significantly greater than the thickness tested here. Importantly, in addition to the in-vitro measurements, we performed *in vivo* closed-chest experiments in Sprague-Dawley rat, although the thoracic path length to the heart is shorter than in humans. As shown in **Fig. 4l**, externally applied 780 nm illumination reached the implanted device. This closed-chest configuration inherently includes attenuation by the skin, muscle, rib bone, and costal cartilage, providing a proof-of-concept whole thorax validation in a small animal model.

We acknowledge that adult humans have much larger skin-to-heart distances, and the ribcage (bone and cartilage) can introduce additional attenuation. Translation to thicker human tissues would likely require optimized illumination strategies (e.g., intercostal optical windows, minimally invasive light delivery, wavelength/irradiance optimization within established safety constraints).

To address this comment, the illumination was positioned to minimize rib obstruction, which has been clarified in the revised manuscript (Page 9, highlight in green). We also added a discussion acknowledging the limitations for translation to thicker human tissues, which will be addressed in future work.

Minor-4. The viability experiment that uses Sb_2Se_3 co-cultured with H9C2 cells and NRCMs did not include antimony. What is the rationale of excluding antimony considering its toxicity?

Response:

We thank the Reviewer for raising this important safety-related question. The toxicity of antimony is mainly associated with its soluble ionic forms. The biological toxicity of Sb mainly comes from its soluble Sb compounds (e.g., Sb (III) salts). In this study, Sb is incorporated in a crystalline chalcogenide lattice $(Bi,Sb)_2Se_3$, which is chemically stable and exhibits extremely low solubility under physiological conditions[11]. Therefore, free Sb ions are not expected to be released during co-culture. Importantly, our broader biocompatibility assessment shows no detectable cytotoxicity, hemolysis, or inflammatory response for the OES and its Sb-containing photoactive component (**Figs. S17** and **S18**).

To address this comment, we have explicitly stated in the caption of **Fig. S17** that Sb is present in the chemically stable solid $(Bi,Sb)_2Se_3$ phase.

Minor-5. Based on required implantation conditions for a device of this nature (long implantation), these devices will remain in contact with moisture and under body temperature conditions. These two important parameters were not included in any in-vitro experiments, thus there is not a prediction of how long these devices could operate in-vivo. For example, what is the performance of the device when it remains under biological conditions for long time, say at 37C under saline buffer?

Response:

We thank the Reviewer for this important comment. We have already characterized the stability of the device after *in vivo* implantation in our response to Major Comment #4 above. Here, we further performed an *in vitro* stability test by immersing the OES in physiological saline at 37 °C for 45 days to simulate the biofluid environment. As shown in **Fig. 16b**, after 45 days of immersion, the all-inorganic OES exhibited negligible morphological changes during immersion, owing to the intrinsic physicochemical stability. The photocurrent under 530 nm and 780 nm illumination decreased by only 3.7% and 4.5%, respectively, (**Fig. 16c**). Expectably, the performance degradation observed in this *in vitro* test was smaller than that measured after *in vivo* implantation.

To address this comment, **Fig. R16** and the related discussion have been added as

Fig. S19 in the revised manuscript (Page 7, highlight in blue).

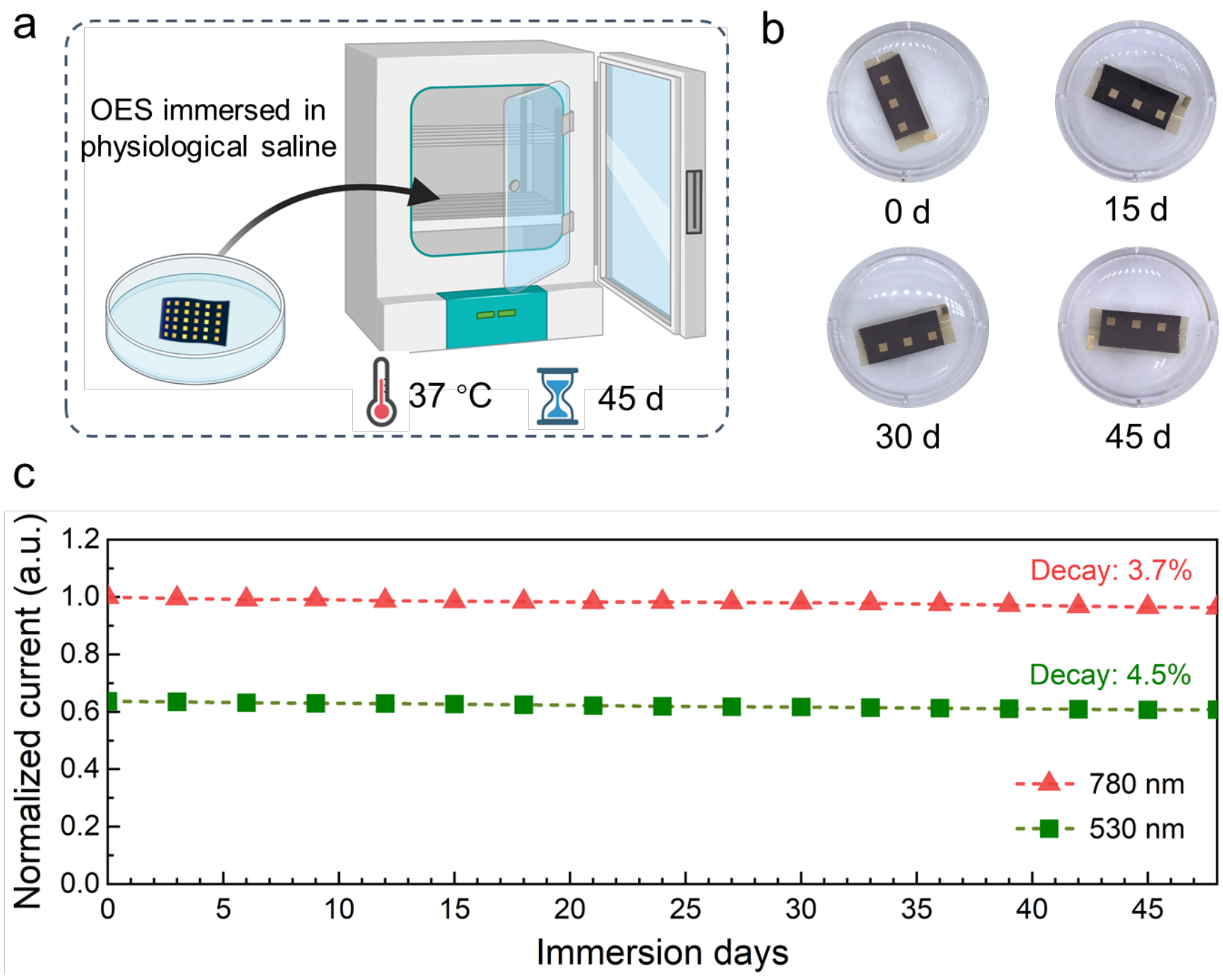

Fig. R16. (a) Schematic of the OES immersed in physiological saline at 37 °C for 45 days. (b) Photographs of the OES after 0, 15, 30, and 45 days of immersion, showing nearly no observable change in appearance. (c) Photocurrent of the OES under 530 nm and 780 nm illumination under a light intensity of 24.7 mW cm^{-2}, with only 3.7% and 4.5% decreases after 45 days, respectively.

Minor-6. The rat myocardial infarction model, according to the manuscript, uses 1hr of optical stimulation every week. Authors report that the Light-OES group demonstrated robust ventricular contraction and preserved wall motion at week four from implantation. Is one hour a week, 4 hours in that period, enough to achieve the results? Following on my major concern 1 above the descriptions of the in-vivo experiments should be more detailed, perhaps a chronological graph that indicates implantation, stimulation, validations, etc., which is typical in these types of animal experiments.

Response:

We sincerely thank the Reviewer for this insightful comment regarding the stimulation regimen and the clarity of the *in vivo* experimental design. Considering that prolonged continuous stimulation may cause phototoxicity or thermal effects, here we intentionally control the stimulation time to only 1 hour each time (at 1 Hz, each 1 hour

session delivers 3,600 stimulation cycles). The total stimulation time seems to be only 4 weeks. However, our approach was designed as a periodic modulatory intervention rather than continuous pacing. The aim was to repeatedly activate electrophysiological involved in post-infarction remodeling.

Previous studies have shown that intermittent stimulation can induce sustained cardiac effects by modulating calcium handling and remodeling-related signaling pathways[12,13]. Importantly, the week-4 outcomes show consistent improvements across multiple independent endpoints, supporting that repeated weekly stimulation is sufficient to induce sustained therapeutic effects (**Fig 4**): (a) reduced fibrosis, (b) significantly increased Cx43 expression, (c) enhanced arteriole formation, and (d) nerve fiber regeneration observed only in the Light-OES group. Functionally, echocardiography at 4 weeks post-implantation showed robust ventricular contraction and preserved wall motion in the Light-OES group, with EF = 59.5% and FS = 33.1%. These convergent structural, molecular, and functional readouts collectively support the efficacy of the periodic stimulation regimen.

To address this comment, the original schematic (**Fig. 5b**) has been revised so that the experimental timeline and procedures of the *in vivo* study are presented more clearly.

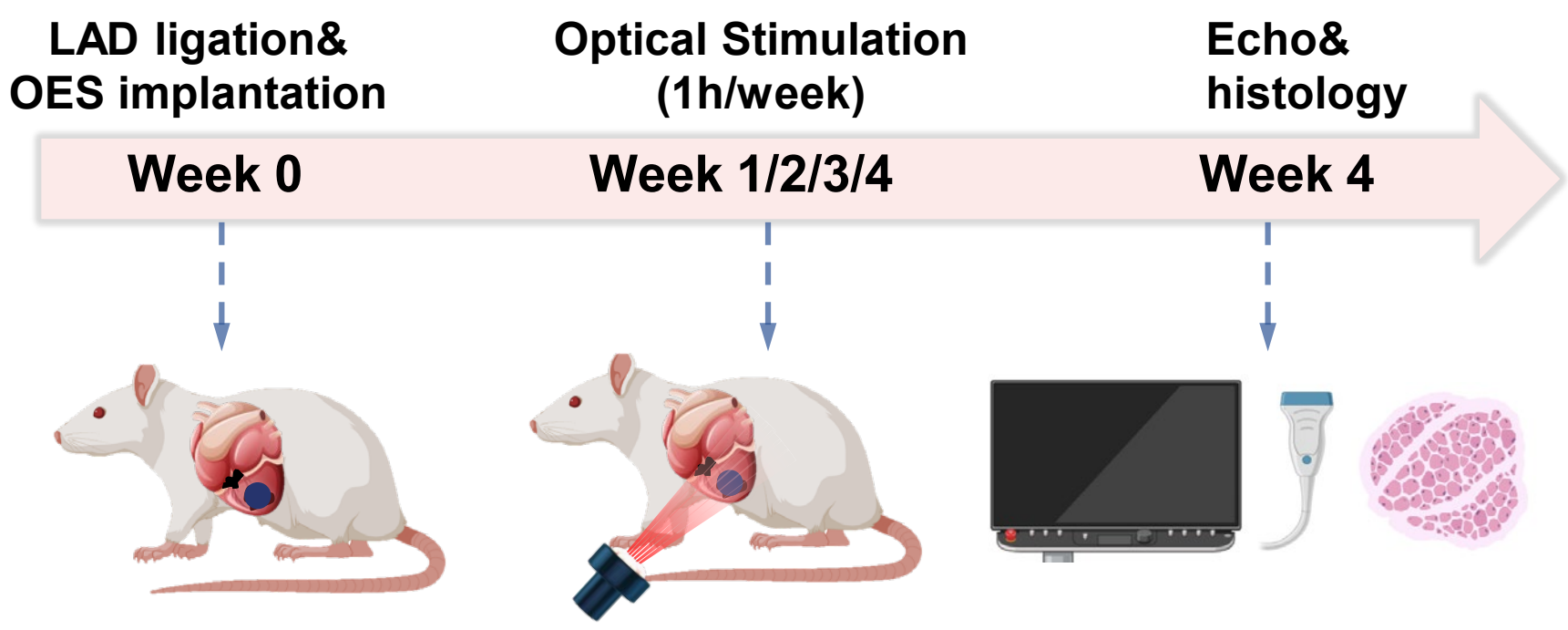

Fig. R17. Myocardial infarction (MI) was induced in rats via left anterior descending (LAD) artery ligation. OES was implanted at the infarct site, and the therapeutic effects of photoelectric stimulation (780 nm) were assessed 4 weeks post-implantation using echocardiography and histology.

Reviewer #3

Your manuscript presents an innovative and well-executed study demonstrating an ultraflexible optoelectronic stimulator (OES) based on $(Bi,Sb)_2Se_3$ for weak-light–driven modulation of cardiac tissue. The work successfully integrates material-level flexibility, high-efficiency photoelectric conversion under near-infrared illumination, and in vivo demonstration in both rat and swine models. The results provide compelling evidence that the proposed OES platform can restore myocardial conduction and improve cardiac function under low-power stimulation, while maintaining performance across scalable device geometries. This combination of weak-light sensitivity, cardiac applicability, and scalability will be of substantial interest to the bioelectronics and cardiovascular engineering communities. While the study is strong and highly promising, several aspects require clarification and improvement to fully support the translational narrative, particularly regarding device uniformity, mechanical durability, and long-term biostability. If the authors successfully address the major and minor comments outlined below, I believe the manuscript would be suitable for publication in Nature Communications, as it presents a meaningful bioelectronic platform with clear clinical relevance and technological novelty. I therefore recommend addressing the following points for revision.

Major comments:

Major-1. In Figure 1, the current explanation appears insufficient to convey the overall research objective and the operating principle of the device. Since Figure 1 serves as a conceptual schematic introducing the study, more detailed descriptions for each caption would be better. I suggest clearly describing the functional significance of each panel and what advantage it represents.

Response:

We thank the Reviewer for this insightful comment. We have optimized Fig. 1 and carefully revised the caption to provide a more comprehensive conceptual description. The updated caption now (i) explicitly introduces the therapeutic objective of the optoelectronic stimulator, (ii) clarifies the light-to-electricity operating mechanism, and (iii) explains the functional significance and corresponding advantages of each panel, including therapeutic selectivity, mechanical flexibility, and efficient weak-light energy conversion. These revisions improve the clarity of the conceptual schematic and better highlight the design rationale of the proposed device.

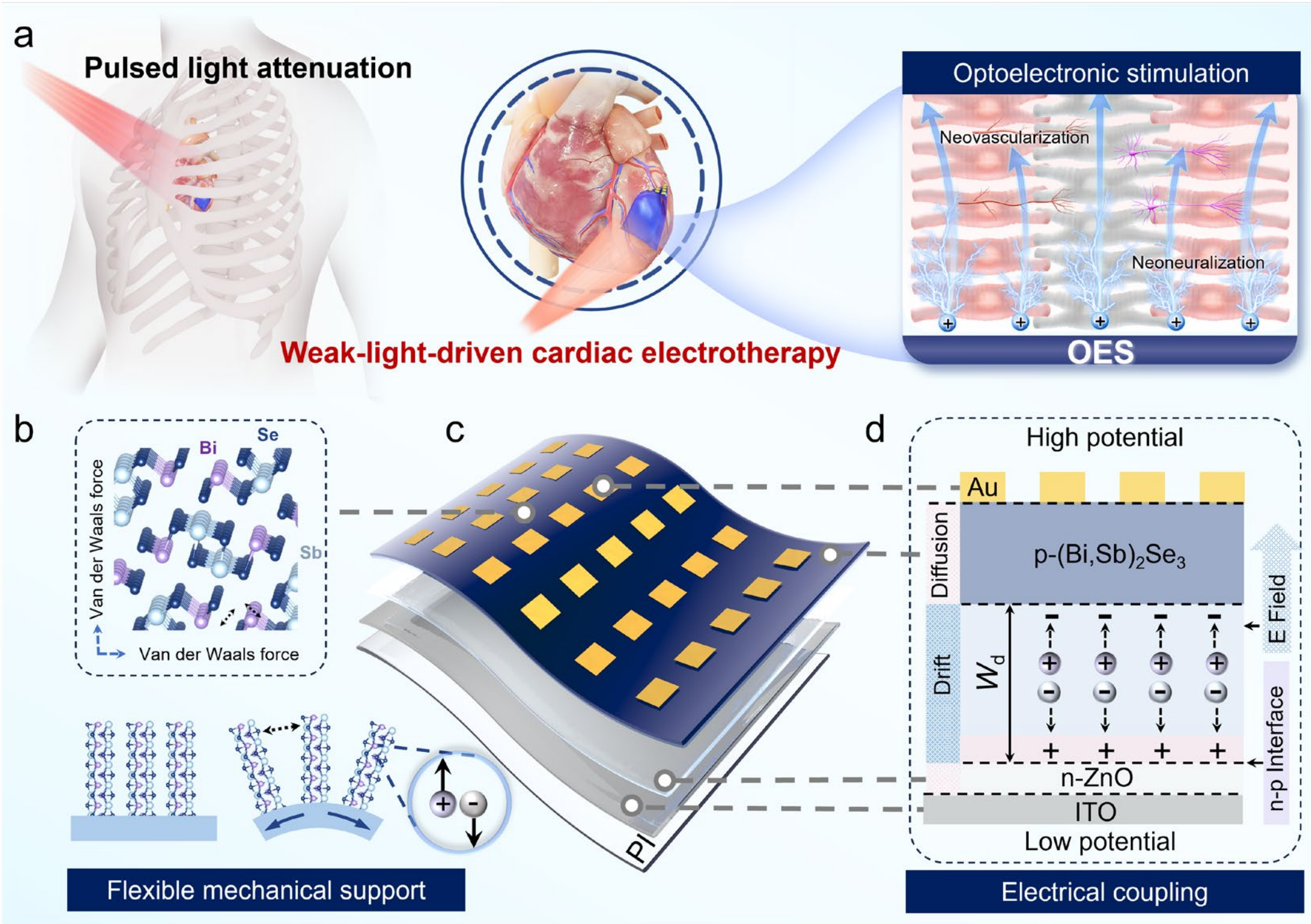

Fig. R18. Design concept and operating principle of the optoelectronic stimulator (OES). The OES is designed to achieve safe and effective deep-tissue optoelectronic stimulation under weak light illumination. **(a)** Schematic of the therapeutic concept and working mechanism. Pulsed light penetrates biological tissues and attenuates to weak intensity, yet remains sufficient to activate the OES. The device converts incident light into localized electrical stimulation through photovoltaic charge generation and separation, thereby promoting neurogenesis, neovascularization, and cardiomyocyte remodeling at infarction sites while avoiding phototoxicity to healthy cardiomyocytes. **(b)** Schematic of the mechanical adaptability of the OES. Owing to its one-dimensional (1D) inorganic semiconductor structure and weak interlayer molecular van der Waals interactions, the device can conformally integrate with dynamically beating cardiac tissues, thereby improving stimulation stability. **(c, d)** Structural design and working principle of the OES. The device consists of a flexible polyimide (PI) substrate, layered with infrared-transparent indium tin oxide (ITO), n-type zinc oxide (n-ZnO), a photovoltaic p-$(Bi,Sb)_2Se_3$ layer, and Au electrodes. This heterostructure, together with the built-in electric field, facilitates efficient photocarrier generation and separation, enabling effective light-to-electricity conversion even under weak illumination, which is critical for deep-tissue applications.

To address this comment, **Fig. 1** has been replaced by **Fig. R18** in the revised manuscript.

Major-2. In Figure 2a, b, although it is stated that photocurrent mapping was performed under illumination from multiple orientations, the methodological details remain unclear. Specifically, it is not clear what pixel size or device area was mapped, nor by what measurement approach the mapping was conducted. With the current figure alone, it is difficult to interpret the spatial resolution or how the mapped distribution corresponds to the device structure. Therefore, additional clarification or figure improvement appears necessary.

Response:

We thank the Reviewer for this valuable suggestion. This question is similar to Reviewer 1/Major 2. The experimental details have been clarified in the revised manuscript. Specifically, a 780 nm LED (intensity: 24.7mW cm^{-2}) was focused by a convex lens to form a localized spot on a device with a 3×3 electrode array. The light source was positioned 4 cm above the device. The focused light spot was controlled to scan across different positions on the array, and at each position the photocurrent signals from all nine electrodes were recorded simultaneously. The currents were monitored using a semiconductor device parameter analyzer (Agilent B1500A) in a shielded enclosure.

The acquired current data were subsequently subjected to smoothing to enhance spatial continuity, and the processed data were used to construct spatially resolved photocurrent 2D mapping, enabling visualization of the localized illumination position. The nine mappings shown in **Fig. 2b** represent the different illumination positions on the 3×3 electrode array, in sequential order from left-to-right and from top-to-bottom. The mapping results demonstrate that the fabricated device possesses precise spatial addressability, allowing site-specific photoelectric stimulation as required.

To address this comment, we added a scale bar to **Fig. 2b**. We also expanded the description and experimental detail for **Fig. 2a, b** in the Methods section and its revised figure caption.

Major-3. The mechanical bending test appears to have been conducted considering the curvature of the cardiac environment. However, 1000 bending cycles seem insufficient to evaluate reliability under an implantable cardiac setting. Since the heart undergoes

continuous contraction- relaxation cycles, a minimum of approximately 10,000 bending cycles would be required to substantiate claims regarding mechanical durability.

Response:

We thank the Reviewer for this insightful comment. This question is similar to Reviewer 1/Major 10. To evaluate the potential delamination and crack propagation after repeated bending, the flexible OES was subjected to 10,000 bending cycles under a minimum bending radius of 1 mm, as shown in **Fig. R19a**. This condition is substantially more severe than the deformation encountered under normal operating conditions. In situ SEM observation in the bent state revealed no obvious cracks on the surface of the $(Bi,Sb)_2Se_3$ layer (**Fig. R19b**). In addition, SEM images taken after 0, 100, 1,000, and 10,000 bending cycles show that slight surface microcracks appear after 1,000 cycles, while no obvious delamination of the active layer was observed even after 10,000 cycles (**Fig. R19c**).

Despite the appearance of limited surface microcracks after extensive cycling, the optoelectronic output remains largely preserved, decreasing by only 2.8% after 10,000 bending cycles (**Fig. R19d**), and the time-resolved photocurrent response under repeated light on/off cycles remains stable (**Fig. R19e**). These results indicate that the OES maintains good interfacial integrity and crack-tolerant mechanical performance under extreme bending conditions.

This mechanical compliance of $(Bi,Sb)_2Se_3$ is related to its quasi-one-dimensional crystal structure, featuring strong covalent bonding within the $(Sb_4Se_6)_n$ ribbons/chains and weak van der Waals interactions between adjacent ribbons/chains, which gives rise to pronounced structural anisotropy[6,7].

To address this comment, **Fig. R19** has been added as **Fig. S10.** The relevant discussion of mechanical resilience has been added to the revised manuscript (Page 5, highlight in blue), and the corresponding references have been added as Refs. [31,39].

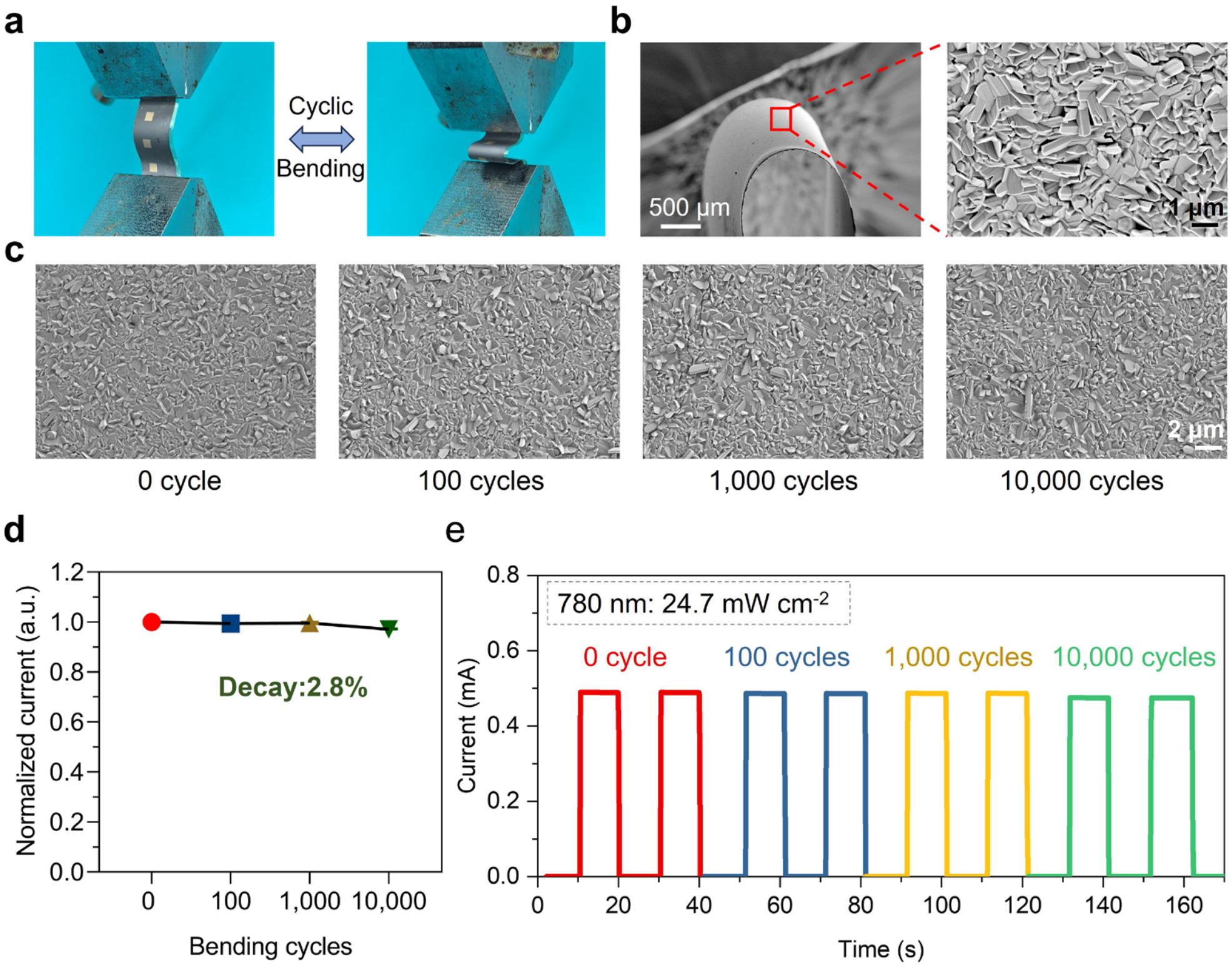

Fig. R19. (a) Photographs of the cyclic bending test. (b) In situ and higher-magnification surface SEM images of the OES in the bent state at a bending radius of 1 mm. (c) SEM images of the OES after 0, 100, 1,000, and 10,000 bending cycles. (d) Normalized photocurrent of the OES measured after 0, 100, 1,000, and 10,000 bending cycles. (e) Time-resolved photocurrent (*I-T*) response of the OES under repeated light on/off cycles (780 nm, 24.7 mW^{-2}).

Major-4. This study includes biocompatibility assessments, but data regarding the biostability of the device and its operational lifetime in an implant environment are not presented. For implantable bioelectronic devices, it is important to demonstrate how long the device can maintain functionality under physiological conditions. Therefore, the lifetime of device assessment would be necessary.

Response:

We thank the Reviewer for this valuable comment. This question is similar to Reviewer 1/Major 14 and Reviewer 2/Major 4. To evaluate the biostability and operational lifetime of the device under physiological conditions, we performed post-implantation morphology and photoresponse characterization of the OES following both cardiac and subcutaneous implantation.

After cardiac implantation after 4 weeks, optical images show that the OES was encapsulated by a thin fibrotic tissue layer on the heart surface, while remaining structurally intact after removal of the surrounding tissue (**Fig. R20a**). SEM images of the explanted device show only minor surface cracks in the $(Bi,Sb)_2Se_3$ layers, likely associated with chronic cyclic deformation imposed by cardiac motion, while no obvious large-area delamination of the active layer was observed (**Fig. R20c**). In addition, the photocurrent of the device showed a decrease of 8.9% compared to the pre-implantation value (**Fig. R20b**). These results demonstrate that the OES maintains substantial structural integrity and photoelectric functionality after 4 weeks of direct implantation, despite the absence of encapsulation.

In addition, OES devices were implanted subcutaneously and retrieved at 15, 30, and 45 days. The devices remained intact at all time points (**Fig. R20e**), and although the normalized photocurrent gradually decreased, a stable photoresponse was preserved (**Fig. R20f,g**). After 45 days, the photocurrent decreased by 13.7% compared to the initial value.

To avoid overstatement, we have also clarified in the revised figure caption that these results support mid-term *in vivo* stability. Longer-term implantation will still require effective encapsulation strategies to prevent degradation caused by prolonged mechanical friction and exposure to biofluids.

To address this comment, **Fig. R20** has been added as **Fig. S21**, and the description of device robustness under biological conditions has been added in the revised manuscript (Page 8-9, highlight in blue).

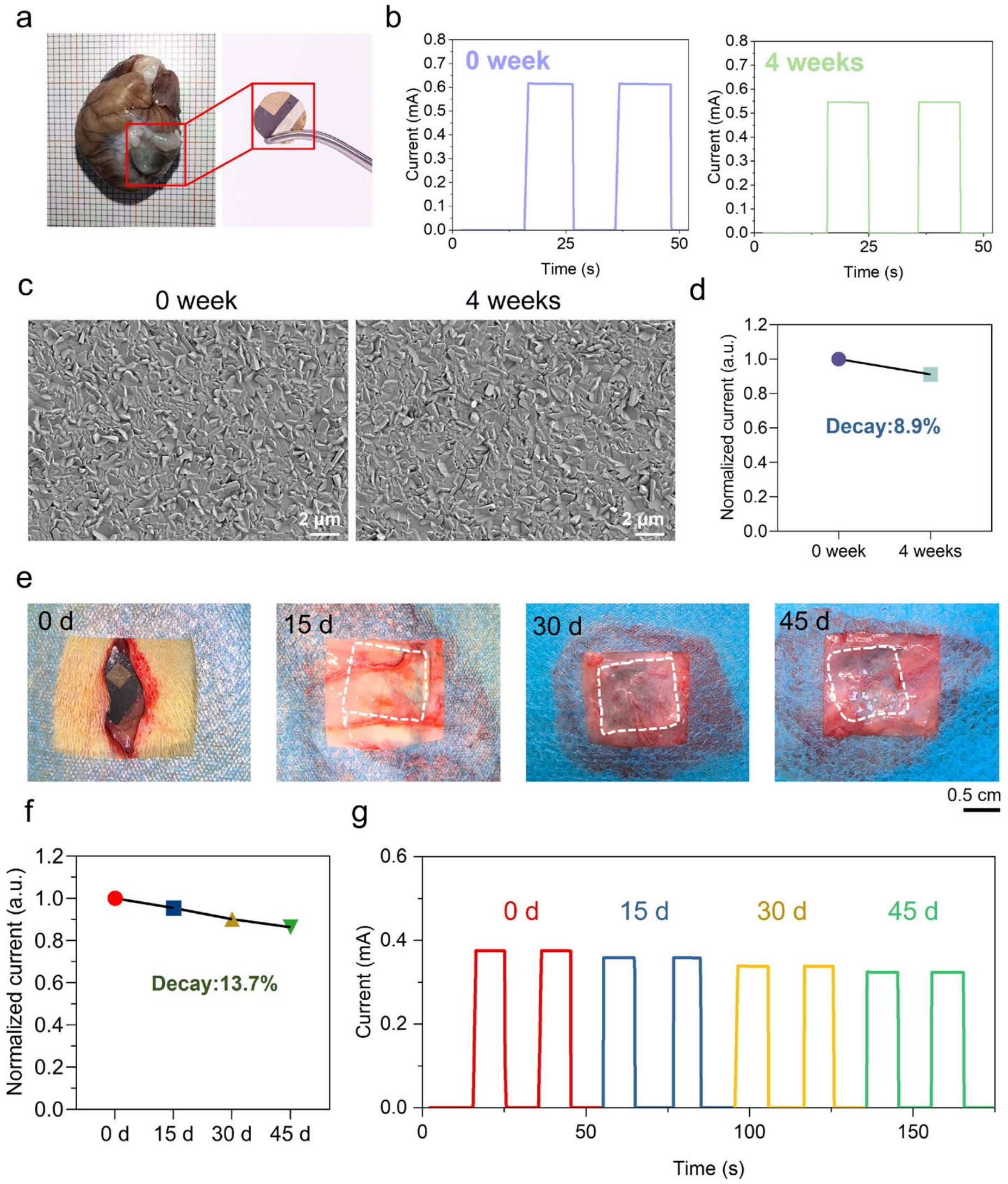

Fig R20. (a) Optical images of the OES retrieved after 4 weeks of implantation on the SD rat heart. (b) *I*-*T* curves recorded before and after implantation. (c) SEM images of the $(Bi,Sb)_2Se_3$ layers before implantation and retrieved after 4 weeks, indicating minor surface cracks without obvious delamination. (d) Photocurrent response of the OES after retrieval following 4 weeks of implantation. (e) Optical images of OES after subcutaneous implantation for 0, 15, 30, and 45 days. (f, g) Normalized photocurrent and *I*-*T* curves versus subcutaneous implantation time, under an illumination of 24.7 mW cm^{-2} at 780 nm.

Major-5. This research presents data showing that the electrical output varies with optical stimulation intensity. However, there are no data demonstrating whether uniform output is maintained across individual device cells. For expandable platforms such as this one, spatial uniformity is an important characteristic, and in applications involving multi-site stimulation or large-area operation, cell-to-cell variation could significantly influence stimulation outputs. Therefore, providing comparative photocurrent or voltage output data across individual cells would support the reliability and scalability of the device.

Response:

We thank the Reviewer for this valuable comment regarding the spatial uniformity of the device output. In the revised manuscript, we have now evaluated the electrical outputs from multiple individual cells (Au electrode) under identical optical stimulation conditions to assess the cell-to-cell variation. Specifically, the current response was measured at total 81 electrodes from devices fabricated in 9 independent batches. As shown in **Fig. R21**, the normalized current values of all devices are tightly distributed around 1, with only small deviations among the tested positions. In addition, the average normalized current and corresponding standard deviation are comparable across all batches. These results indicate good film uniformity and reliable batch-to-batch reproducibility, supporting the suitability of the device for scalable and multi-site stimulation applications.

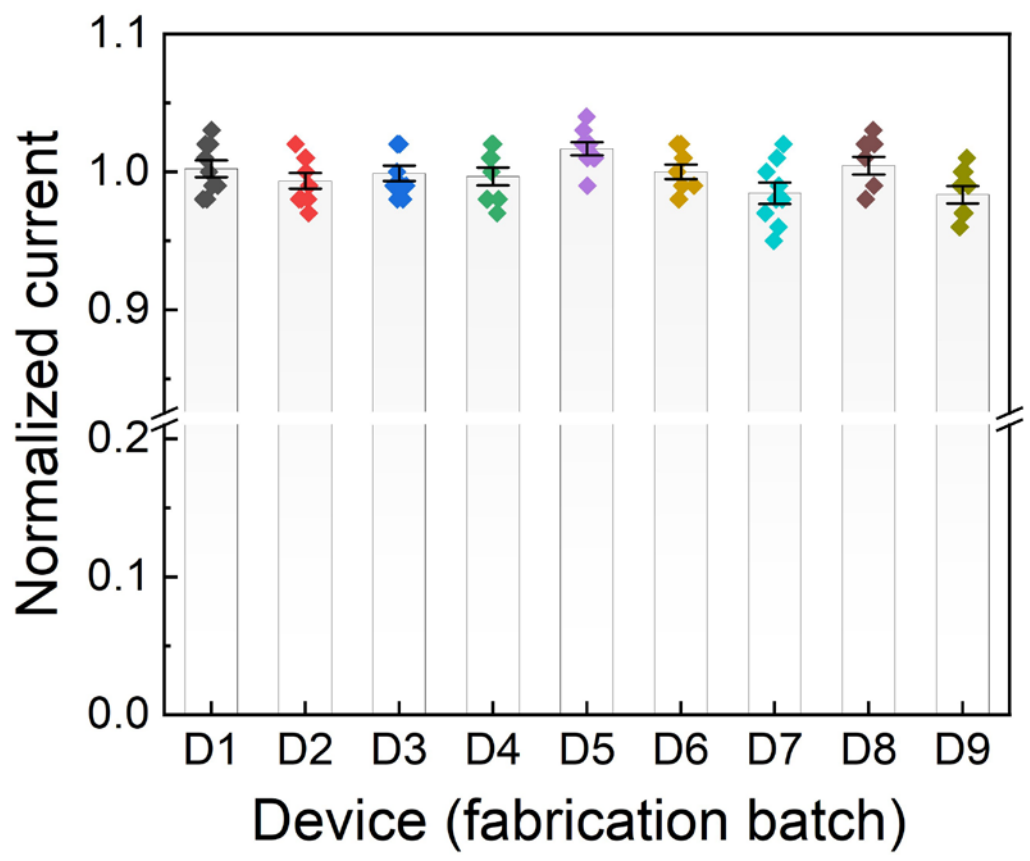

Fig. R21. Statistical analysis of normalized current measured at different positions for devices fabricated from nine independent batches. Individual data points represent the normalized current measured at different positions on each device. The error bars denote the standard deviation.

To address this comment, **Fig. R21** has been added as **Fig. S2**, and the description has been added in the revised manuscript (Page 10, highlight in blue).

Major-6. The main theme of this study is the capability of the OES to convert tissue-attenuated weak light into electrical signals for cardiac pacing and myocardial regeneration. While Figure 5 demonstrates the regenerative effects, the demonstration of cardiac pacing presented in Figure 6 was conducted under open-chest conditions in a porcine model. I acknowledge the technical difficulties associated with performing closed-chest stimulation in large animal models. However, to validate the device's advantage, I recommend that the authors include additional *in vivo* pacing data (just like Figure 6g, h, i) in rat or other small animals, but in closed-chest conditions.

Response:

We thank the Reviewer for this insightful and constructive suggestion. We agree that demonstrating cardiac pacing under closed-chest conditions would further validate the capability of the OES to convert tissue-attenuated weak light into electrical signals in a more physiologically relevant scenario.

In response to the Reviewer's comment, we have performed additional *in vivo* pacing experiments in Sprague-Dawley (SD) rats under closed-chest conditions. In this experiment, optical stimulation was delivered through the intact chest wall while the OES device was implanted on the epicardial surface. The results demonstrate reliable pacing capture, with the heart rate increasing from ~240 bpm to ~300 bpm during light stimulation, confirming that the OES device can effectively convert weak, tissue-attenuated light into electrical signals sufficient to drive cardiac pacing.

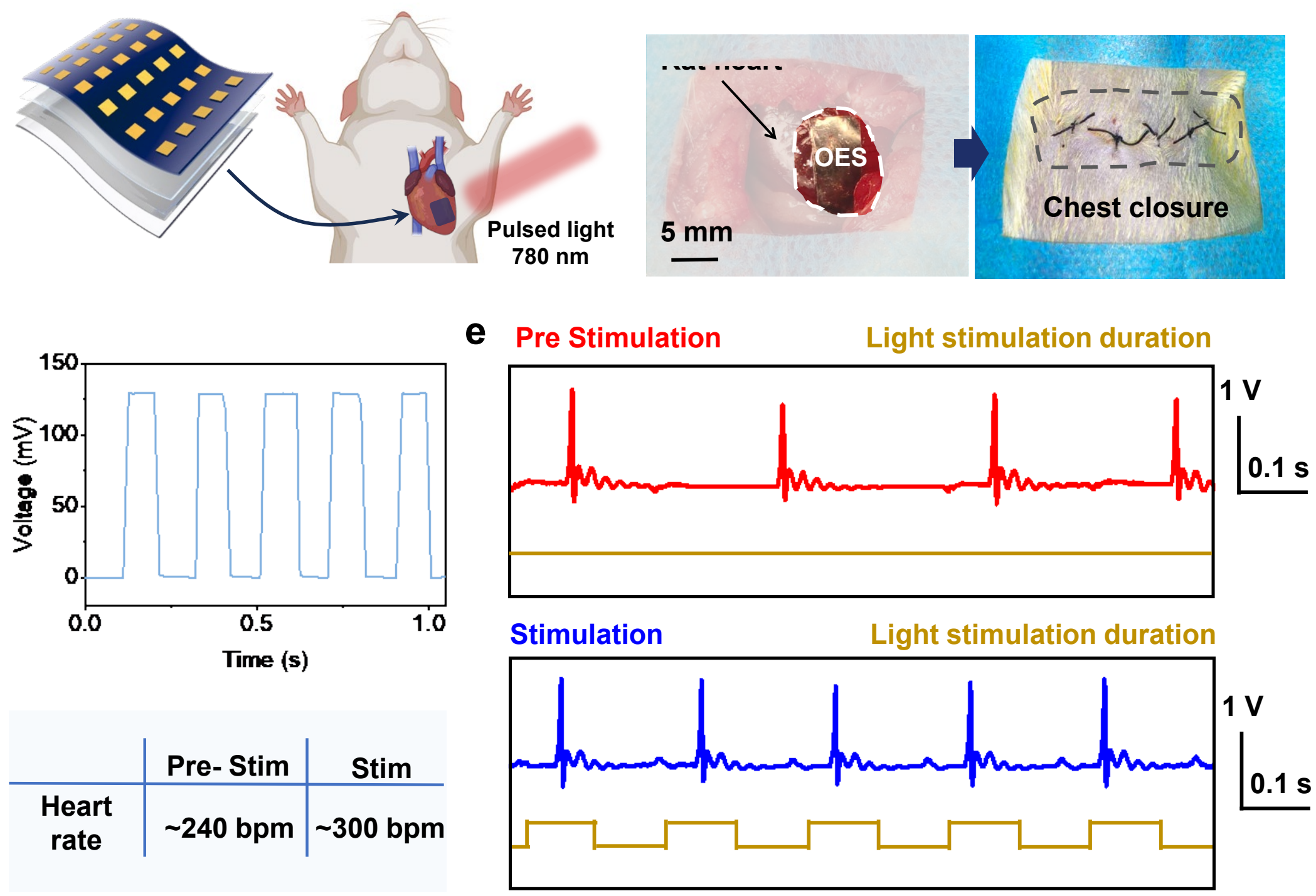

Fig R22. Closed-chest cardiac pacing enabled by the OES device in rats. (a) Schematic of the experimental setup illustrating OES-mediated cardiac pacing under closed-chest conditions, where externally delivered light is converted into electrical stimulation to modulate cardiac rhythm. (b) Photograph showing the placement of the OES on the left ventricular surface of the rat heart for pacing experiments (device size: 5 mm × 10 mm). (c) Representative V_{OC} generated by the OES under optical stimulation at 5 Hz. (d) Quantification of heart rate before and during optical stimulation, showing an increase from ~240 to ~300 bpm. (e) Representative electrocardiogram (ECG) traces recorded from the rat during the pre-stimulation and stimulation periods.

To address this comment, **Fig. R22** has been added as **Fig. S22**, and the description has been added in the revised manuscript (Page 9, highlight in red).

Minor comments:

Minor-1. Several minor text-related corrections appear to be needed. In Figure 2c, the label "Pmax" is clipped, and in Figure 2f, part of the "Voltage (V)" label is also cut off. In Figure 3d, the "Curvature radius (mm)" text is clipped, and in Figure 3i, the "Voltage (V)" label is partially cut off as well. In Figure 4d, the term "2mm skin" should be written with a space as "2 mm".

Response:

Thank you for your careful reading and valuable suggestions. We have carefully checked the figures and corrected the issues mentioned.

1. In Figure 2c, the label "P_{max}" was previously clipped. The figure has been adjusted to ensure the label is fully visible.

2. In Figure 2f, the "Voltage (V)" label was partially cut off. The layout of the figure has been modified to display the full label.

3. In Figure 3d, the "Curvature radius (mm)" text was clipped. The figure margins have been adjusted.

4. In Figure 3i, the "Voltage (V)" label was also partially cut off. This has been corrected in the revised figure.

5. In Figure 4d, the unit format has been revised from "2mm skin" to "2 mm skin" to comply with standard SI unit formatting.

To address this comment, all the above issues have been corrected in the revised manuscript and figures.

Minor-2. Current description of Figure 2c in the main text could lead readers to misinterpret the figure as plotting actual measured data. I suggest revising the main text to explicitly state that Figure 2c is a schematic representation of device's working principle.

Response:

We thank the Reviewer for pointing out the ambiguity. This question is similar to Reviewer 1/Major 3. In the original version, **Fig. 2c** was intended as a schematic illustration to define the classical photovoltaic parameters (*I*-*V* and *P*-*V* characteristics) rather than a measured curve, which indeed may lead to confusion.

To address this comment, we have therefore revised **Fig. 2c** and its caption to (i) explicitly state that this panel is a schematic plot, and (ii) define all abbreviations used in the schematic figure (I_{SC}, short-circuit current; V_{OC}, open-circuit voltage; M_{PP},

maximum power point; V_{mpp}/I_{mpp}, voltage/current at maximum power point; P_{max}, maximum output power). These changes improve readability for readers from diverse backgrounds.

Minor-3. In Figure 2k, it can be inferred from the context that the device is composed of six cells; however, the figure alone does not clearly indicate how many cells are connected in the module. It would be helpful to explicitly indicate the number of integrated cells within the figure.

Response:

Thank you for this valuable comment. Indeed, **Fig. 2k** illustrates the output voltage of a series-connected modules with six cells under varying incident light intensities. To improve clarity, we have marked the number in **Fig. 2k** and revised the figure caption, as below:

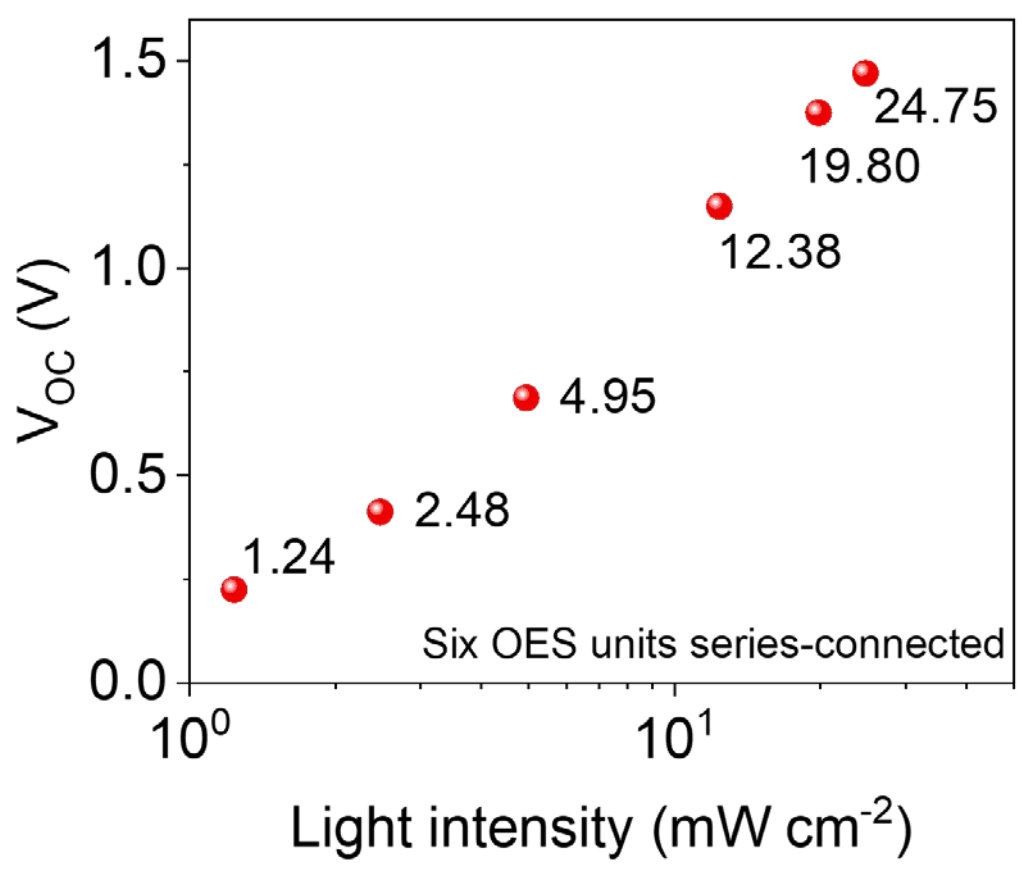

Fig. R23. *V*oc of a series-connected modules with six cells as a function of incident light intensity. The value at each point indicates the corresponding X-axis light intensity.

Minor-4. The text discusses stress distribution and cites figure 3b, but I guess it should be figure 3c. Please correct this citation.

Response:

Thank you for pointing this out. The Reviewer is correct that the citation should refer to **Fig. 3c** rather than **Fig. 3b**. We have corrected this error in the revised manuscript.

Minor-5. In Figure 3b, the presented image does not sufficiently show conformal contact between the device and the tissue. Providing a closer or higher-resolution view of the interface would better substantiate the conformal adhesion described in the manuscript.

Response:

We thank the Reviewer for this helpful suggestion. To better illustrate the conformal contact between the device and the tissue, as shown in **Fig. R24**, we have added a higher-resolution zoomed-in view of the interface. The enlarged image clearly shows the intimate and conformal adhesion between the device and the tissue surface.

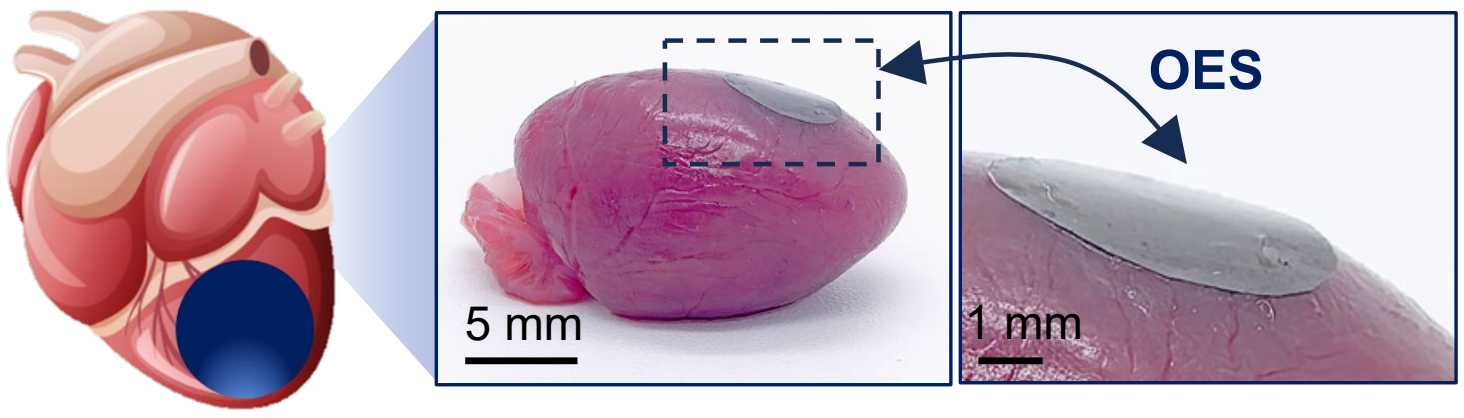

Fig. R24. The OES conforms tightly to cardiac tissue, exhibiting robust adhesive properties.

To address this comment, **Fig. 3b** has been replaced with **Fig. R24** in the revised manuscript.

Minor-6. In Figure 4c, y-axis variables are not defined either in figure caption or main text. I suggest adding a brief explanation.

Response:

Thank you for your helpful comment. In the revised manuscript, Specifically, the y-axis represents P1/P0, where P1 denotes the light intensity after passing through the skin/fat layer and P0 represents the original incident light intensity.

To address this comment, we have added a brief explanation in **Fig. 4c** caption to clarify this point.

Minor-7. When examining the graphs in Figure 4l, m, n, o, the use of different scales makes it difficult to visually compare the results. It would be preferable to unify the scales and display the data within a single graph using different colors, so that the differences can be interpreted more intuitively.

Response:

We thank the Reviewer for this helpful suggestion. In the revised manuscript, original Figs. 4l-o have been replotted using a unified scale and combined into a single graph with different colors to distinguish the datasets (**Fig. R25**).

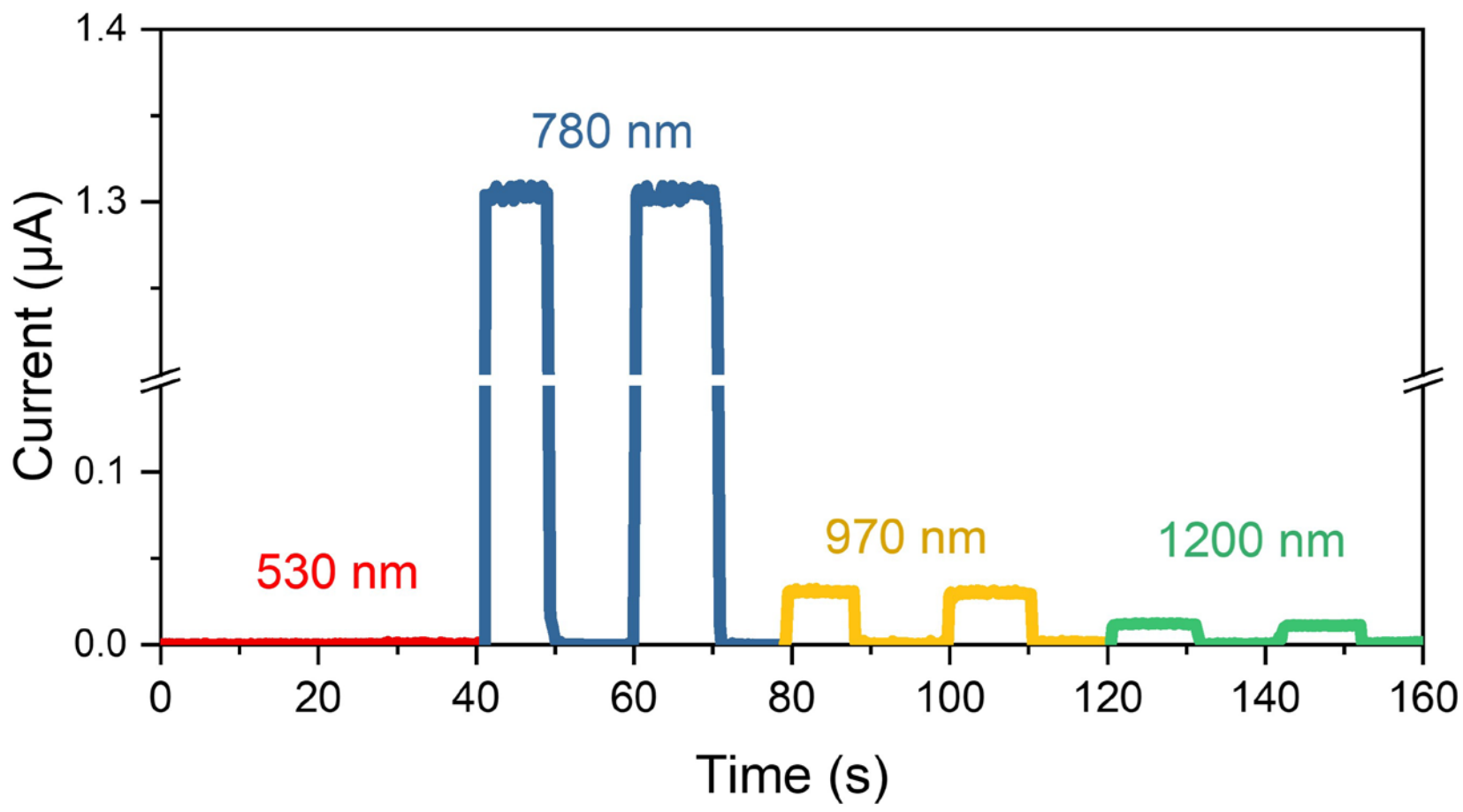

Fig. R25. Cardiac regions were irradiated with lasers of varying wavelengths (530 nm, 780 nm, 970 nm, and 1200 nm), revealing that the peak photocurrent response (~1,300 nA) was achieved at 780 nm.

To address this comment, **Figs. 4l-o** have been replaced with **Fig. R25** in the revised manuscript to more intuitive comparison of the results.

Minor-8. Figure 6a appears to be disconnected from the rest of Figure 6. To maintain the coherence of the figure, I suggest moving the fabrication schematic to the supplementary information. Furthermore, the current illustration in Figure 6a is bit hard to understand. It would be more effective to replace it with a clear, step-by-step diagram that shows sequential deposition

Response:

We thank the Reviewer for this helpful suggestion. While we understand the concern regarding the coherence of **Fig. 6**, we prefer to retain the fabrication schematic in the main text, as it provides essential context for understanding the device architecture and its relationship to the subsequent functional demonstrations presented

in Figure 6. Moving this schematic to the Supplementary Information may interrupt the logical flow and make it less convenient for readers to follow the device construction and operation.

Nevertheless, following the Reviewer's suggestion, we have substantially revised **Fig. 6a** to improve clarity. The schematic is now presented as a step-by-step fabrication diagram within the workflow, showing sequential deposition from the PI substrate (input) to the fully assembled device (output). Specifically, ITO and ZnO layers are labeled as Stage I and Stage II (magnetron sputtering), while $(Bi,Sb)_2Se_3$ and Au are labeled as Stage III and Stage IV (thermal evaporation). These modifications provide a clearer sequential deposition process and improve the overall coherence of **Fig. 6**.

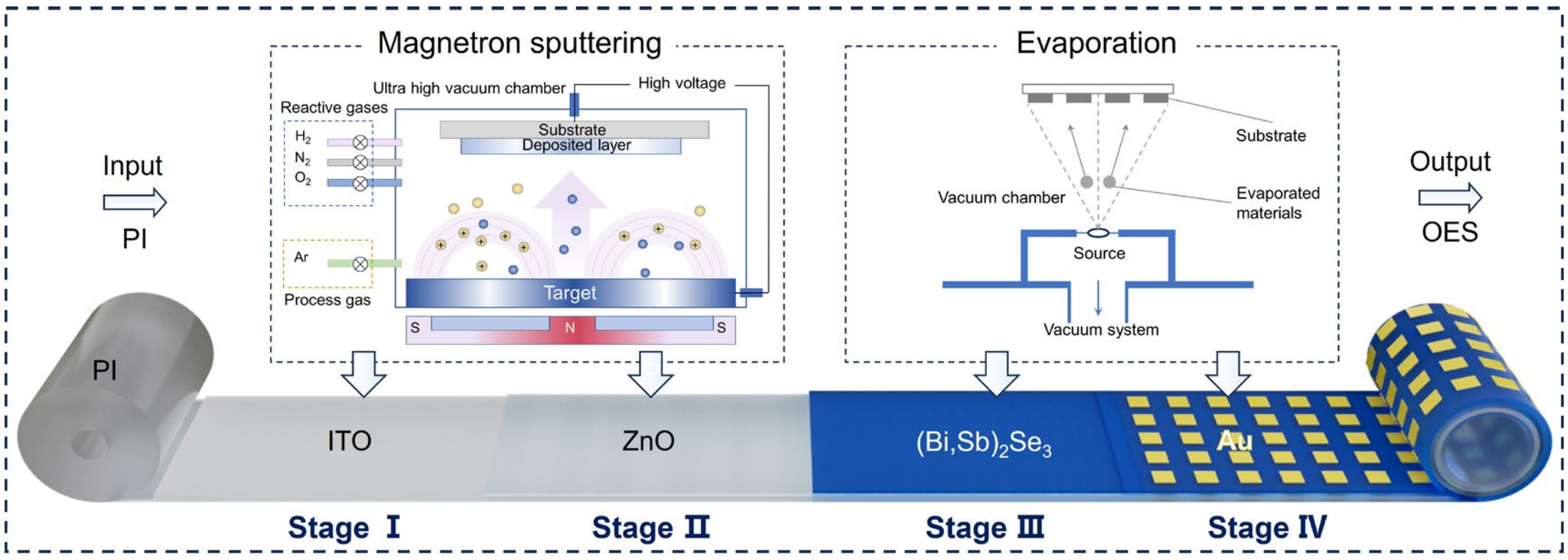

Fig. R26. Schematic diagram of the OES mass production process. Initially, the ITO layer and ZnO layer were deposited using the magnetron sputtering method. Subsequently, the $(Bi,Sb)_2Se_3$ layer and Au electrode were fabricated via the principle of thermal evaporation.

References:

1 Milligan, G. M., Yao, Z.-F., Cordova, D. L. M., Tong, B. & Arguilla, M. Q. Single Quasi-1D Chains of Sb2Se3 Encapsulated within Carbon Nanotubes. *Chemistry of Materials* **36**, 730-741 (2024). https://doi.org/10.1021/acs.chemmater.3c02114

2 Li, K. *et al.* Filter-free self-power CdSe/Sb2(S1−x,Sex)3 nearinfrared narrowband detection and imaging. *InfoMat* **3**, 1145-1153 (2021). https://doi.org/https://doi.org/10.1002/inf2.12237

3 Hamblin, M. R. Shining light on the head: Photobiomodulation for brain disorders. *BBA Clinical* **6**, 113-124 (2016). https://doi.org/https://doi.org/10.1016/j.bbacli.2016.09.002

4 Okuyama, S. *et al.* Avoiding thermal injury during near-infrared photoimmunotherapy (NIR-PIT): the importance of NIR light power density. *Oncotarget* **8**, 113194 (2017).

5 Li, K. *et al.* One-dimensional Sb2Se3 enabling ultra-flexible solar cells and mini-modules for IoT applications. *Nano Energy* **86**, 106101 (2021). https://doi.org/https://doi.org/10.1016/j.nanoen.2021.106101

6 Zhou, Y. *et al.* Thin-film Sb2Se3 photovoltaics with oriented one-dimensional ribbons and benign grain boundaries. *Nature Photonics* **9**, 409-415 (2015). https://doi.org/10.1038/nphoton.2015.78

7 Li, Z. *et al.* 9.2%-efficient core-shell structured antimony selenide nanorod array solar cells. *Nature Communications* **10**, 125 (2019). https://doi.org/10.1038/s41467-018-07903-6

8 Gao, R. *et al.* (Bi,Sb)2Se3 Alloy Thin Film for Short-Wavelength Infrared Photodetector and TFT Monolithic-Integrated Matrix Imaging. *Small* **20**, 2308070 (2024). https://doi.org/https://doi.org/10.1002/smll.202308070

9 Gao, R. *et al.* Boosting Performance of ZnO/(Bi,Sb)2Se3 Short-Wavelength Infrared Photodetector via ZnTe Hole-Transport Layer. *Advanced Optical Materials* **13**, e01610 (2025). https://doi.org/https://doi.org/10.1002/adom.202501610

10 Rahko, P. S. Evaluation of the Skin-To-Heart Distance in the Standing Adult by Two-Dimensional Echocardiography. *Journal of the American Society of Echocardiography* **21**, 761-764 (2008). https://doi.org/https://doi.org/10.1016/j.echo.2007.10.027

11 Yashina, L. V. *et al.* Negligible Surface Reactivity of Topological Insulators Bi2Se3 and Bi2Te3 towards Oxygen and Water. *ACS Nano* **7**, 5181-5191 (2013). https://doi.org/10.1021/nn400908b

12 Wang, Z. *et al.* Chronic Intermittent Low-Level Transcutaneous Electrical Stimulation of Auricular Branch of Vagus Nerve Improves Left Ventricular Remodeling in Conscious Dogs With Healed Myocardial Infarction. *Circulation: Heart Failure* **7**, 1014-1021 (2014). https://doi.org/10.1161/CIRCHEARTFAILURE.114.001564

13 Uitterdijk, A. *et al.* Intermittent pacing therapy favorably modulates infarct remodeling. *Basic Research in Cardiology* **112**, 28 (2017). https://doi.org/10.1007/s00395-017-0616-3

Reviewer #1

Minor corrections:

1. Main text:

a. Page 3 please specify the PI thickness in the text, i e 8 um, as this is critical information for the readers

Response:

We thank the Reviewer for this helpful suggestion. The thickness of the PI substrate has now been explicitly specified in the revised manuscript.

b. For Fig 1 caption, please specifically not that there is no physical separation between the layers as it can be very misleading

Response:

We thank the Reviewer for pointing this out. We have revised the Figure 1 caption to clarify that the layers are shown separated only for visualization purposes and that no physical separation exists in the actual device structure.

c. For Fig 2 caption please specify the size of the spot used for the device mapping a,b , please specify in the caption that for e onwards it's not focused, still it would be useful to know the size of illumination, are we talking about 5x5mm or 1x1mm or 1x1cm. This certainly makes a difference for reproducibility of the reported results.

Response:

We thank the Reviewer for this helpful suggestion regarding the illumination conditions. We have now explicitly clarified that a 780 nm LED with a localized illumination spot of approximately 4.5 mm in diameter was used throughout the photocurrent mapping experiments.

This information has been added to both the Fig. 2 caption and the Methods section in the Supplementary Information to ensure consistency and improve reproducibility. The illumination corresponds to a mesoscale LED spot rather than a focused microscopic beam, and was sequentially positioned across the device to obtain the spatial photocurrent response.

d. for the cardiac pacing, it is interesting that the device does not show any slippage from the beating heart, was it sutured or adhered with some gel? as it reads at the moment, the device is just placed on top of the tissue directly, however one would wonder how the adhesion to the tissue was promoted.

Response:

We thank the Reviewer for this comment. In the cardiac pacing experiments, no

sutures, adhesives, or hydrogel materials were used. The device was directly placed on the epicardial surface of the beating heart for short-term measurements.

Stable contact is enabled by the ultrathin and compliant nature of the device, allowing conformal contact with the wet tissue surface, together with van der Waals interactions and capillary effects during the measurement period.

This clarification has been added to the Methods sections 18 and 19 of the revised manuscript.

2. Supplementary, 1 Device fabrication:

a. For the gold electrode deposition, please include the thickness of gold deposited, right now it says 80um, which is either a typo (i.e., 80nm) or a size of one of the squares.

Response:

We thank the Reviewer for carefully identifying this typographical error. The originally stated “80 μm” was incorrect. The correct thickness of the gold electrode is 80 nm. The manuscript has been corrected accordingly.

3. Supplementary, 2 Photocurrent mapping:

a. What was the spot size? i e 10um or 1mm? this helps to interpret correctly the presented photocurrent maps. Additionally it helps to understand the regime of illumination used to extract the Voc parameters, please also include this information in the caption for fig 2 as discussed above.

Response:

We thank the Reviewer for this helpful comment. We have now clarified that a 780 nm LED with a localized illumination spot of approximately 4.5 mm in diameter was used for both photocurrent mapping and Voc measurements. The corresponding information has been added to the Fig. 2 caption and Supplementary Methods to improve clarity and reproducibility.

Reviewer #2

As described on page 4 (end of the first paragraph), the photodetector architecture generates photocarriers that accumulate at the low-potential (ITO) and high-potential (gold) terminals. While this effect is correct in an optoelectronic device, it raises a critical concern (initially raised in major comment 2 in the previous review report) regarding the spatial distribution of the resulting electric field. Because the generated potential appears to be primarily "buried" within the film, or in-between the two electrodes, there is no evident overlap between the electric field and the target tissue. This reviewer acknowledges that this lack of field penetration is not an problem for the in vitro experiments, where the photodetector functioned as an energy harvester connected to the interdigitated electrodes. The authors must provide a clear, physical description of how the electric field generated within the photodetector is projected externally to reach the tissue in contact with the gold electrodes.

Response:

We thank the Reviewer for this important comment. Although the device adopts a vertical photodetector architecture, both Au and ITO electrodes are routed to the same tissue-contacting surface. Therefore, the photogenerated vertical potential is converted into a lateral potential difference at the tissue-contacting surface. When in contact with biological tissue, this interfacial potential difference drives ionic currents in the surrounding conductive medium, resulting in local electrochemical stimulation.

In other words, the OES functions as a self-powered photovoltage source, while the actual stimulation field is established outside the device, in the tissue/electrolyte, rather than within the multilayer stack itself. This is the same physical principle underlying conventional electrode-based bioelectronic stimulation, except that here the driving bias is generated optically and locally by the photovoltaic device. This clarification has been added to the Methods sections 18.
