# Supplementary material for: Ultra-flexible optoelectronic stimulator converts tissue-attenuated weak light into electrical signals for cardiac remodeling
Source: Nat Commun. 2026 Jul 13;17:8622. doi: 10.1038/s41467-026-75495-7 (PMC13486475; doi:10.1038/s41467-026-75495-7)
Supplement: Supplementary file 3 [file 41467_2026_75495_MOESM1_ESM.pdf]

Supporting Information For

Ultra-flexible Optoelectronic Stimulator Converts Tissue-Attenuated Weak Light into Electrical Signals for Cardiac Remodeling

Ruiyue Zhao[1#], Feifan Yang[2#], Tao Yang[3#], Kanghua Li[2*], Ruisi Gao[2], Xinchang Kang[1], Qi Chen[1], Liang Li[2], Jia Chen[1], Yi Yuan[1], Xinyi Chen[2], Mengxue Zhou[1], Shengmin Zhang[1], Jianglin Wang[1,4*], Chuanbin Mao[3*]

[1] Department of Biomedical Engineering; National Medical Products Administration Research Base for Regulatory Science of Medical Devices; State Key Laboratory of New Textile Materials and Advanced Processing, Huazhong University of Science and Technology, Wuhan, China.

[2] School of Integrated Circuits, Huazhong University of Science and Technology, Wuhan, China.

[3] Department of Biomedical Engineering, The Chinese University of Hong Kong, Sha Tin, Hong Kong, China.

[4] Shenzhen Huazhong University of Science and Technology Research Institute, Shenzhen, 518000, China.

[#] These authors contributed equally: Ruiyue Zhao, Feifan Yang, Tao Yang

* Corresponding Authors:

Chuanbin Mao

E-mail: maophage@gmail.com

Jianglin Wang

E-mail: jwang520@hust.edu.cn

Kanghua Li

E-mail: Kanghua_li@hust.edu.cn

I. Supplementary Tables

Table S1. Layer thickness, Young's modulus, and Poisson's ratio for each functional layer

Material	**Thickness**	**Young's modulus**	**Poisson's ratio**
Au	0.08 μm	78 GPa	0.42
$(Bi,Sb)_2Se_3$	0.8 μm	83 GPa	0.26
ZnO	0.34 μm	200 GPa	0.34
ITO	0.2 μm	135 GPa	0.26
PI	8/40 μm	3 GPa	0.4

II. Supplementary Figures

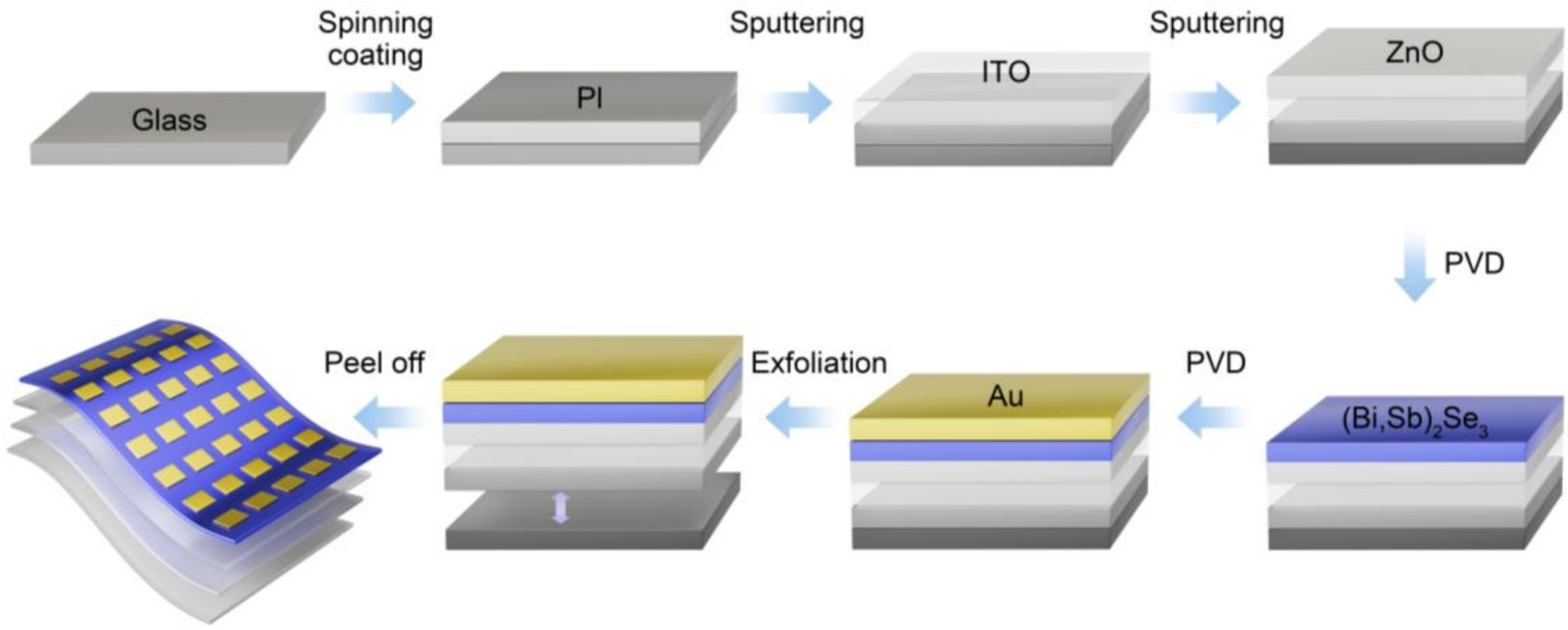

Fig. S1. Schematic illustration of the fabrication process for flexible $(Bi,Sb)_2Se_3$ optoelectronic stimulation devices. The process involves sequential steps including PI substrate preparation, deposition of ITO, n-type ZnO, and p-type $(Bi,Sb)_2Se_3$, followed by Au electrode, leading to the formation of a flexible and functional optoelectronic interface suitable for stimulation applications.

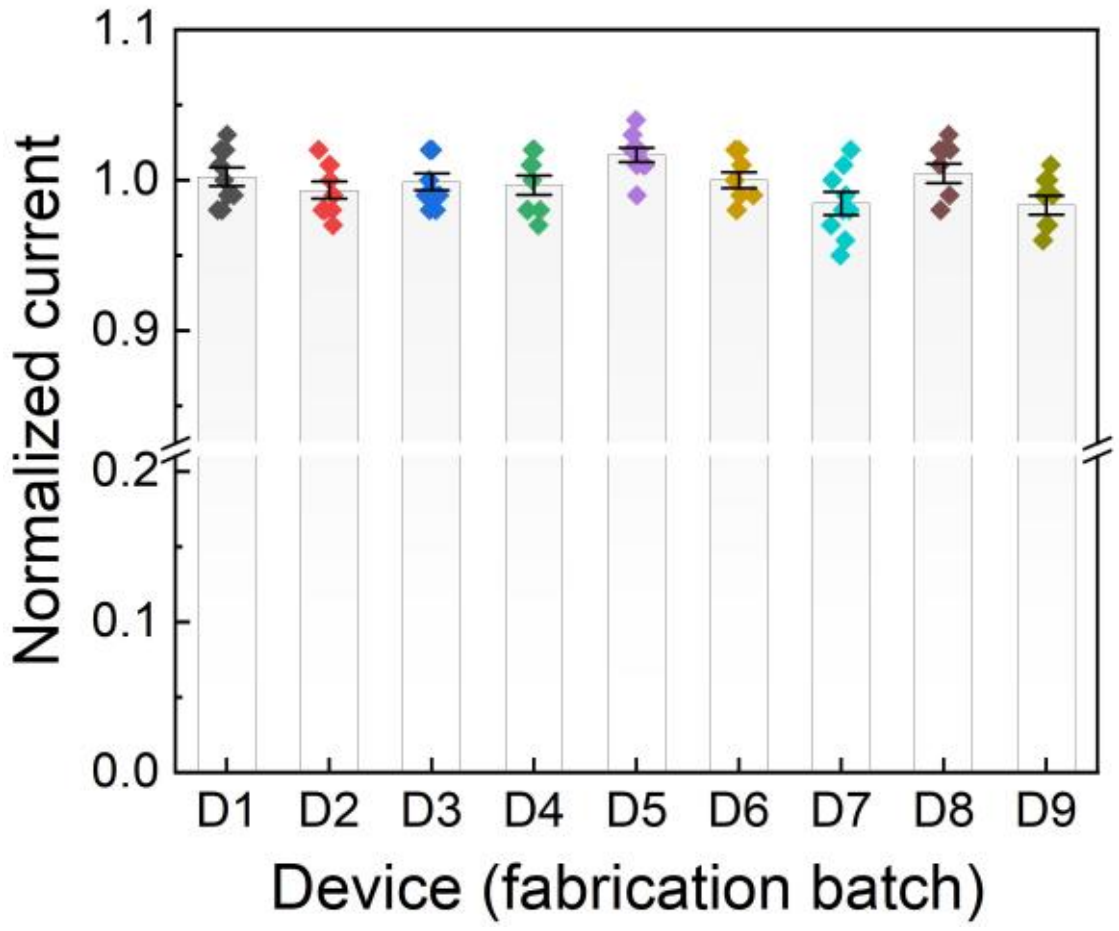

Fig. S2. Statistical analysis of normalized current measured at different positions for devices fabricated from nine independent batches. Individual data points represent the normalized current measured at different positions on each device. The error bars denote the standard deviation. Data in Fig. S2 are presented as mean ± S.D.; $n = 9$ independent experiments. Source data are provided as a Source Data file.

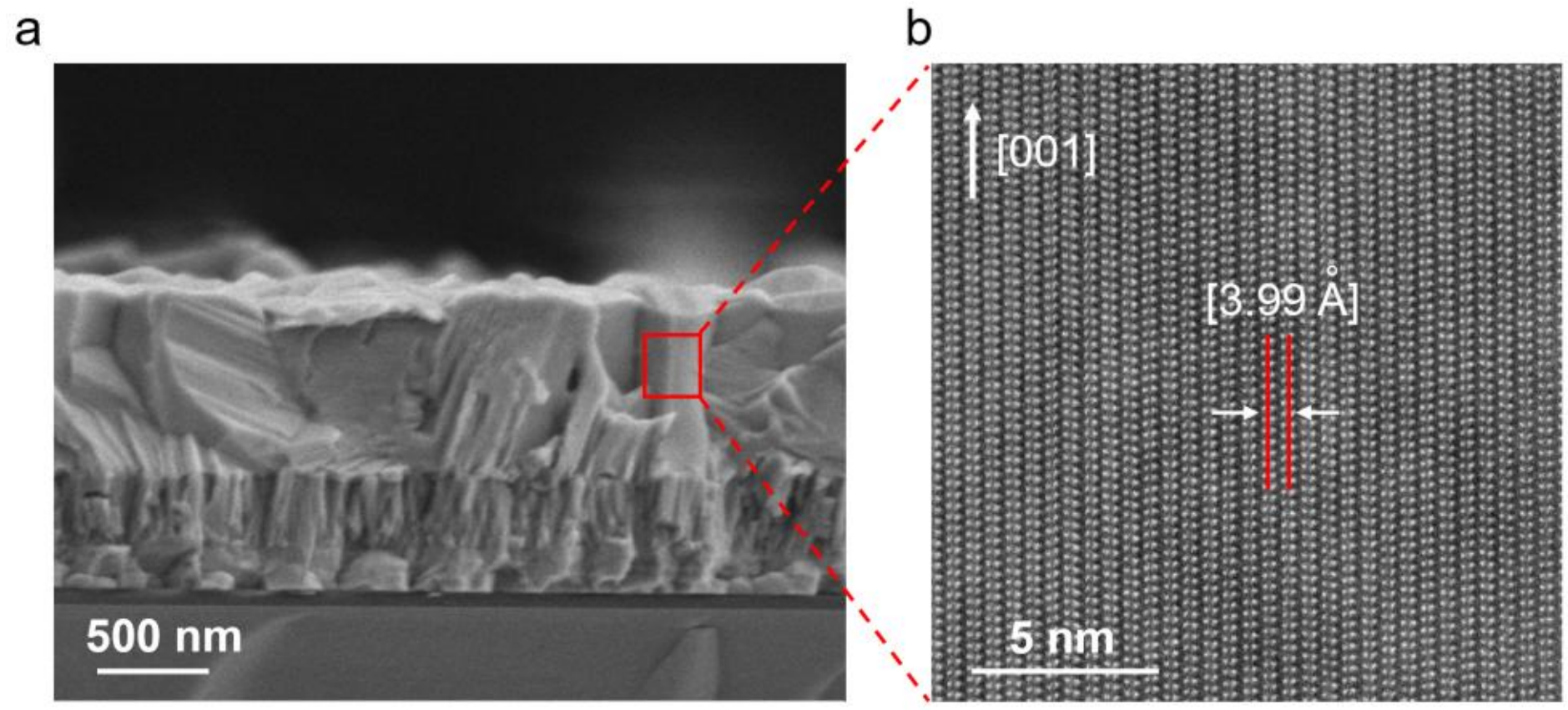

Fig. S3. Morphology of (a) vertically oriented columnar grains in the cross-section of the $(Bi,Sb)_2Se_3$ layer, and (b) the corresponding magnified HRTEM image.

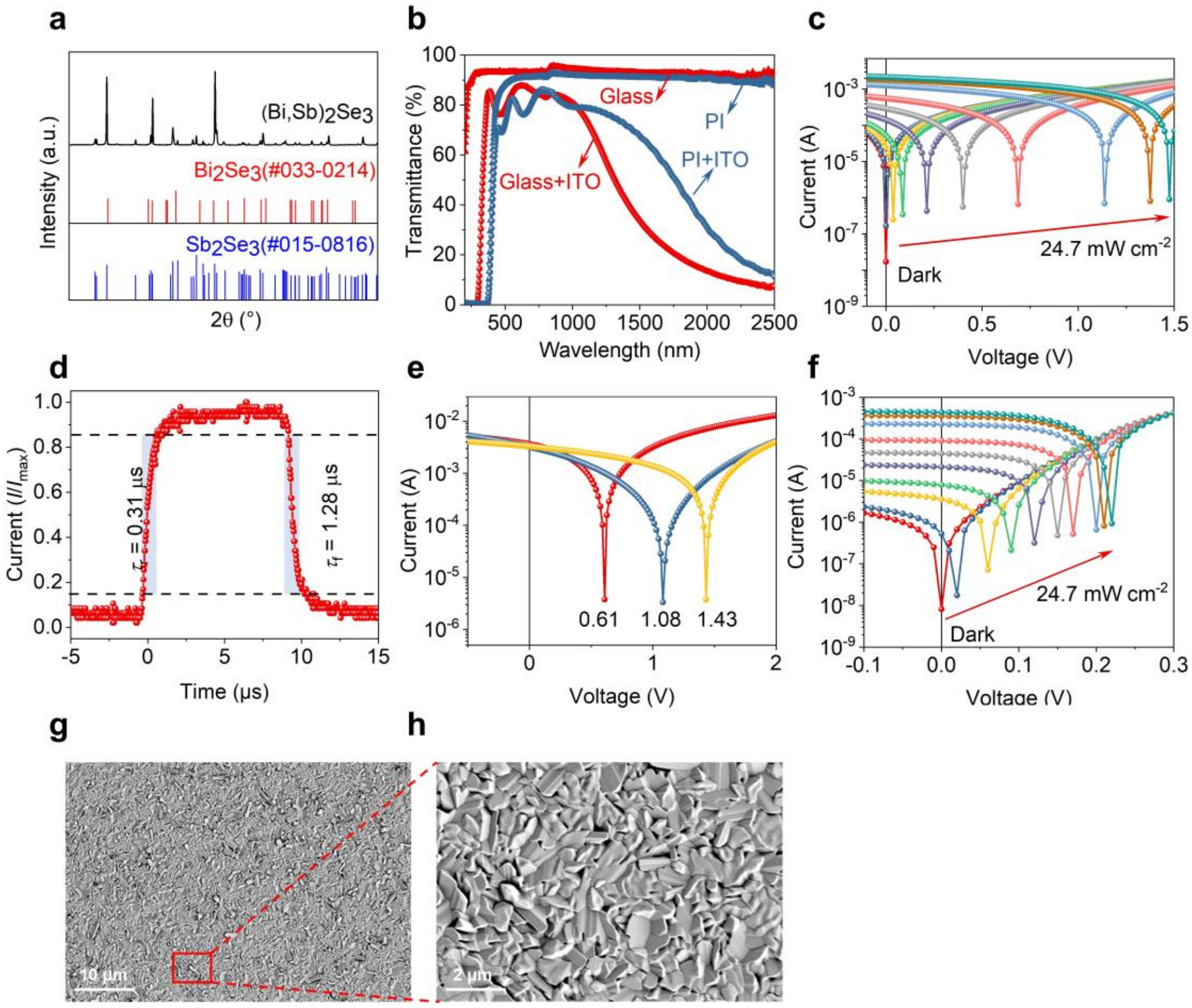

Fig. S4. Structural and photoelectric characterization of the OES. (a) XRD pattern of the $(Bi,Sb)_2Se_3$ thin film. (b) Photo-response speed of the OES device. (c) Optical transmittance spectra of glass, glass/ITO, PI, and PI/ITO substrates across different wavelengths. (d) Current-voltage (*I-V*) characteristics of the OES device under 780 nm illumination at varying light intensities. (e) *I-V* curves of OES mini-modules composed of 2, 4, and 6 units in series under 780 nm illumination. (f) *I-V* characteristics of the OES mini-module under 780 nm illumination with varying light intensities. (g-h) Plan-view SEM images of the surface morphology of the $(Bi,Sb)_2Se_3$ film: (g) low-magnification and (h) high-magnification views.

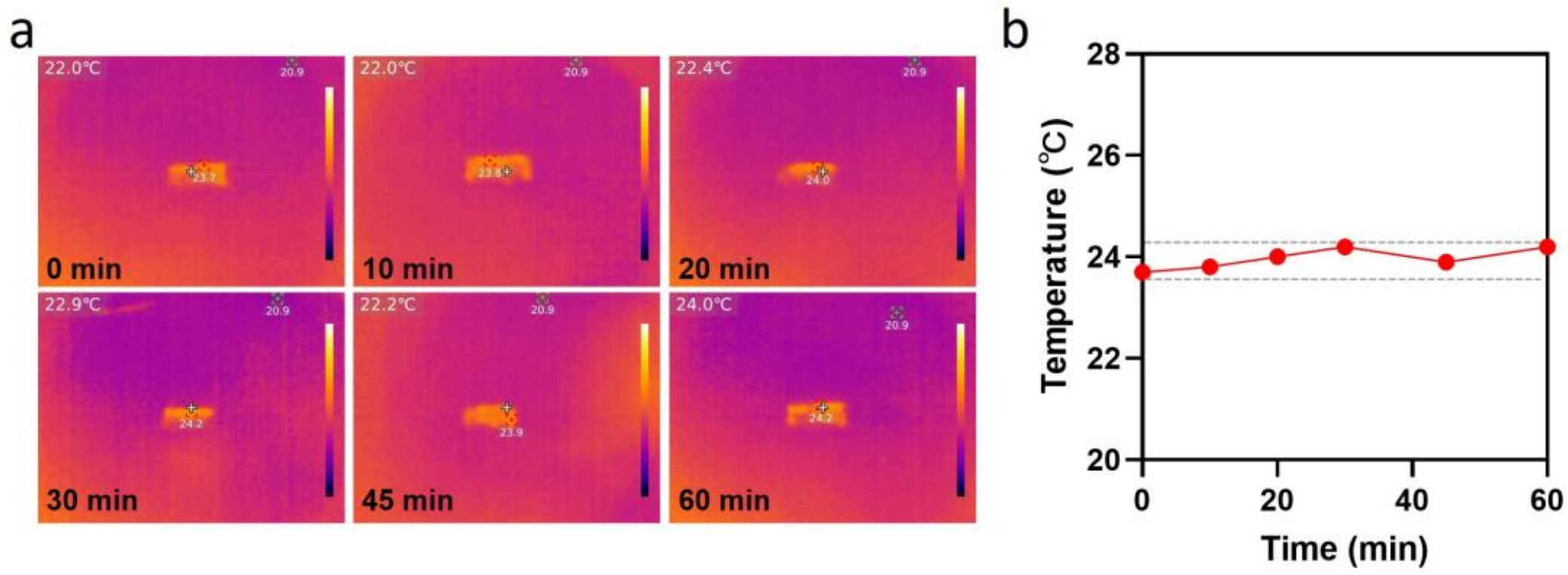

Fig. S5. Photothermal response of the OES under pulsed light illumination. (a) Infrared thermal images acquired at different time points during pulsed light stimulation (24.7 mW cm^{-2}, 1 Hz). The temperature indicated by the red cursor corresponds to the OES, the green cursor denotes the minimum temperature within the captured frame, and the value shown in the upper-left corner represents the average temperature of the entire image. (b) Statistical analysis of the temperature variation, showing a minimal fluctuation (≈0.5 °C over 1 h), which confirms stable thermal behavior and negligible photothermal effects.

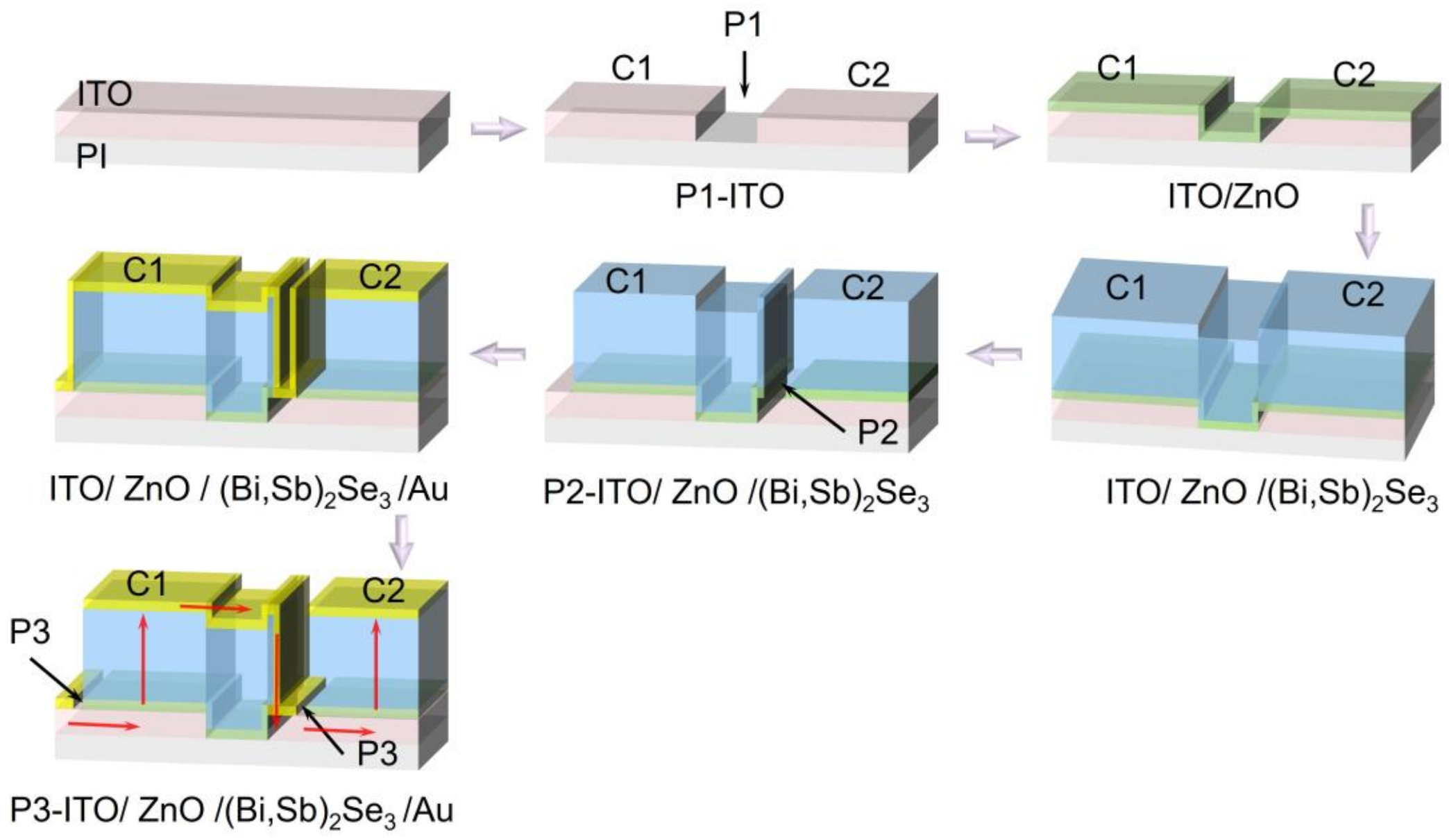

Fig. S6. Schematic of the micro-module fabrication flowchart. The fabrication details can be found in the Methods section.

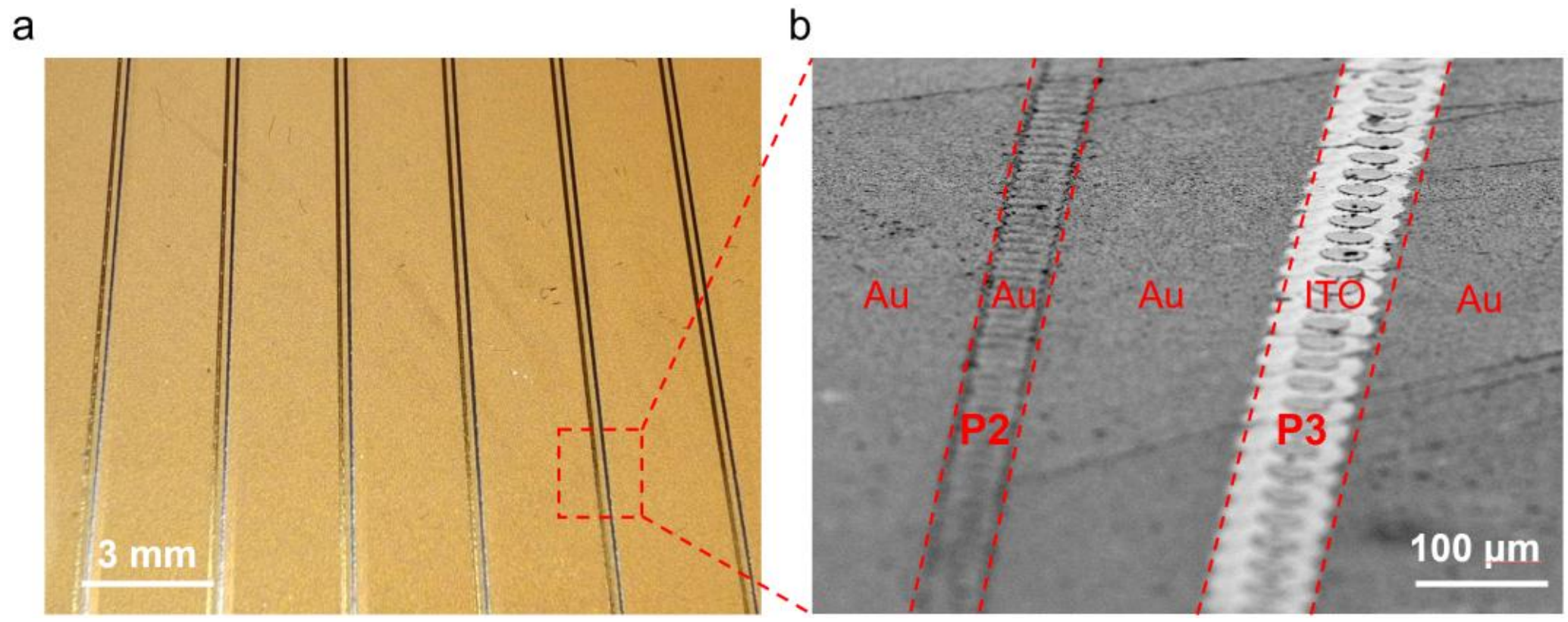

Fig. S7. (a) Photograph of the micro-modules and (b) SEM images of representative regions. The P1 scribe is shallow and subsequently covered by ZnO/$(Bi,Sb)_2Se_3$/Au layers, making it almost invisible in the image.

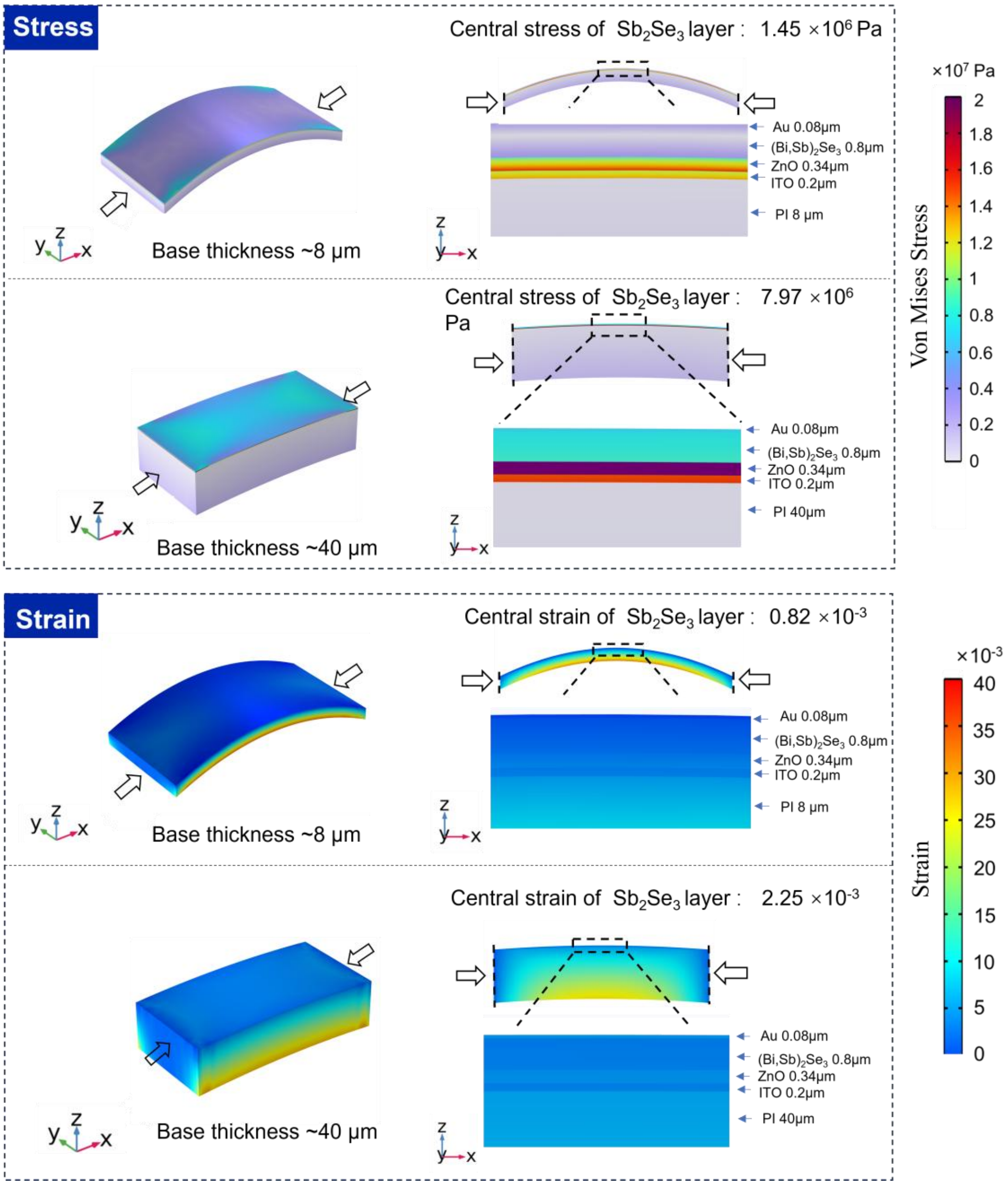

Fig. S8. COMSOL simulation of mechanical stress and strain in OES devices under bending. Simulated distributions of stress and strain in OES devices with substrate thicknesses of 8 μm and 40 μm under identical bending conditions. The simulations reveal markedly reduced mechanical deformation in the functional layer when employing the thinner (8 μm) PI substrate. Finite element parameters used in the model, including thickness, Young's modulus, and Poisson's ratio for each functional layer are detailed in **Table S1.**

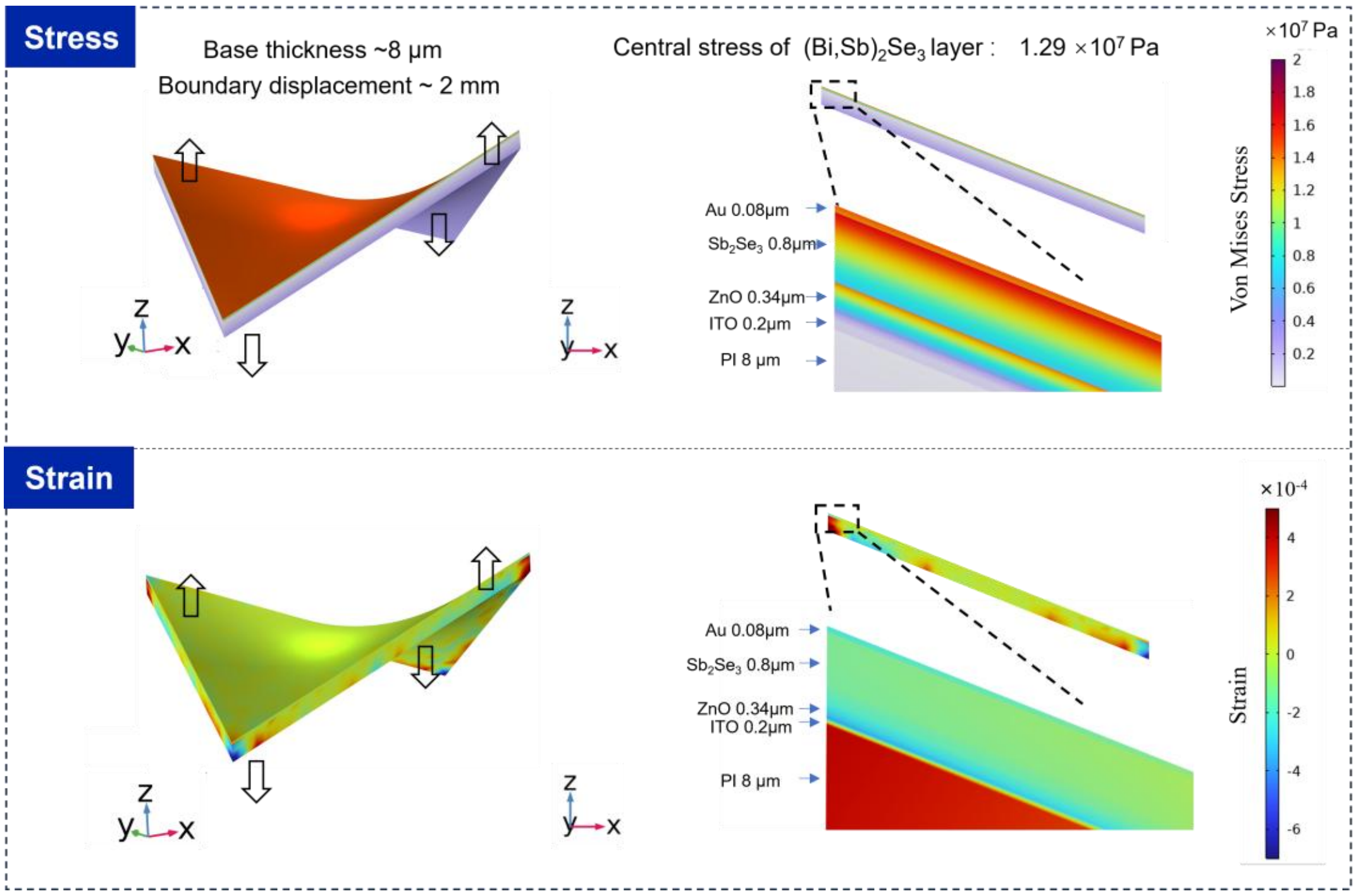

Fig. S9. COMSOL simulation of stress-strain distribution in OES devices under twist deformation. Finite element analysis of OES devices under twist deformation revealed that the central $(Bi,Sb)_2Se_3$ layer experienced a peak stress of ~1.29×10^7 Pa. Reducing the substrate thickness led to a marked decrease in both stress and strain within the functional layer, highlighting the mechanical advantage of ultrathin substrates in flexible device architectures.

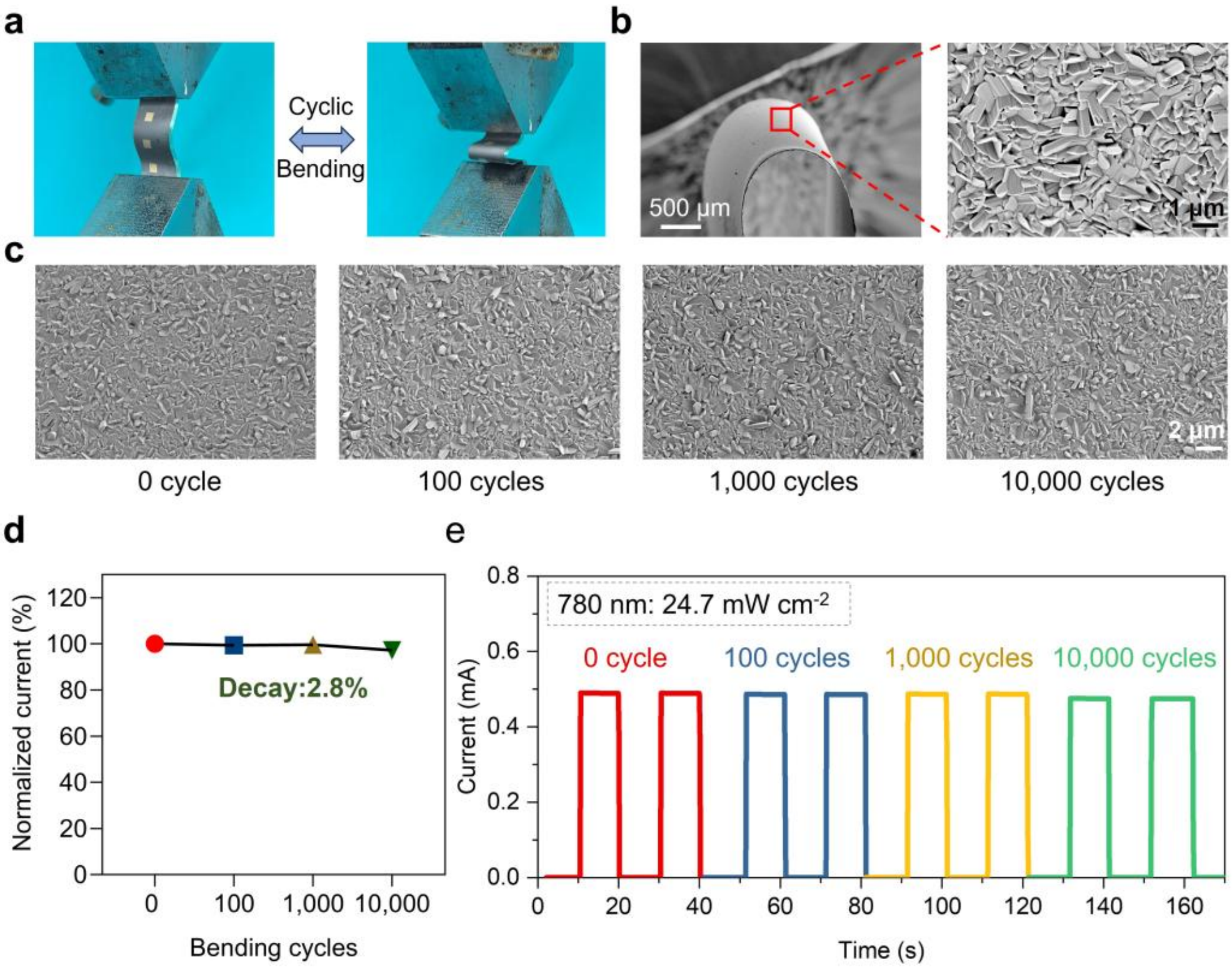

Fig.S10. (a) Photographs of the cyclic bending test. (b) In situ and higher-magnification surface SEM images of the OES in the bent state at a bending radius of 1 mm. (c) SEM images of the OES after 0, 100, 1,000, and 10,000 bending cycles. (d) Normalized photocurrent of the OES measured after 0, 100, 1,000, and 10,000 bending cycles. (e) Time-resolved photocurrent (*I-T*) response of the OES under repeated light on/off cycles (780 nm, 24.7 mW^{-2}).

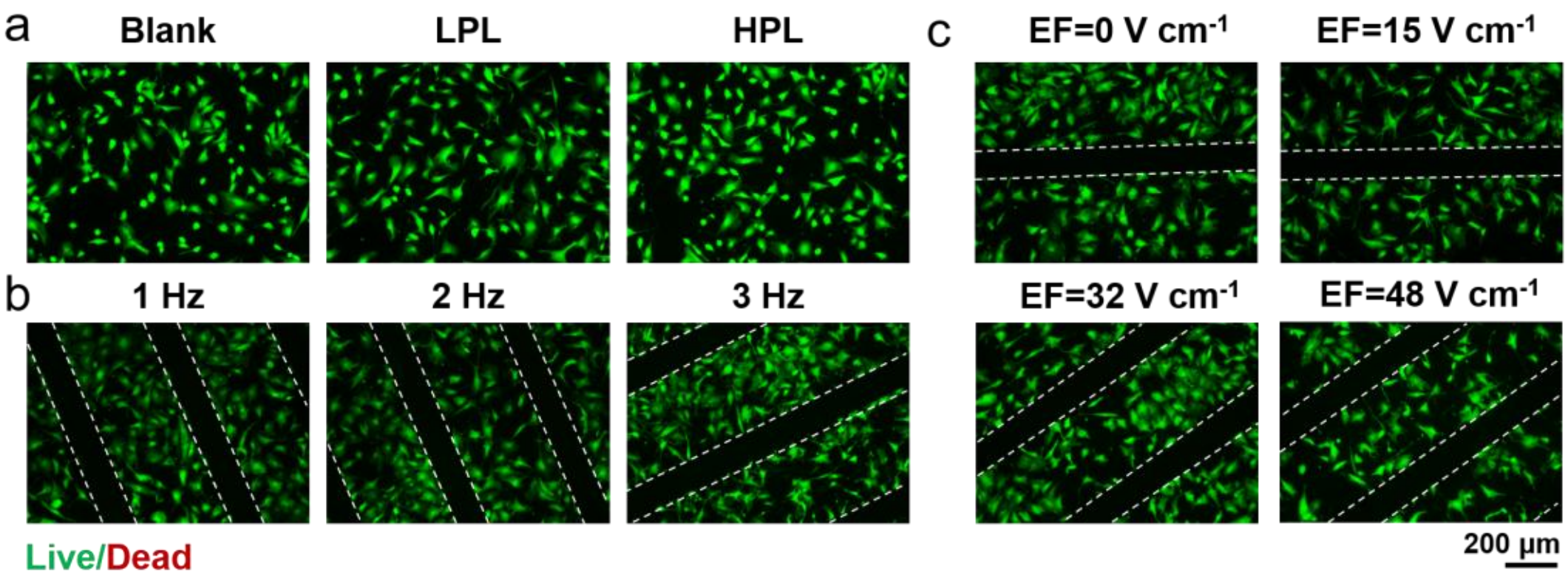

Fig. S11. Live/dead staining of NRCMs under different conditions. (a) NRCMs exposed to optical illumination at an identical power density without the device, showing negligible phototoxicity. (b) NRCMs after optoelectronic stimulation at different frequencies. (c) NRCMs after stimulation under different electric field strengths. Dashed lines indicate the electrode positions. (LPL: low power light; HPL: high power light).

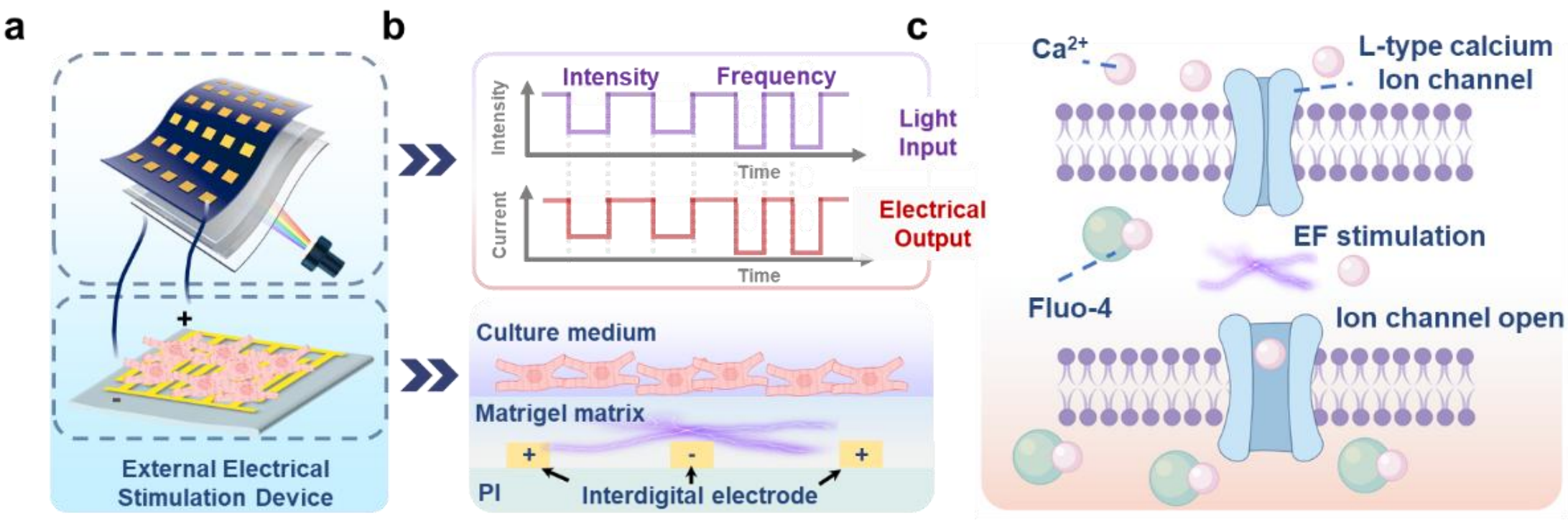

Fig. S12. Electrical stimulation modulates myocardial cell activity. Created in BioRender. ZHAO, R. (2026) https://BioRender.com/y3hfpkw. (a, b) Schematic illustration of the experimental setup showing externally applied electrical stimulation to myocardial cells cultured on Matrigel-coated interdigital electrodes. (c) Exposure to electric field (EF) stimulation enhances intracellular calcium signaling, indicating activation and upregulation of calcium ion channels in myocardial cells.

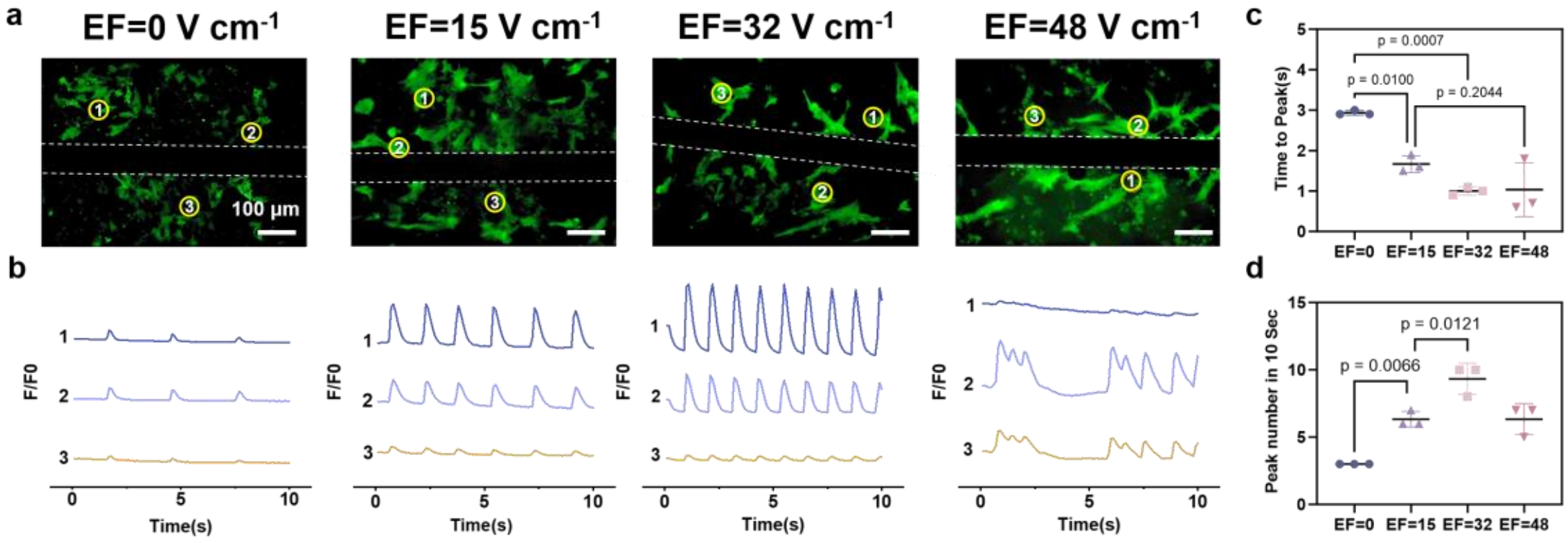

Fig. S13. Calcium dynamics of cardiomyocytes under varying electric field intensities. (a, b) Time-lapse calcium transient imaging of cardiomyocytes subjected to electric field (EF) stimulation ranging from 0 to 48 V cm^{-1}. (c) Quantification of Ca^{2+} transient time-to-peak under different EF intensities, based on imaging in b. (d) Number of Ca^{2+} transient peaks detected every 10 s under varying EF intensities. Frequency-domain analysis of the extracted signals identified 32 V cm^{-1} as the optimal stimulation intensity for synchronized calcium activity. Data in Fig. S13c, d are presented as mean ± S.D.; n = 3 independent experiments. Source data are provided as a Source Data file.

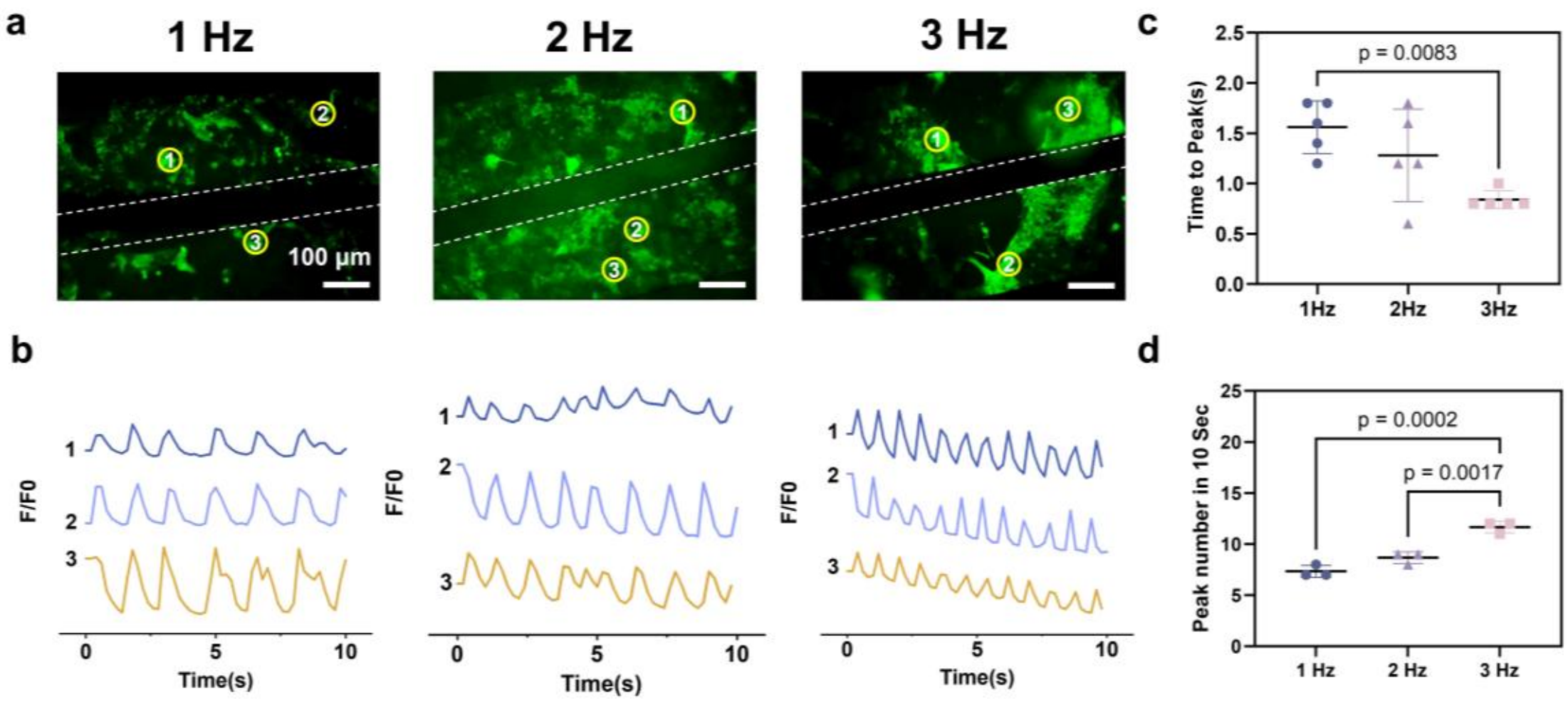

Fig. S14. Calcium dynamics of cardiomyocytes under varying frequency. (a, b) Calcium transient imaging of cardiomyocytes subjected to electric field (EF) stimulation at frequencies ranging from 1 to 3 Hz. (c) Quantification of Ca^{2+} transient time-to-peak under different stimulation frequencies, based on imaging in b. (d) Number of Ca^{2+} transient peaks detected every 10 s across frequencies. Frequency-domain analysis of the extracted signals identified 3 Hz as the optimal stimulation frequency for enhanced calcium signaling. Data in Fig. S14c are presented as mean ± S.D.; *n* = 5 independent experiments. Data in Fig. S14d are presented as mean ± S.D.; *n* = 3 independent experiments. Source data are provided as a Source Data file.

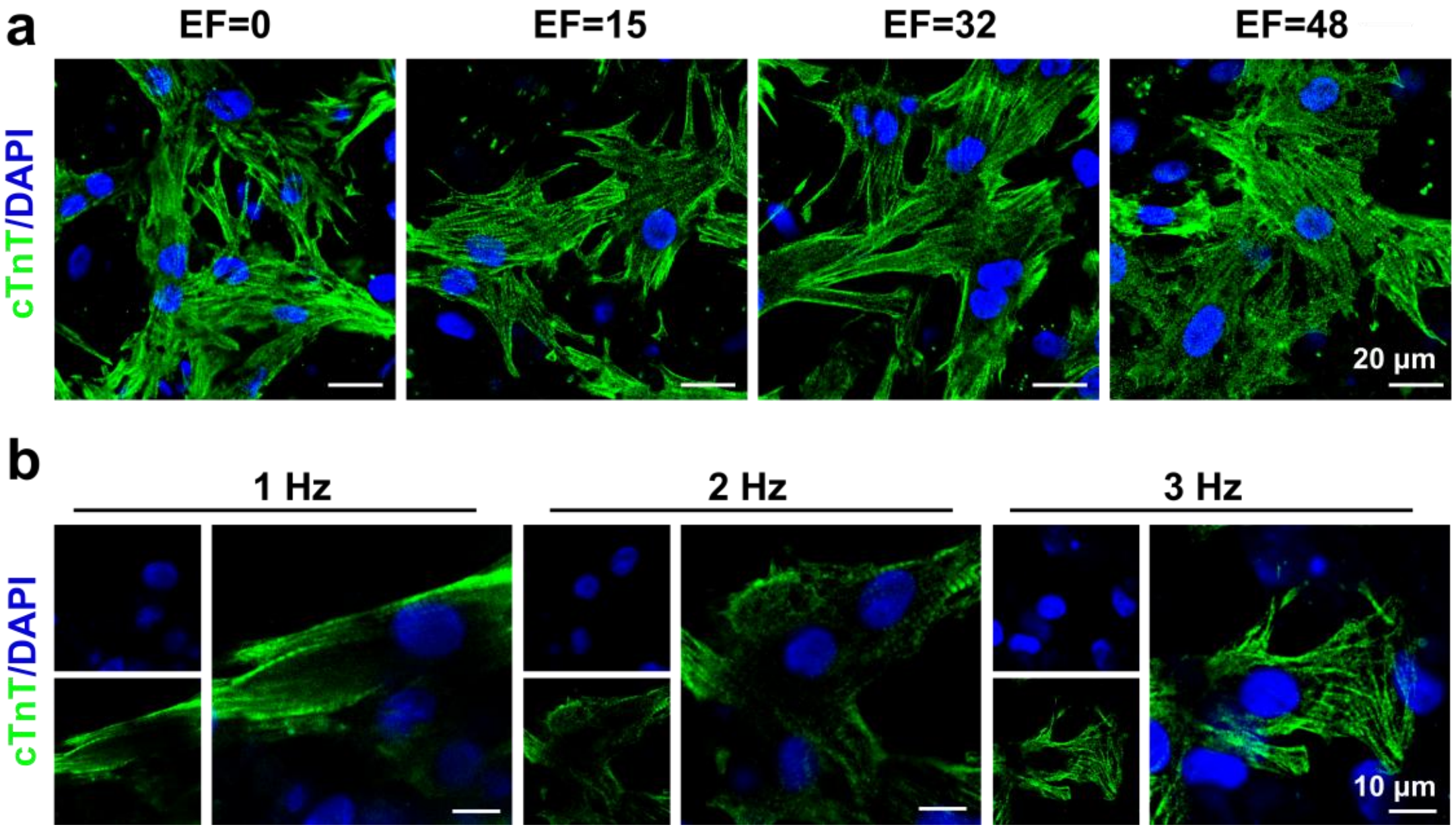

Fig. S15. Electrical stimulation modulates cardiomyocyte phenotype under varying intensity and frequency. (a) Immunofluorescence staining of neonatal rat ventricular cardiomyocytes (NRVCMs) after 4 days of culture under varying electric field (EF) intensities (unit: V cm^{-1}), showing cardiac troponin T (cTnT, green) and nuclei (blue). (b) Representative images of NRVCMs subjected to different stimulation frequencies, highlighting frequency-dependent modulation of cardiomyocyte phenotype. All images were acquired via confocal laser scanning microscopy.

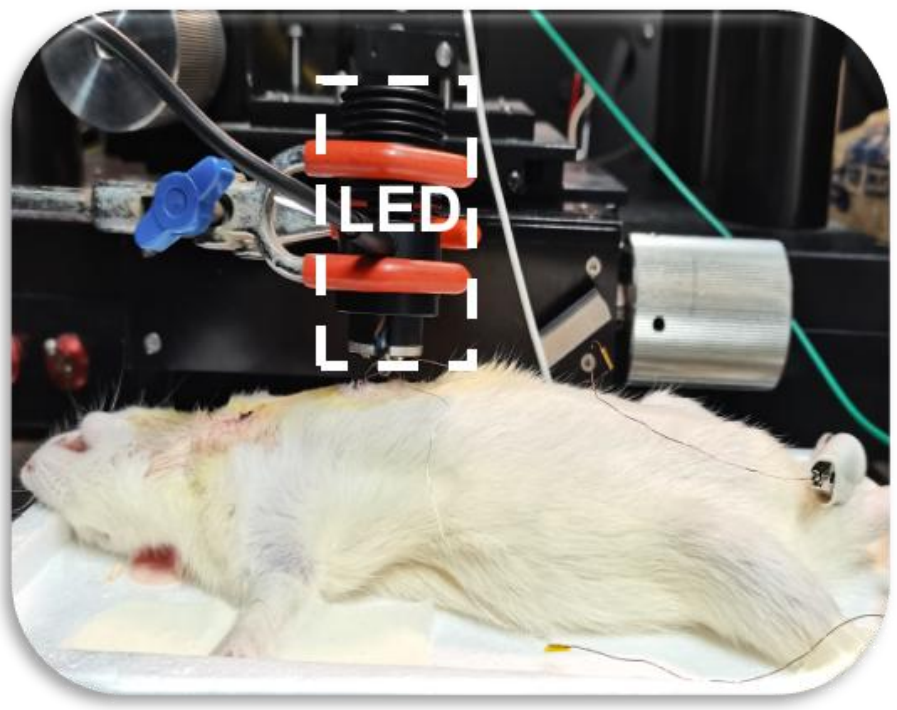

Fig. S16. Photographic image of *in vivo* implantation of the OES onto the myocardium of SD rats to evaluate wavelength-dependent photoresponsivity.

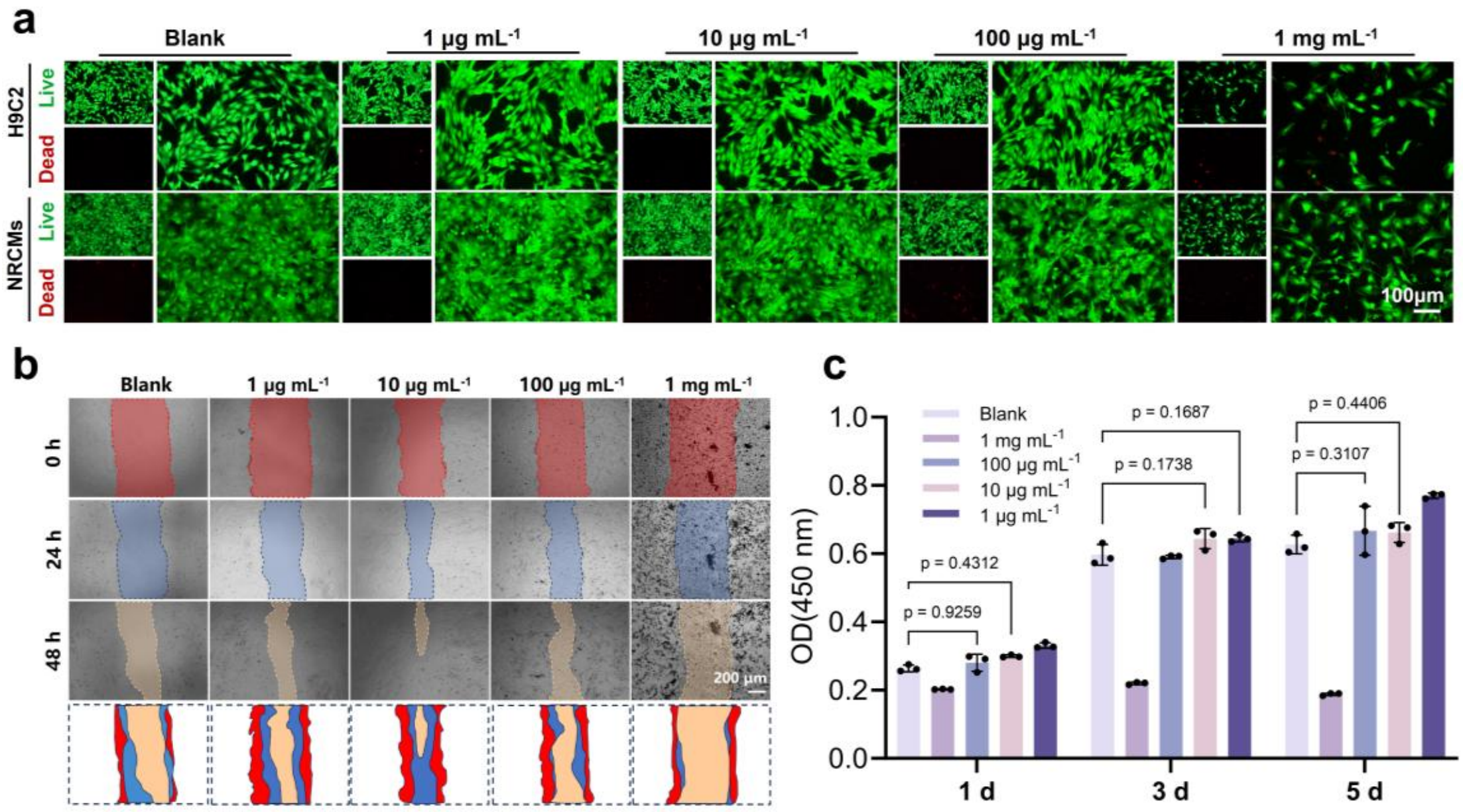

Fig. S17. ***In vitro* biocompatibility evaluation of OES.** (a) Live/dead staining of H9C2 cells and neonatal rat cardiomyocytes (NRCMs) exposed to low concentrations of Sb_2Se_3 (1, 10, and 100 μg mL^{-1}) showed no adverse effects on cell viability. (b) Cell migration assays indicated that Sb_2Se_3 at tested concentrations did not significantly alter the migratory behavior of H9C2 cells. (c) At high concentration (1 mg mL^{-1}), Sb_2Se_3 inhibited H9C2 cell proliferation, whereas lower concentrations (1, 10, and 100 μg mL^{-1}) maintained normal proliferative activity. The toxicity of antimony is mainly associated with its soluble ionic forms. The biological toxicity of Sb mainly comes from its soluble Sb compounds (e.g., Sb(III) salts). In this study, Sb is incorporated in a crystalline chalcogenide lattice $(Bi,Sb)_2Se_3$, which is chemically stable and exhibits extremely low solubility under physiological conditions[1]. Data in Fig. S17c are presented as mean ± S.D.; *n* = 3 independent experiments. Source data are provided as a Source Data file.

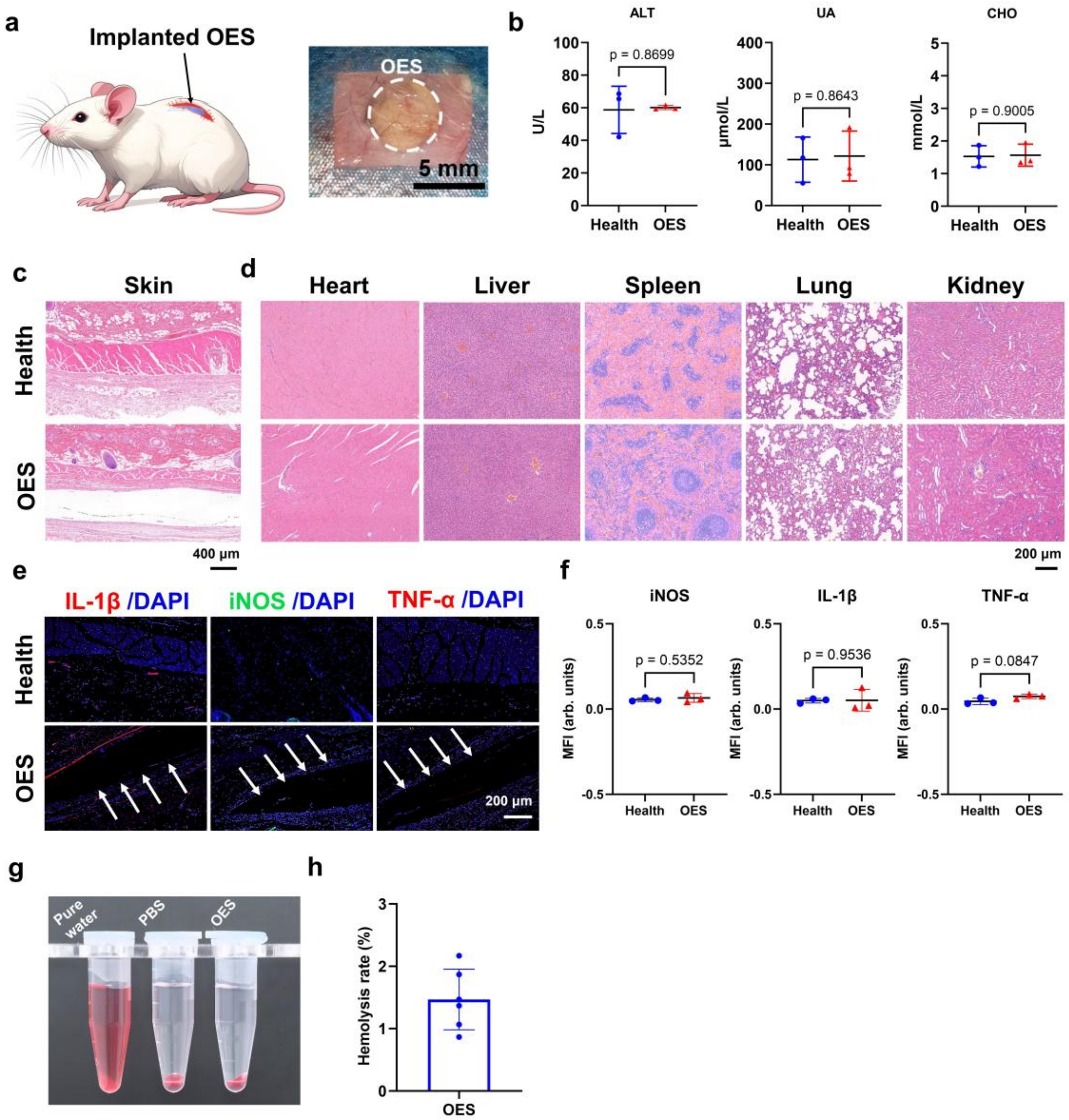

Fig. S18. *In vivo* biocompatibility evaluation of OES. (a) Subcutaneous implantation of OES discs (5 mm diameter) in SD rats shows no adverse effects on major organs. (b) Serum biochemical markers, including ALT, UA, and CHO, remain within normal ranges compared to healthy controls. (c) H&E staining of peri-implant tissue reveals minimal inflammatory infiltration. (d) Histological examination of major organs (heart, liver, spleen, lung, kidney) confirms absence of pathological changes one month post-implantation. (e, f) Immunofluorescence staining and quantification indicate no significant upregulation of inflammatory markers in the OES-implanted site compared to native skin. (g, h) Hemolysis results of OES. The OES exhibits negligible changes in the hemolysis ratio compared with the positive control. Data in Fig. S18b, f are presented as mean ± S.D.; n = 3 independent experiments. Data in Fig. S18h are

presented as mean ± S.D.; $n = 6$ independent experiments. Source data are provided as a Source Data file.

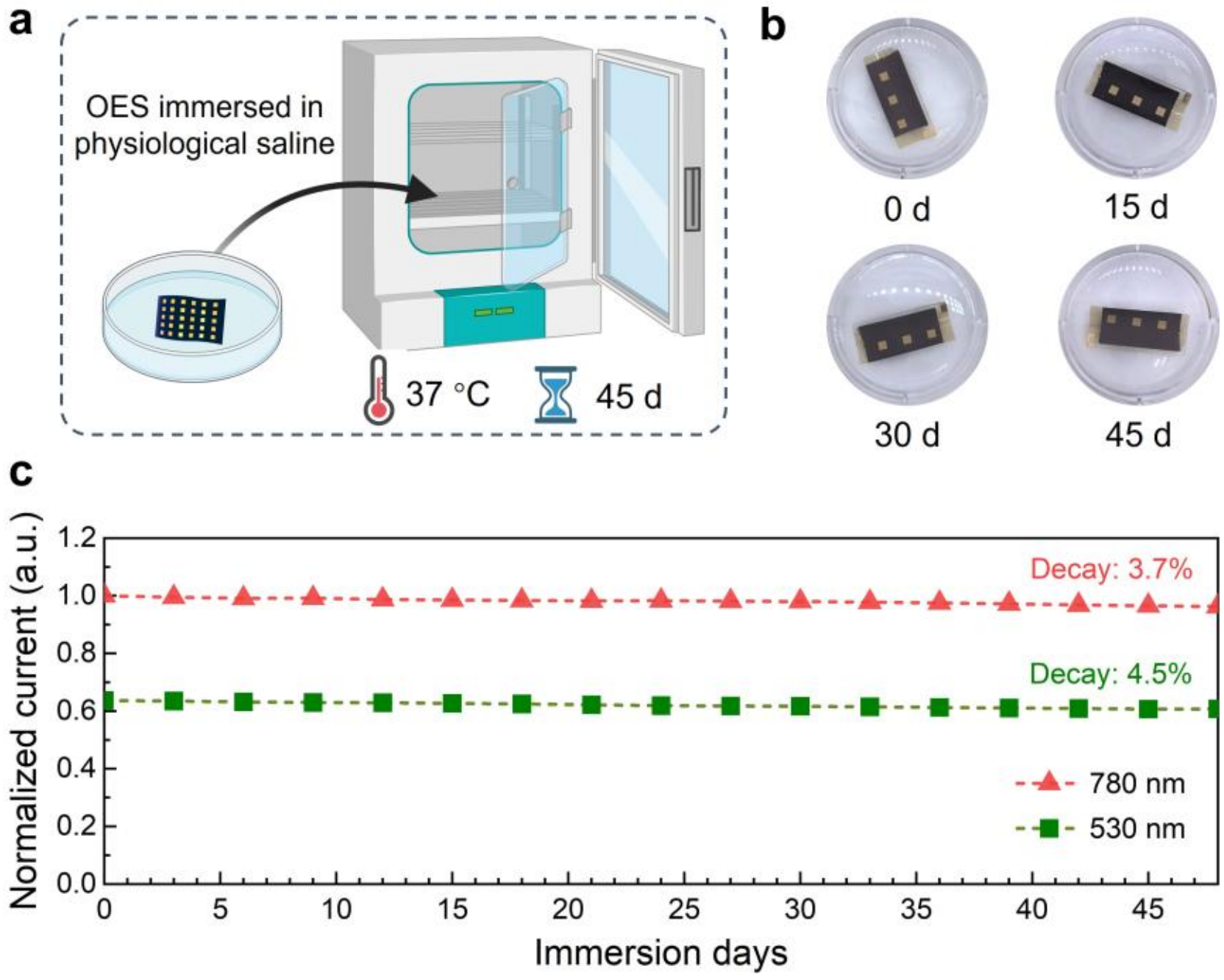

Fig. S19. (a) Schematic of the OES immersed in physiological saline at 37 °C for 45 days. Created in BioRender. ZHAO, R. (2026) https://BioRender.com/y3hfpkw. (b) Photographs of the OES after 0, 15, 30, and 45 days of immersion, showing nearly no observable change in appearance. (c) Photocurrent of the OES under 530 nm and 780 nm illumination under a light intensity of 24.7 mW cm^{-2}, with only 3.7% and 4.5% decreases after 45 days, respectively.

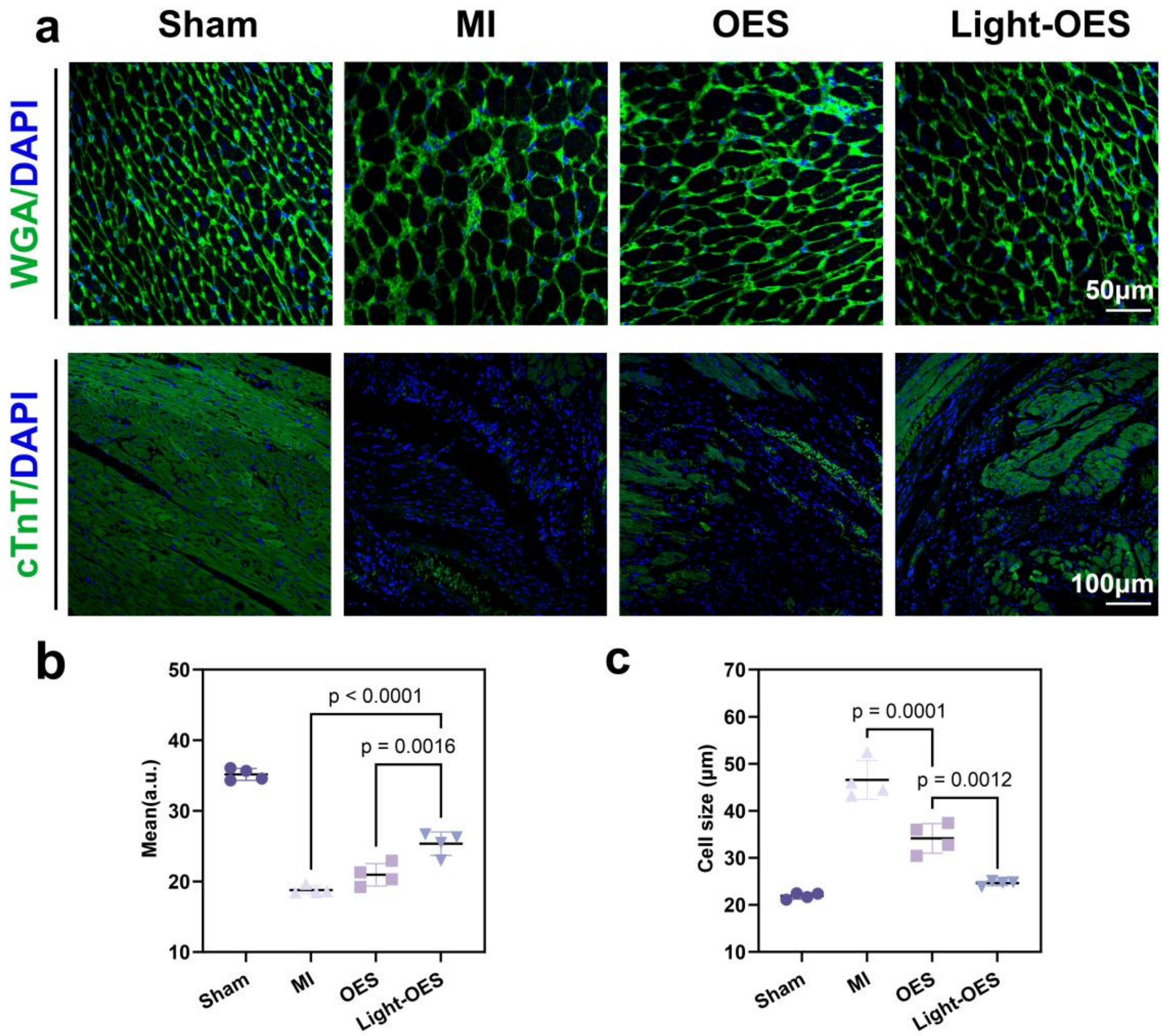

Fig. S20. Histological analysis of myocardial repair following OES treatment. (a) Representative immunofluorescence images of infarcted myocardium from Sham, MI, OES, and Light-OES groups, stained for wheat germ agglutinin (WGA; cell boundaries), cardiac troponin T (cTnT; cardiomyocytes), and nuclei (DAPI). (b) Quantification of cTnT-positive area based on immunofluorescence images. (c) WGA staining of the border zone highlights myocyte boundaries. Data in Fig. S20b, c are presented as mean ± S.D.; *n* = 4 independent experiments. Source data are provided as a Source Data file.

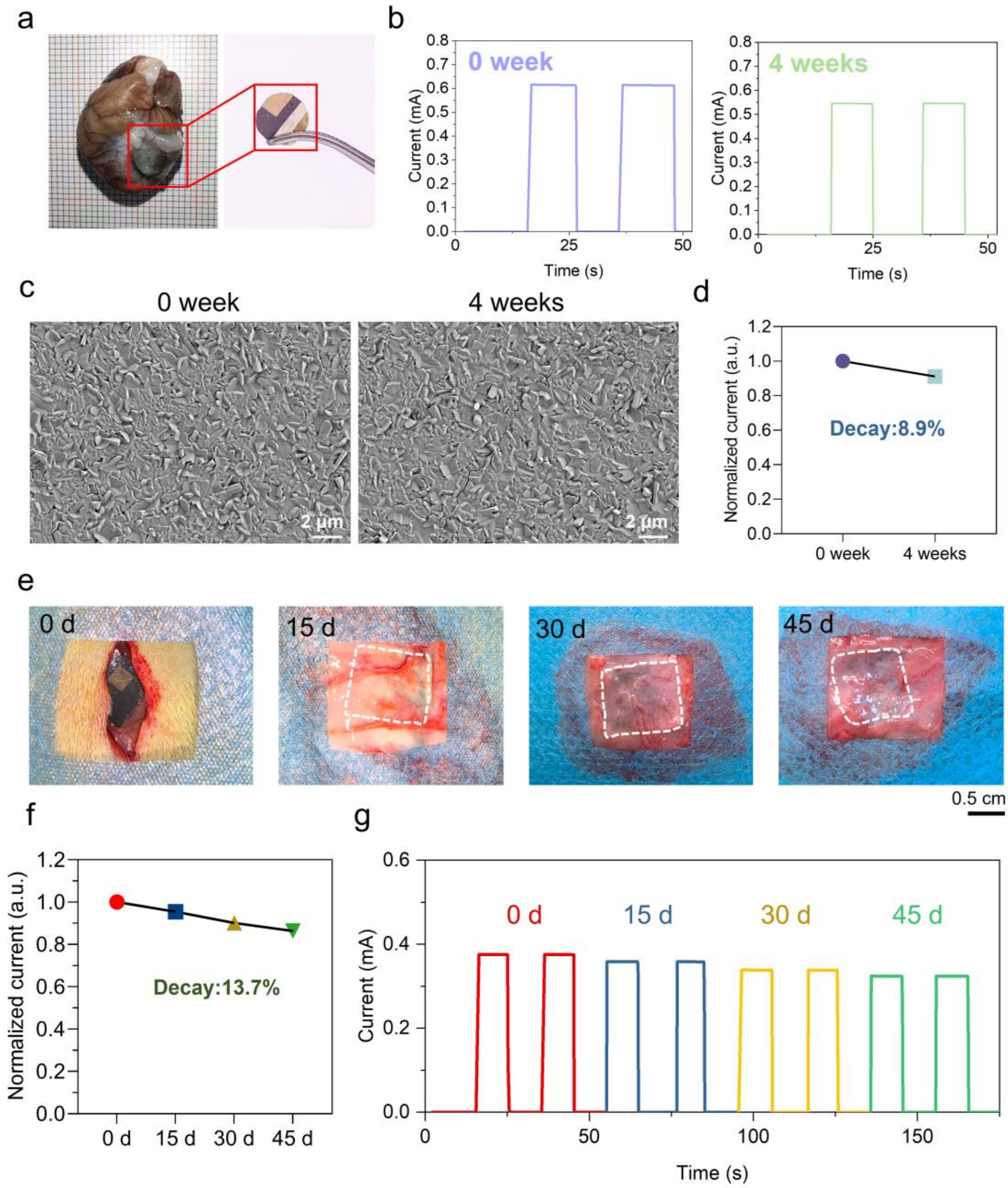

Fig. S21. Optical images of the OES retrieved after 4 weeks of implantation on the SD rat heart. (b) *I-T* curves recorded before and after implantation. (c) SEM images of the $(Bi,Sb)_2Se_3$ layers before implantation and retrieved after 4 weeks, indicating minor surface cracks without obvious delamination. (d) Photocurrent response of the OES after retrieval following 4 weeks of implantation. (e) Optical images of OES after subcutaneous implantation for 0, 15, 30, and 45 days. (f, g) Normalized photocurrent and *I-T* curves versus subcutaneous implantation time, under an illumination of 24.7 mW cm^{-2} at 780 nm.

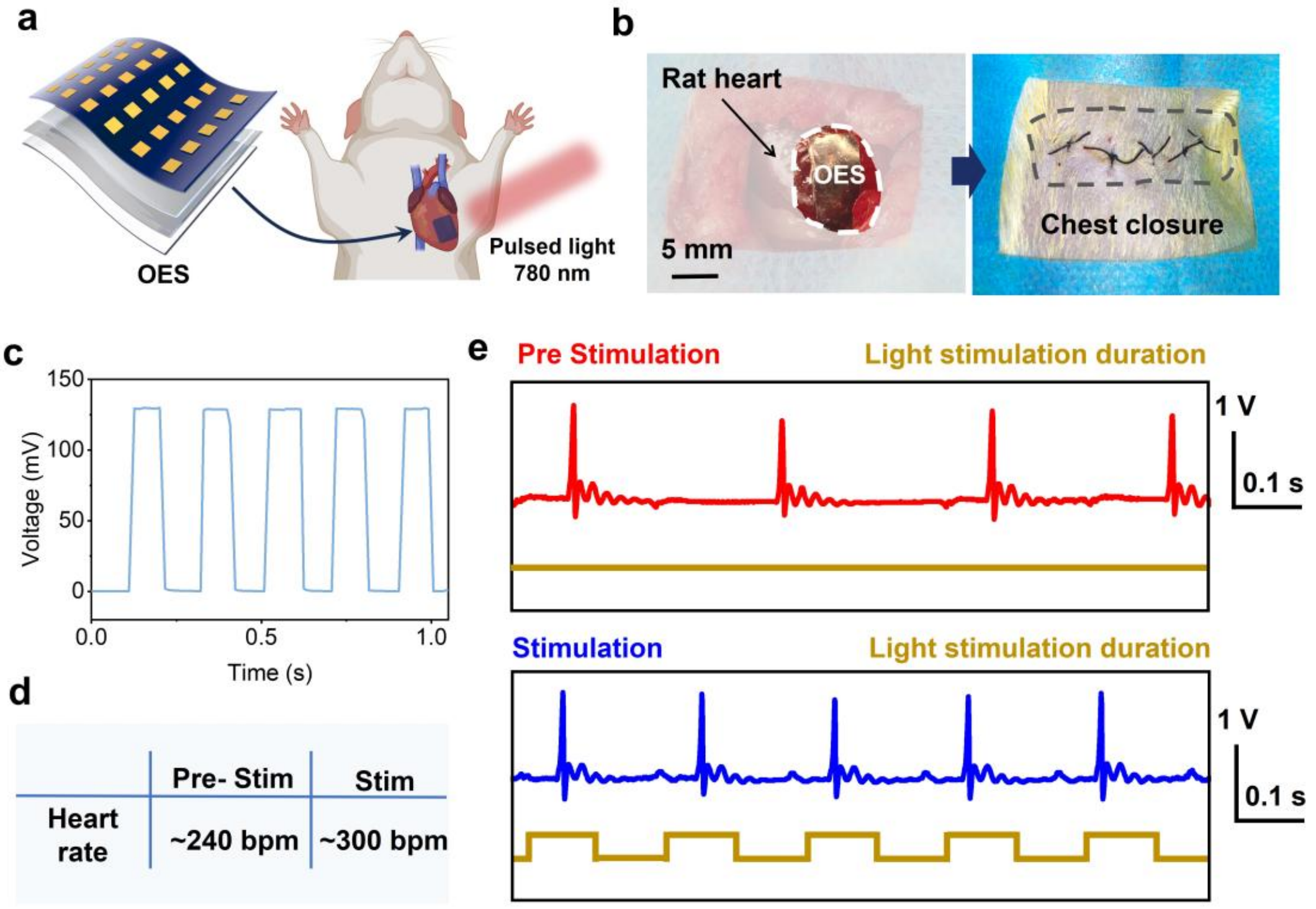

Fig S22. Closed-chest cardiac pacing enabled by the OES device in rats. (a) Schematic of the experimental setup illustrating OES-mediated cardiac pacing under closed-chest conditions, where externally delivered light is converted into electrical stimulation to modulate cardiac rhythm. Created in BioRender. ZHAO, R. (2026) https://BioRender.com/y3hfpkw. (b) Photograph showing the placement of the OES on the left ventricular surface of the rat heart for pacing experiments (device size: 5 mm × 10 mm). (c) Representative V_{OC} generated by the OES under optical stimulation at 5 Hz. (d) Quantification of heart rate before and during optical stimulation, showing an increase from ~240 to ~300 bpm. (e) Representative electrocardiogram (ECG) traces recorded from the rat during the pre-stimulation and stimulation periods.

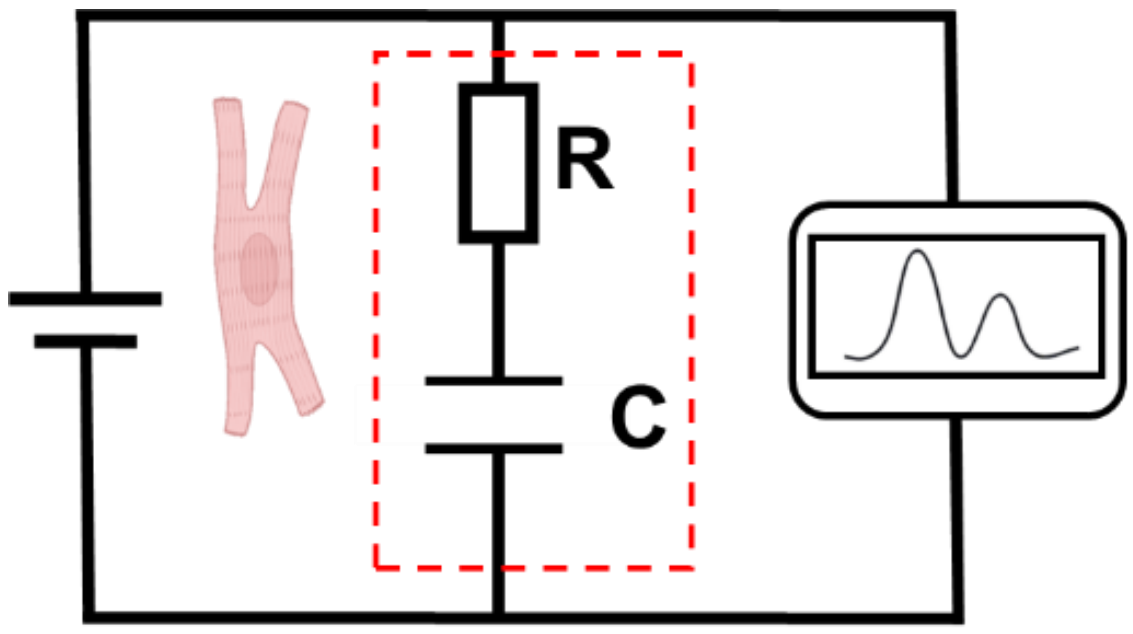

Fig. S23. Equivalent circuit model of the OES in contact with myocardial tissue. Created in BioRender. ZHAO, R. (2026) https://BioRender.com/y3hfpkw. The model simulates the voltage output of the OES at the tissue interface, incorporating representative parameters for tissue-electrode interaction (resistance R ≈ 2 kΩ, capacitance C ≈ 50 nF).

Supplementary References:

1 Yashina, L. V. *et al.* Negligible Surface Reactivity of Topological Insulators Bi_2Se_3 and Bi_2Te_3 towards Oxygen and Water. *ACS Nano* **7**, 5181-5191 (2013). https://doi.org/10.1021/nn400908b
