# Supplementary material for: Ultra-flexible optoelectronic stimulator converts tissue-attenuated weak light into electrical signals for cardiac remodeling
Source: Nat Commun. 2026 Jul 13;17:8622. doi: 10.1038/s41467-026-75495-7 (PMC13486475; doi:10.1038/s41467-026-75495-7)
Supplement: Supplementary file 4 [file 41467_2026_75495_MOESM2_ESM.pdf]

Description of Additional Supplementary Files

Supplementary Movie 1: Calcium transient imaging of primary cardiomyocytes cultured on the OSE surface under varying light intensities including Blank (No light), LPL (Low power light) and HPL (High power light).

Supplementary Movie 2: Calcium transient imaging of primary cardiomyocytes cultured on an interdigital electrode under varying electric field intensities of 0, 15, 32, 48 Vcm-2.

Supplementary Movie 3: Calcium transient imaging of primary cardiomyocytes cultured on an interdigital electrode under varying stimulation frequencies of 1, 2, 3 Hz.

Supplementary Movie 4: Establishment of the ischemic myocardial infarction model in SD rats.

Supplementary Movie 5: Implantatipon of OES onto the surface of infarcted myocardium in SD rats.

Supplementary Movie 6: Seamless adhesion of the flexible to the surface of the beating porcine heart.

Supplementary Movie 7: Effective performance of the OES under low-light conditions.
